# Network pharmacology and in vitro studies demonstrate modulation of fibrotic pathways by *Swertia chirayita* in pulmonary fibrosis

**DOI:** 10.1038/s41598-025-30784-x

**Published:** 2025-12-08

**Authors:** Bharath H. B., Farmiza Begum, Gautam Kumar, Jyothi Giridhar, Usha Y. Nayak, Fayaz S. M., Pawan Ganesh Nayak, Yogendra Nayak

**Affiliations:** 1 Department of Pharmacology, Manipal College of Pharmaceutical Sciences, Manipal Academy of Higher Education, Manipal, Karnataka 576104 India; 2 Department of Pharmacology, Vaagdevi Pharmacy College, Bollikunta, Warangal, Telangana 506005 India; 3 Amity School of Pharmaceutical Sciences, Amity University, Mohali, Punjab 140306 India; 4 Department of Pharmaceutical Chemistry, Manipal College of Pharmaceutical Sciences, Manipal Academy of Higher Education, Manipal, Karnataka 576104 India; 5 Department of Pharmaceutics, Manipal College of Pharmaceutical Sciences, Manipal Academy of Higher Education, Manipal, Karnataka 576104 India; 6 Department of Biotechnology, Manipal Institute of Technology, Manipal Academy of Higher Education, Manipal, Karnataka 576104 India

**Keywords:** Fibroblast proliferation, Immunofluorescence, TNF pathway, Principal component analysis, Epithelial mesenchymal transition, Computational biology and bioinformatics, Diseases, Drug discovery

## Abstract

Various biological processes contribute to pulmonary fibrosis, which results in fibrotic foci that impede the exchange of gases between alveoli and capillaries. This study investigates the therapeutic mechanism in fibrotic foci reconfiguring via the TNF signaling pathway by potential *Swertia chirayita* (SC) components to treat pulmonary fibrosis by network pharmacology, in silico, and in vitro studies. The targets of LC-MS/MS analyzed SC components were used to build a protein-protein interaction network in Cytoscape and predicted key targets and signalings. The molecular docking, dynamics (MD) simulation, and principal component analysis (PCA) were performed to predict the potential interactions between components and their targets. In vitro studies, such as cell migration, E-cadherin immune fluorescence assay, and western blot analysis were performed in NIH3T3 and A549 cells with or without TGFβ1 stimulation. The network pharmacology of SC revealed nine key targets, and the pathway analysis found that these targets are implicated in TNF-α signaling. The main components of SC have substantial binding affinities, as shown by molecular docking. Furthermore, MD simulation and PCA predict that bellidifolin, gentiopicroside, and mangiferin will substantiate the anti-fibrotic effect. SC inhibited fibroblast migration and differentiation, epithelial to mesenchymal transition, and TNF-α downstream markers like NF-κB/p-NF-κB. In vitro studies confirmed the network pharmacology and docking predictions that SC can modulate fibrotic foci by acting through the TNF-α signaling pathway.

**Supplementary Information:**

The online version contains supplementary material available at 10.1038/s41598-025-30784-x.

## Introduction

 The primary cause of idiopathic pulmonary fibrosis (IPF) progression is the dysfunction of alveolar epithelial cells, which is caused by genetic, epigenetic, or environmental changes in gene expression. These changes set off a cascade of processes that include mechanotransduction, endoplasmic reticulum (ER) stress, mitochondrial dysfunction, cell integrity, epithelial-mesenchymal transition (EMT), cellular senescence, fibroblast proliferation, differentiation, and extracellular matrix (ECM) deposition^1–3^. The transforming growth factor-beta (TGF-β) and tumor necrosis factor-alpha (TNF-α) signaling are considered major regulators of these complex pathogenic processes resulting from epithelial cell injury. TNF-α is responsible for the early expression of lung injury from macrophages, fibroblasts, and T cells in the alveoli^4^. TNF-α signaling has been shown to play a significant role in the formation of fibrotic foci, along with the induction of oxidative and nitrosative stress, as well as necrosis, apoptosis, angiogenesis, and tissue remodeling^5^. The abnormal expression of TNF-α is also involved in initiating the inflammatory cascade. Through its paracrine impact, TNF-α released by fibroblasts enhances the focal concentration of fibroblasts^6^. The activation of TNF-α signaling regulates NF-κB and MAPK downstream signaling pathways in the myofibroblast differentiation from the focal and activated fibroblasts. It leads to interstitial inflammation and the development of fibrotic foci with the deposition of the ECM, which are the final results of TNF-α signaling activation^7^. The dysregulation of epithelial cell injury repair and alveolar type II (ATII) to type I (ATI) transdifferentiation alters alveolar homeostasis and promotes epithelial injury, which is another significant mechanism that triggers EMT and fibroblast proliferation^8^. Since TGFβ1 is the primary profibrotic marker, it largely promotes EMT, fibroblast-to-myofibroblast differentiation, and chronic inflammation^3,9^. The formation of fibrotic foci involves complex, multiple processes with numerous target interactions; hence, targeting multiple targets with multiple components could be a promising therapeutic approach.

The US FDA approved nintedanib and pirfenidone for the treatment of IPF. Nintedanib and pirfenidone are specific anti-fibrotic drugs regulating tyrosine kinase receptors (EGFR, VEGFR, FGFR, and PDGFR) and TGFβ^10,11^. Pirfenidone has additional antioxidant and anti-inflammatory activity that acts by modulating TNF-α and interleukins^10,12^. However, both of these drugs have adverse effects and do not provide a complete effect; they slow the progression and extend the life of IPF patients for a few more years^13,14^. The current limitations of the therapeutic approach urge alternative treatment with minimal adverse effects. Traditional and natural medicines with multiple components provide greater insight into exploring therapeutic approaches with minimal secondary effects. *Swertia chirayita* (Roxb.) Buch.-Ham. ex C.B.Clarke (SC), (Fam: Gentianaceae), is one such traditional Indian medicinal herb used as a tonic in Unani and Ayurvedic therapies to treat respiratory conditions such as bronchitis and asthma, meningitis, inflammatory diseases, and other ailments^15,16^. In the traditional Chinese and Tibetan medicinal systems, SC was used to treat inflammatory diseases^17^. SC phytoconstituents primarily comprise triterpenoids, lignans, xanthones, and secoiridoid glycosides. Mangiferin (MANG) and gentiopicroside (GENPD) have been shown to have an anti-fibrotic effect in preclinical studies for treating pulmonary fibrosis by regulating inflammatory markers (TNF, IL-17, and IL-6) and TGFβ1/Smad and TNF-α/NF-κB signalings^18–20^. Another component, bellidifolin (BELDF), was reported to aid in the reduction of α-SMA and Collagen I and III production in myocardial fibrosis and may prove to be a useful treatment for pulmonary fibrosis through TGFβ1 signaling^21^. The rich composition of SC and its traditional uses lend strength to treating pulmonary fibrosis through a holistic effect.

The mechanism of action of natural products is more complex to explain than that of modern medicines. Network pharmacology elucidates the mechanism of action of traditional medicines in treating diseases with complex pathologies^22^. It employs an interdisciplinary and comprehensive analytical approach to comprehend complex mechanisms. Network pharmacology as an exquisite integrative research method that decodes a complex network and looks at prominent targets for SC in order to comprehend the intricacy of IPF advancement^23^. The application of network pharmacology investigates SC constituents and their molecular-level interactions, multi-target effects, potential efficacy, and the pathways influenced by these targets in pulmonary fibrosis. This study provides a mechanism by which SC acts as an anti-fibrotic agent to treat pulmonary fibrosis. The compound-target interactions were validated using molecular docking and dynamics simulation as an evidence-based approach to support the study findings. The computational predictions were confirmed by evaluating the effectiveness of traditional herbs in cell culture studies on A549 and NIH3T3 cells. The TGFβ1 stimulation of A549 and NIH3T3 cells mimics the EMT and fibroblast proliferation and differentiation of myofibroblasts. The anti-fibrotic effect of SC, as proposed by network pharmacology, was investigated through cell migration and E-cadherin immunofluorescence assays to confirm the inhibition of fibroblast migration, differentiation, and EMT. The effective pathway regulations by SC were confirmed in fibroblast proliferation and EMT inhibition, which are involved in the formation of fibrotic foci in the interstitial space through multimodal interactions.

## Materials and methods

### Collection, authentication, and extraction of *Swertia chirayita*

The SC was procured from an authentic herb seller, ‘Trust the Herb’ (https://trustherb.com), as an entire dried plant. The authentication certificate was obtained from Mrs. Shruthi Nayak, Head of Department of Botany, Mahatma Gandhi Memorial College in Udupi, Karnataka, India. The voucher specimen was prepared and deposited in the Herbarium at the Department of Pharmacology, Manipal College of Pharmaceutical Sciences, Manipal Academy of Higher Education, Manipal (# 2025001). The dried herb was made into a coarse powder using an electric blender. The powdered SC was weighed to 400 g and packed accordingly in a Soxhlet apparatus with ethanol (22072000-Zenith chemicals) to macerate for 2 h. Ethanol was added to the boiling flask to collect the extracts from the powdered plant. The cycle was then started every day for twelve hours for five days, with the heat set to 40 °C. Following extraction, the solvent-containing extract was transferred to a second round-bottom flask with a rotary evaporator to remove the solvent under reduced pressure and temperature. The yield of SC extract was 34.83 g. It was stored at −80 °C freezer and then in desiccator for further screening and assessment of its efficacy^24^.

### Liquid chromatography-tandem mass spectrometry (LC-MS/MS Q-TOF) for component identification

The plant extract was subjected to Liquid chromatography-tandem mass spectrometry (LC-MS/MS Q-TOF) using the UPLC ACQUITY H CLASS Series separation system with C18 Waters, Acquity BEH 2.1*100 mm 1.7 μm liquid chromatography column with gradient mobile phase of solvent A (0.1% formic acid (533002-Merck) + LC-MS grade water) and solvent B (0.1% Formic Acid + Acetonitrile (6183002500-Merck)) at 0.2 ml/min flow rate for 45 min, and the flow was directed to the MRM (unit resolution). The mass spectrometer was optimized to 950 Lts/h desolvation gas flow, 120 °C source temperature, 3.22 keV capillary voltage, 50 V cone voltage, 4 eV collision energy, and 80 V source offset to perform in the positive mode ([M + H]+), a quadruple and time-of-flight analyzer was used for running MS modes^25^.

### Protein-protein interaction and target identification

The number of active components reported and present in the SC were identified from the LC-MS/MS analysis of the ethanolic extract. SuperPred generates a list of possible targets for the compounds identified in the LC-MS/MS analysis using machine learning models and similarity scores based on the structural features of compounds^26^. ‘GeneCards’ and ‘DisGeNET’ were used to locate the targets with disease targets involved in IPF using the efficient keyword ‘Idiopathic pulmonary fibrosis or Lung fibrosis’^27^. The common overlapped targets between SC and the disease were identified. A protein-protein interaction (PPI) network was built for the overlapped (common) targets using the interaction network analyzer Cytoscape 3.10.2 software with the string protein query built-in application, considering a 0.90 confidence score, *homo sapiens* species, and no further extra interactions^28^. The built network was analyzed using a built-in analyzer tool to calculate the topological parameters. Cytohubba was utilized to identify and create separate sub-networks of each parameter. Hub targets were obtained by analyzing the degree, betweenness, and closeness centrality of the PPI network. Targets to select for the molecular docking further screening were performed for hub targets with string disease targets of IPF. IPF-specific targets were enriched with confidence scores of 0.9 in Cytoscape and analyzed with the topological parameter. The IPF targets with the top 30-degree targets network merged with hub targets network of SC to identify major targets for docking using the intersection criteria^29^.

### Enrichment analysis and compound-target-pathway (C-T-P) network

The overlapped SC compounds and disease targets were uploaded to the Database for Annotation, Visualization, and Integrated Discovery (DAVID) database to predict the complete biological annotations with the complete targets interactions^30^. The bioinformatic analysis was performed with GO (Gene Ontology) and KEGG (Kyoto Encyclopedia of Genes and Genome) pathways (permission obtained with Ref:252218)^31^. The top 10 enriched pathways and top 5 biological processes (BP), molecular function (MF), and cellular components (CC) were considered based on their fold enrichment, count, and p-values specifically involved in the IPF disease progression. The top 10 KEGG pathways involved in the disease were considered to build a C-T-P network with the existing compound and target interaction network.

### Molecular docking

All the molecules of SC were docked with the primary targets identified through network pharmacology, and PDB IDs were imported from the RCSB Protein Data Bank. The Glide module of the Schrodinger software was utilized to dock the protein and ligands. Using the Maestro Protein Preparation Wizard and LigPrep, the protein structures and ligands were optimized^32^. In order to build a glide grid for proteins without ligands, a site map was run in addition to the direct glide grid generated for the co-crystallized proteins^33^. The conformation with the lowest binding affinity and the essential interactions was considered the best docking result after running the XP-docking (extra precision) of the Glide module^34^. The analysis of several interaction scores, such as the hydrogen bond score, lipophilic interaction, and electrostatic rewards, was done using an XP-visualizer.

### Induced fit docking

Based on the docking analysis, the selective ligands and proteins were subjected to induced fit docking (IFD). The IFD dock score for the various poses created was computed, and binding site interactions with the ligand were examined^35^.

### Molecular dynamics simulation

To investigate the protein-ligand complexes conformational stability and steady state, 100 ns molecular dynamics (MD) simulations were performed for the good protein-ligand interactions shown in the XP-docking and IFD pose. The Desmond module was utilized in our study to perform MD simulations^36^. By using SPC solvent and maintaining iso-osmolarity in the system builder, the Protein-ligand complex was prepared^37^. The system was minimized and relaxed to a minimum local energy of 100 ps. Utilizing an isotropic style for 100 ns using NPT, the Nose-Hoover chain thermostat and Martyna-Tobias-Klein Barostat technique were coupled to a 300 K temperature and 1.0315 bar pressure for the MD simulation^38^. Root mean square deviation (RMSD), root mean square fluctuation (RMSF), hydrogen bonding, hydrophobic interactions, radius of gyration (rGyr), and Solvent Accessible Surface Area (SASA) were calculated for every protein-ligand complex using the simulation interaction diagram (SID).

### Principal component analysis (PCA)

In order to compute eigenvectors and eigenvalues, the PCA was utilized to assess the dynamic motion of the protein impacted by ligand binding with the respective proteins. The Desmond simulation trajectory was converted to carry out PCA using the MD analysis^39,40^. Domain cross-correlation map (DCCM) was carried out using Schrodinger’s suite. It shows the residue displacement correlation, both positive and negative, throughout the simulation. Here, the mobility of correlated and noncorrelated protein-ligand complexes is estimated^41^. PCA projections were converted to calculate relative free energy by using Matplotlib for static 3D and 2D plots, contour maps showing energy minima (stable states) and energy barriers.

### Cytotoxicity assay on NIH3T3 and A549 cells

The NIH3T3 fibroblast and A549 epithelial cell lines were procured (Req. No. 1882/2023–2024) from the National Centre for Cell Science (NCCS), Pune, India. All the experiments involving NIH3T3 and A549 cells were performed as per the guidelines of the Institutional Biosafety Committee-Manipal Academy of Higher Education (Reg. No. F.9.8–89/U.3). The sulforhodamine B (SRB) assay was performed to determine the cytotoxicity of SC. Flasks with NIH3T3 and A549 cells were sub-cultured, and a stock of 1 × 10^5^ cells/ml was prepared in 10% DMEM (11995-065-Gibco) media. Incubated the 96-well plate for 24 h, containing 100 µl/well. Cells were treated at different concentrations of SC (A549 and 3T3: 1000–15.625 µg/ml) and nintedanib (3T3: 50–3.125 µg/ml and A549: 125–15.625 µg/ml). Based on the reported IC₅₀ values of nintedanib on A549 and NIH3T3, appropriate concentration ranges were selected^42,43^. After 24 h of treatment exposure, the plates were observed under the microscope, and the cells were fixed to the plates using 10% trichloroacetic acid (TCA) (60677-SRL) for 1 h at 4 °C. After the specific incubation, the plates were washed with tap water four times. The air-dried plates were treated with 100 µl of 0.04% SRB (230162-Sigma) solution in each well, incubated for 1 h at RT, and washed with 1% v/v acetic acid (695092-Sigma). 100 µl/well of a 10 mM Tris base (MB029, Himedia) solution (pH 10.5) was added to a dried plate and kept under shaking conditions in an orbital shaker for 10 min. The absorbance was recorded at 540 nm using a microplate reader^44^.

### TGFβ1-induced fibroblast proliferation and differentiation

NIH3T3 cells were seeded in 12-well plates with 10% DMEM media and incubated for 24 h. Then, the cells were incubated for 12 h with serum-deprived (1% FBS (10270-106)) media. After incubation, scratches were made with a 1 ml pipette tip and washed with PBS to remove cell debris. Cells were treated with SC (110 µg/ml) and nintedanib (4 µg/ml) (FDX210012-Cipla) with or without TGFβ1 (100-21-Imperial Life Science). The scratch area was photographed using 40X magnification to consider a 0 h (initial) time point. Cells were incubated for 24 h, and the scratch area was photographed. Image J software was utilized to represent the percentage migration of fibroblast cells from 0 h to 24 h^45,46^.

### SC inhibits TGFβ1-induced EMT in A549 cells (immunofluorescence assay)

A549 cells were seeded onto poly-l-lysine (P1524-Sigma) coated coverslips and incubated for 24 h to attach. Cells were treated with SC (180 µg/ml) and nintedanib (30 µg/ml) with or without TGFβ1 and incubated for 24 h. Cells were fixed by adding 4% paraformaldehyde (TC703-Himedia), permeabilized with 0.1% Triton X-100 (MB031-Himedia) for 10 min each, and blocked with 3% BSA (TC194-Himedia) for 1 h at room temperature (RT). Cells were incubated with E-cadherin (sc-8426-Santa Cruz) antibody overnight at 4 °C. Coverslips were washed and incubated with FITC (fluorescein isothiocyanate)-conjugated secondary antibody (A18916, Thermofisher) (488 nm) for 1 h at RT. Coverslips were washed and incubated with three µg/ml DAPI (18668-SRL) for 10 min to stain the nuclei and then washed with PBS. Coverslips were fixed to the slides with mounting media. The slides were dried for a few minutes and sealed. The slides were stored in the dark at 4 °C and visualized under a fluorescent microscope with filters of FITC (green, excitation 488 nm, emission 520 nm) and DAPI (blue, excitation 358 nm, emission 461 nm). The intensity of E-cadherin was measured for 127x photographs using ImageJ bundled with 64-bit Java 8 software (ImageJ 1.54 g; Java 1.8.0_345) downloaded from https://imagej.net/ij/^47^.

### Western blot analysis

A549 and NIH3T3 cells were seeded in 60 mm dishes at a 3 × 10^5^ cells/dish density and incubated for 24 h. After incubation, cells were treated with SC (A549- 180 µg/ml and NIH3T3- 110 µg/ml), and nintedanib (A549- 30 µg/ml and NIH3T3- 4 µg/ml) for 24 h along with or without TGFβ1 (4 ng/ml). Protein extraction was done using Radio-Immunoprecipitation Assay (RIPA) buffer and centrifuged at 14,000 g for 20 min at 4 °C for supernatant. Total protein contents were determined in the supernatant using a BCA protein assay kit (71285-3-Sigma) to add an equal amount (30 µg) to every well. Proteins were prepared in the sample buffer (4:1) along with 2-mercaptoethanol (21985023-Sigma) and denatured by heating for 5 min at 95 °C. Samples were loaded onto each lane with a protein ladder (1610374-Biorad) and separated on 8 and 10% SDS polyacrylamide gels (1610156-Biorad) at 50 V. Followed by separated bands on the gel, they were transferred to PVDF (10600023-Cytivia) membranes at 90 V. Membranes were blocked with 3% BSA at RT and incubated with primary antibodies (EGFR (E-AB-31281-Elabscience), NF-κB (PA5-16545-Thermofisher), p-NF-κB(MA5-15160-Thermofisher), β-actin (MA1140-Thermofisher)) overnight at 4 °C. The blots were washed and then incubated with HRP-conjugated secondary antibody (anti-mouse:31450-Thermofisher and anti-rabbit: E-AB-1003-Elabscience) for 2 h on a shaker at RT. After incubation, the blots were washed, and finally, the peroxidase activity was visualized using the ECL (T7103A-Takara) solution in the G: box visualizer^48,49^. After the visualization, blots were undertaken for the mild stripping using the stripping buffer, and the process was continued in the same manner from the blocking step for the visualization of the next protein. B-actin was used as a loading control for the models of pulmonary fibrosis. The blots were further analyzed for protein expression using ImageJ bundled with 64-bit Java 8 software (ImageJ 1.54 g; Java 1.8.0_345) downloaded from https://imagej.net/ij/.

### Statistical analysis

Experimental data were presented as the mean ± SEM, and the statistical analysis results were plotted using GraphPad Prism 8.0.2. All experiments were conducted in triplicate. In the event of significant differences among groups, multiple comparisons were conducted using Tukey’s test.

## Results

### LC-MS/MS analysis for *Swertia chirayita* components analysis

**Fig. 1:**
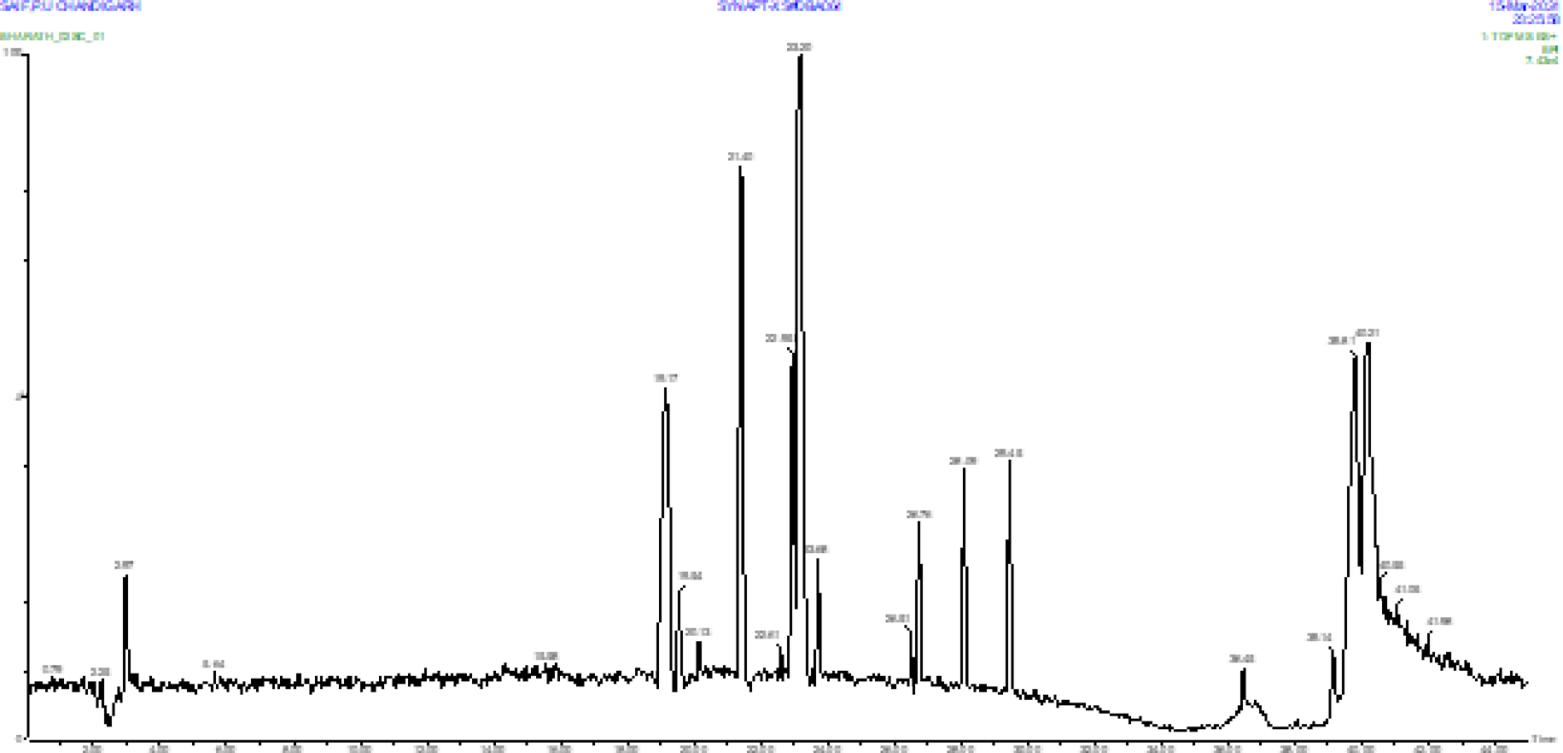
LC-MS/MS chromatographic profile of *Swertia chirayita* extract depicting metabolite peaks: components identified at represented retention time peaks.

The LC-MS/MS Q-TOF showed 35 phytochemicals in the SC ethanol extract. The obtained mass spectra graphs were compared with the registered mass spectrometry database information from the NIST Chemistry WebBook, Human Metabolome Database (HMDB), and PubChem (National Library of Medicine)^50,51^. Several m/z were identified in the SC extract, as listed in Table 1, chromatogram peaks in Fig. 1, and mass spectra graphs were depicted in supplementary file S1, 1–11.

**Table 1:** LC-MS/MS identified *Swertia chirayita* whole plant ethanolic extract components.

*Swertia chirayita* components	Molecular weight	Reported fragment/precursor	[M + H]^+^/MS fragment ion (m/Z)	Retention time (min)
Dl-aspartic acid	133.1	134, 116, 88, 74	134.07	2.937
Chiratenol	426.39		427	19.197
Enicoflavine	211.21		211.15	19.197
Gentianine	175.18	176, 158, 133, 105, 145	133.10, 145.10, 105.07, 176	19.197
L-leucine	131.17	86, 132	132, 85	19.197
L-threonine	119.12	76, 120	119.09	19.197
Palmitic acid	256.4	257.2	257.15	19.197
Stearic acid	284.5	285.26	285.19	19.197
Syringaresinol	418.4	401, 369, 388, 265, 180	419.19	19.197
Taraxerol	426.7	427, 409, 353	353	19.197
(3E)−3-(aminomethylidene)oxane-2,4-dione	141.12		140.92	19.568
Glutamate	147.13	84, 130, 102, 56	84.95	19.568
Bellidifolin	274.2	191, 219, 109, 275, 247	191.11	21.371
Chiratol	288.25		289.21	21.371
Demethylbellidifolin	260.2	261, 219, 81, 127, 243, 153, 189, 233	260.12, 219, 81	21.371
Dl-tryptophan	204.22	205	205.12	21.371
Swerchirin	288.25	288, 273	287.2	22.95
Stigmasterol	412.7	255, 159, 83, 97, 55, 413	159	21.371
5,8-dimethylbellidifolin	145.2		145.1	23.155
Amaroswerin	602.5	227, 229, 247, 603, 201, 203	227.11, 203, 201	23.155
Calendol	426.7	427, 409, 353	352.17, 353	23.155
Swertiamarin	374.34	375, 355, 195, 177, 213, 149, 167, 119	355.18, 119, 375	23.155
Amarogentin	586.5	391.1, 229.1, 198	198.94	23.715
Pichierenyl acetate	468.8	468	468	26.76
2,5-Dihydroxyterephthalic acid	198.13		198.94	26.76
Octadecanoate	283.5		284	28.094
Sweroside	358.34	127, 197, 151, 111, 179	359.11	28.094
Decussatin	302.28	303, 287, 259, 271, 301	303, 301.2	36.45
Swertinin	414.7		415.5	36.45
Erythrodiol	442.7	407, 443, 425, 395	443	39.105
Gentiopicroside	356.32	149, 121,195, 177	149.13, 121, 177	39.105
Mangiferin	422.3	273, 303, 339, 256, 423, 405	303.21, 339.34, 406	39.105
Swertiapuniside	598.5		599.42	39.105
Beta-Sitosterol	414.7	414, 397, 379, 119	397.37, 119	39.105
Isovitexin	432.4	433, 434, 313	433.17, 313	39.105

### Identification of the disease targets and targets for the compounds found in the extract

Overall, 416 potential disease targets were identified in IPF. It was found that 802 targets interacted with the SC components, as shown in the supplementary file S2-1 and S2-2. The duplicate conditional formatting displayed 116 common overlapping targets between the disease and SC targets, as represented in the supplementary file S2-3.

### Protein-protein interaction (PPI) network

**Fig. 2:**
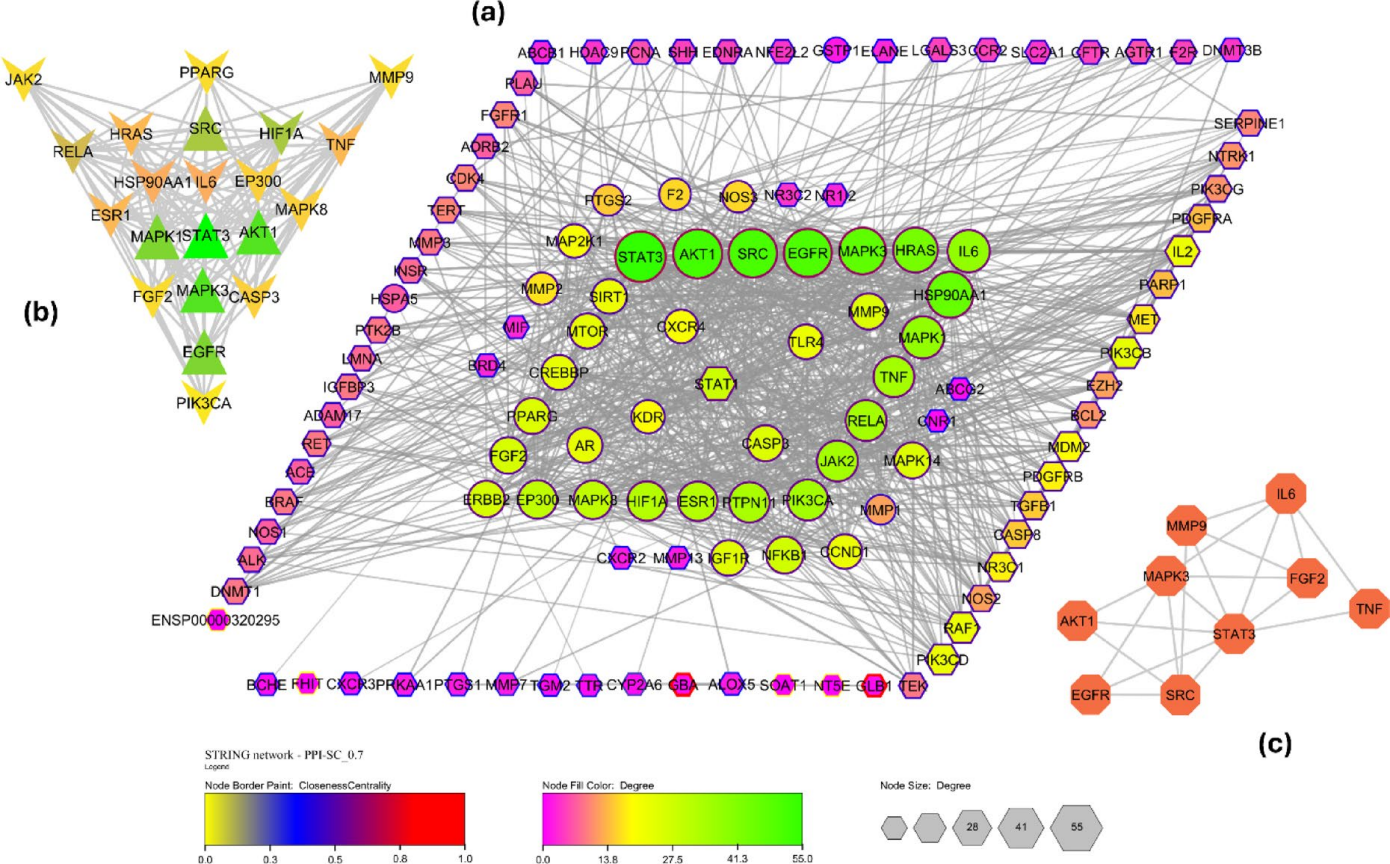
Cytoscape networks (**a**) Protein-protein interaction network for SC components in pulmonary fibrosis. Node size and color represent degree (green > yellow > purple), node border color represents closeness (red > blue > yellow), and node shape represents betweenness (Circular > hexagon). (**b**) Hub targets comprise all three degrees, betweenness, and closeness centrality. (**c**) Targets identified for the molecular docking based on the topological screening.

Figure 2a shows the PPI network of 116 common targets that was constructed with a string protein query. Supplementary file S3-2 highlighted the top 30 hub targets in terms of the betweenness, degree, and closeness centrality criteria. Considering all three major centrality measures, a network of 21 hub targets (Fig. 2b) was obtained in Cytoscape by merging all hub networks using the intersection criteria. These targets were found to possess good topological characteristics, primarily in terms of degree, betweenness, and closeness centrality. Similarly, a disease IPF-specific protein-protein interaction network (DOID: 0050156) was built and the hub targets were identified as mentioned in the Supplementary file S3-1 based on the topological parameters. After merging the Fig. 2b hub network and Supplementary file S3-1 disease hub network in Cytoscape, a nine-core target network (Fig. 2c) was obtained after prioritizing the overlapping nodes. The nine core targets identified—TNF, MMP9, IL-6, AKT1, MAPK3, STAT3, SRC, EGFR, and FGF2—were considered for molecular docking, as they are highly involved in pulmonary fibrosis progression, rather than examining 30 targets.

### Functional annotations: pathway enrichment analysis and gene ontology

**Fig. 3:**
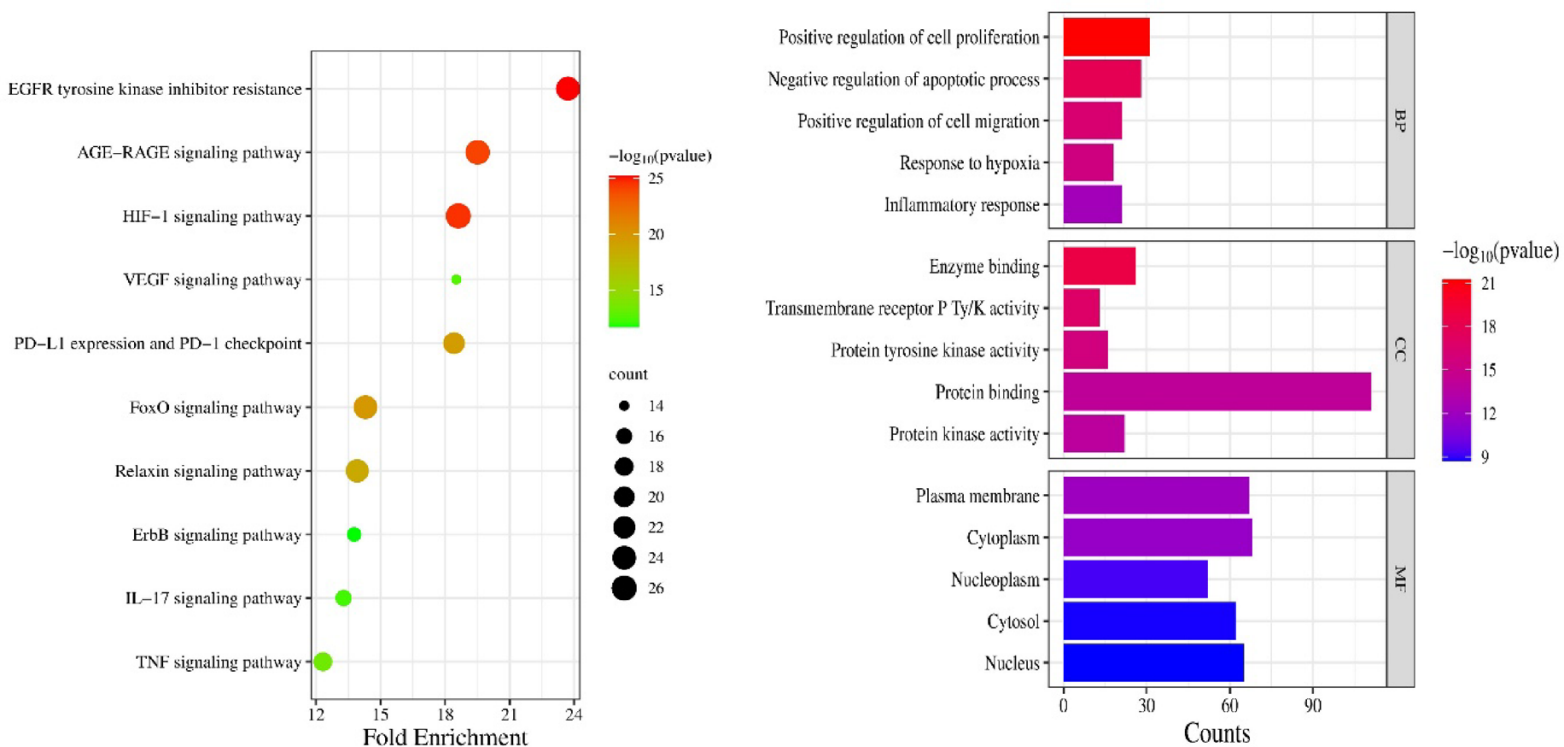
Gene ontology functional annotations (BP-Biological process, CC-Cellular components, MF-Molecular function) and KEGG pathway enrichment.

**Fig. 4:**
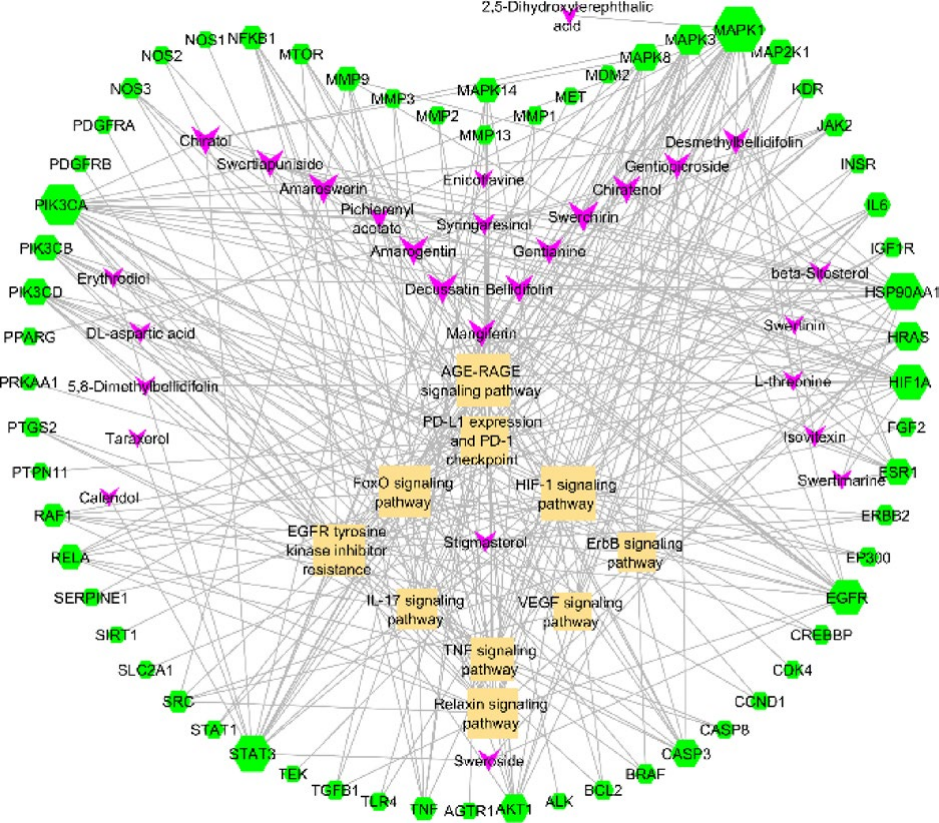
C-T-P network built using 10 KEGG pathways with the genes involved in that and compounds predicted targets.

KEGG pathway analysis showed that targets were enriched in 171 pathways overall (Supplementary file S2-4). With the criteria of P-value < 0.05, enrichment fold, and core targets involvement, the top 10 targets enriched in pulmonary fibrosis were considered, as shown in Fig. 3^31^. The SC regulates EGFR tyrosine kinase inhibitor resistance, PD-L1 expression, and the PD-1 checkpoint, as well as TNF, EGFR, VEGF, HIF-1, FoxO, relaxin, ErbB, and IL-17 signaling pathways, primarily in IPF. As shown in the top 5 BP processes, the components of SC may promote fibroblast cell apoptosis and suppress the proliferation and migration of these cells. Moreover, it lessens inflammatory responses and hypoxia. The top 5 CC and MF composites out of 78 and 131 were represented in Fig. 3. The C-T-P network, as represented in Fig. 4, shows the interactions between the potential components with the targets involved in the pathways. The polypharmacological effect was observed through multiple compounds interacting with multiple targets via major pulmonary fibrosis signaling pathways. It comprised 28 compounds, 61 targets, and 10 major signalings. Overall, decussatin, bellidifolin, mangiferin, swerchirin, amarogentin, gentiopicroside, and amaroswerin exhibited good degrees of interaction, characterized by strong binding potentials. The highly connected nodes, such as EGFR, STAT3, PIK3CA, MAPK, HIF1A, AKT1, and TNF, suggest their role in anti-fibrotic activity. Pathways that interact with a greater number of nodes and exhibit good enrichment scores represent all 10 signaling pathways, which play a crucial role in the polypharmacological effect of SC. The compounds of SC show good interactions with all pathways, and TNF signaling was prioritized due to its involvement in initiating the inflammatory and fibrotic foci cascade. TNF-α, along with its downstream signaling, collectively promotes fibroblast migration, proliferation, and myofibroblast differentiation and EMT, unlike other signaling pathways.

### Molecular docking

The nine targets IL6 (PDB: 1ALU), TNF (2AZ5), MMP9 (4XCT), EGFR (3POZ), STAT3 (6NJS), FGF2 (1BFB), MAPK3 (3FHR), AKT1 (4GV1), and SRC (1O41) were docked against all the compounds of SC with respect to their particular binding pockets. The binding site of the 1ALU was identified through SiteMap and evaluated as a druggable pocket at site 1 (Table 2) with a site score of 0.983, a Dscore of 0.985, and a volume of 486.03 Å (Table 3). Received an unsatisfactory docking score (more than − 5.0 kcal/mol, except for a few components) and contacts between 1O41, 1BFB, and 6NJS PDB ID and the SC components. As per the molecular docking analysis, SC components inhibit many targets involved in the TNF-α signaling pathway.

**Table 2:** SiteMap results with the suitable site for molecular docking of *Swertia chirayita*.

PDB ID	Site	Site score	Druggability score	Volume Ǻ^3^	Residues
1ALU-IL-6	Site 1	0.983	0.985	486.03	Chain A: 54,55,56,57,58,59,60,61,62,63,64,65,66,67,68,70,71,74,86,89,90,92,93,95,96,97,139,143,144,147,150,154,158,161,162,165,168,169,172,173,176,302,315,336,381

**Fig. 5:**
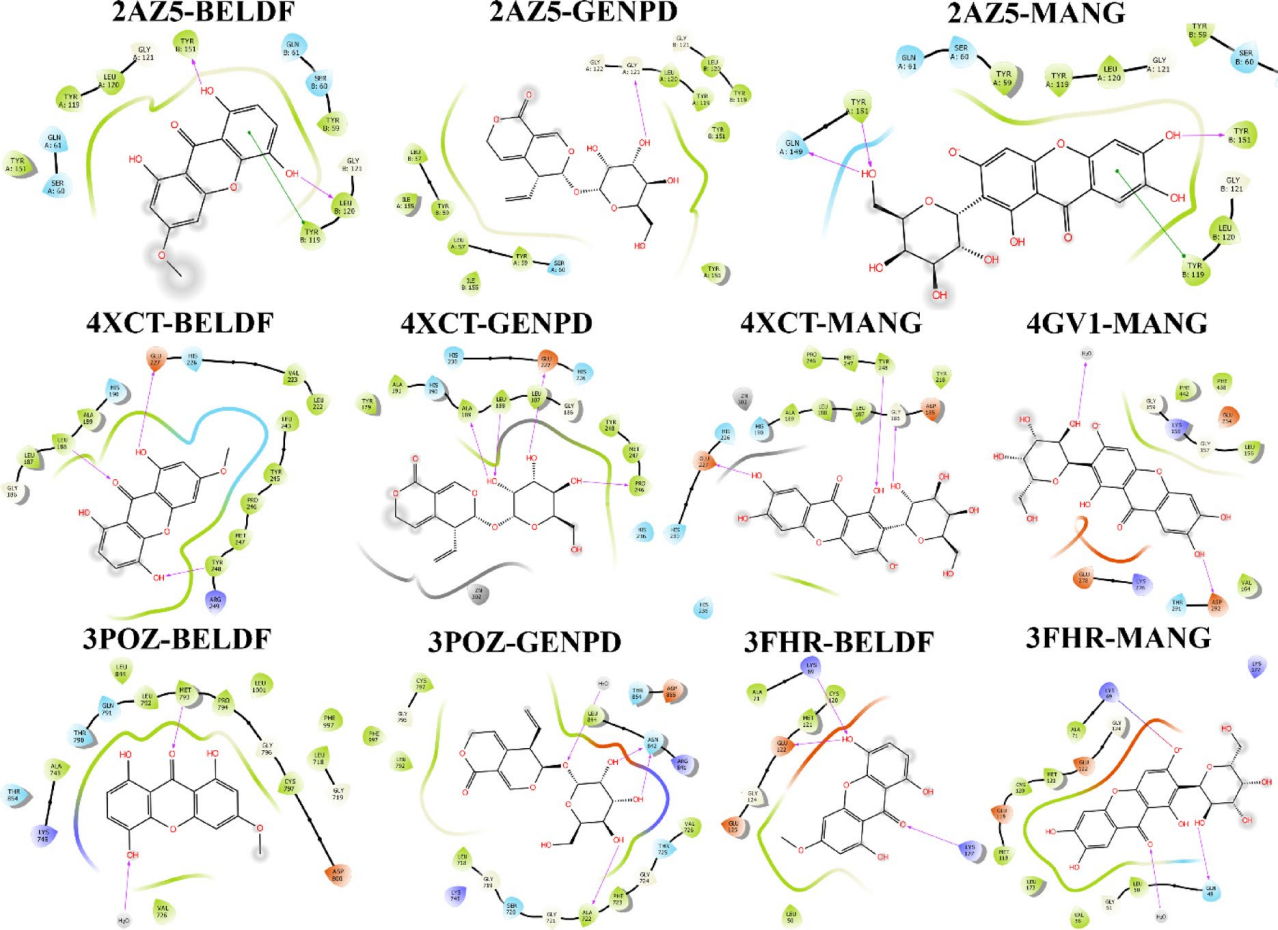
2D interaction diagram for all complexes obtained via molecular docking that was taken into account for MD simulations.

As represented in Table 3, good binding affinity was identified for the components like MANG, BELDF, GENPD, Demethylbellidifolin (DMBEDF), Sweroside (SWIDE), 2,5-Dihydroxyterephthalic acid (DTPA), Swerchirin (SWCIN), Chiratol (CHIRT), and Swertiamarin (SWRMN). However, the compounds MANG, BELDF, and GENPD were chosen for additional MD simulation based on the H-bond interactions and score, docking score, lipophilic interactions, penalty, and electrostatic rewards consideration at the binding site as detailed in the 2D interaction Fig. 5 and supplementary file S4, 1–6. Based on the binding affinity and interactions shown, SWCIN, CHIRT, SWIDE, and DTPA also affect TNF, EGFR, MMP9, and MAPK3 proteins.

**Table 3:** Docking score of *Swertia chirayita* core targets and potential components.

Components	MMP9 (4XCT)	EGFR (3POZ)	TNF (2AZ5)	IL-6 (1ALU)	MAPK3 (3FHR)	AKT1 (4GV1)
Mangiferin	−5.63	−7.35	−7.02	−6.13	−7.64	−4.84
Gentiopicroside	−6.65	−7.26	−6.45	−3.31	−4.47	−3.25
Bellidifolin	−6.43	−7.02	−6.26	−4.97	−5.33	−4.68
Demethylbellidifolin	−5.23	−7.25	−5.82	−4.68	−5.60	−4.24
2,5-Dihydroxyterephthalic acid	−5.94	−6.82	−4.05	−3.89	−4.82	−4.74
Swerchirin	−5.87	−6.76	−5.74	−4.52	−7.33	−4.24
Chiratol	−5.69	−6.54	−5.75	−5.35	−6.56	−3.87
Sweroside	−5.40	−5.44	−7.22	−3.79	−4.50	–
swertiamarin	−5.80	−6.88	−5.55	−5.53	−3.47	–

Table 4 shows H-bond (bold) and hydrophobic interactions of MANG with MMP9, AKT1, MAPK3, and TNF-α amino acid residues at the inhibitory binding site^52–55^.

**Table 4:** Mangiferin (MANG) interaction pattern with the represented proteins.

Proteins	No. of H-bonds	Amino acid residue contact
MMP9	3	**GLU227 (2.19)**,** GLY186 (2.14)**,** TYR248 (2.51)**, HIS190, HIS236, MET247, LEU188, ALA189, PRO246, HIS 230
AKT1	1	**ASP292 (2.17)**, THR291, LYS158
MAPK3	2	**GLN48 (1.89)**,** LYS69 (3.05)**, LEU50, CYS120, MET121, GLU122
TNF	3	**TYR151 (Chain A:2.07)**, **GLN148 (1.86)**,** TYR119 (5.06)**, LEU57, GLY121, TYR151, TYR119

Table 5 shows H-bond (bold) and hydrophobic interactions of GENPD with MMP9, EGFR, and TNF-α amino acid residues at the inhibitory binding site^52,56,57^.

**Table 5:** Gentiopicroside (GENPD) interaction pattern with the represented proteins.

Proteins	No. of H-bonds	Amino acid residue contact
MMP9	4	**PRO246 (1.67)**,** GLU227 (2.78)**,** LEU188 (1.88)**,** ALA189 (1.97)**, HIS236, HIS230, GLY186, HIS190
EGFR	3	**ASN842 (1.87 and 1.81)**,** ALA722 (2.62)**, ASP800, SER720, LYS745, THR854, VAL726
TNF	1	**GLY121 (2.69)**, TYR119, SER60, TYR151, LEU57, TYR59

Table 6 shows H-bond (bold) and hydrophobic interactions of BELDF with MMP9, EGFR, MAPK3, and TNF-α amino acid residues at the inhibitory binding site^52,56,57^.

**Table 6:** Bellidifolin (BELDF) interaction pattern with the represented proteins.

Proteins	No. of H-bonds	Amino acid residue contact
MMP9	3	**GLU227 (1.90)**,** LEU188 (2.40)**,** TYR248 (2.15)**, PRO246, HIS236, PRO180, HIS190, MET247, ALA189, HIS 230
EGFR	1	**MET793 (2.64)**, THR854, LYS745, ASP800, GLN791
MAPK3	3	**LYS177 (2.36)**,** GLU122 (1.84)**,** LYS69 (1.88)**, GLN48
TNF	2	**TYR151 (1.75)**,** LEU120 (1.90)**,** TYR119 (5.21)**, TYR59, SER60, GLY121,

### Induced fit docking

All of the poses were examined for interactions and scores using the interaction matrix shown in Table 7 and the supplementary file S3-3. The first pose from MANG, BELDF, and GENPD was chosen using the MMP9-ligand complex for MD simulation. Poses 2 and 4 for EGFR were chosen from GENPD and BELDF, respectively. Pose 1 has been provided by BELDF and GENPD, while MANG pose five was chosen for MD simulations with good interactions of TNF. To run MD simulations using MAPK3, poses 3 and 1 were acquired for MANG and BELDF, respectively. For the MD simulations, pose four from the MANG with AKT1 was taken into consideration.

**Table 7:** Induced fit docking (IFD) score and pose of MANG (Mangiferin), BELDF (Bellidifolin), and GENPD (Gentiopicroside) and targets considered for the MD simulation.

Components	4XCT		3POZ		2AZ5		3FHR		4GV1	
	IFD score	Pose	IFD score	Pose	IFD score	Pose	IFD score	Pose	IFD score	Pose
MANG	−370.62	1	-	-	−609.63	5	−610.14	3	−742.56	4
BELDF	−364.69	1	−683.74	4	−611.55	1	−611.29	1	-	-
GENPD	−355.45	1	−678.31	2	−603.24	1	-	-	-	-

### Molecular dynamics

**Fig. 6:**
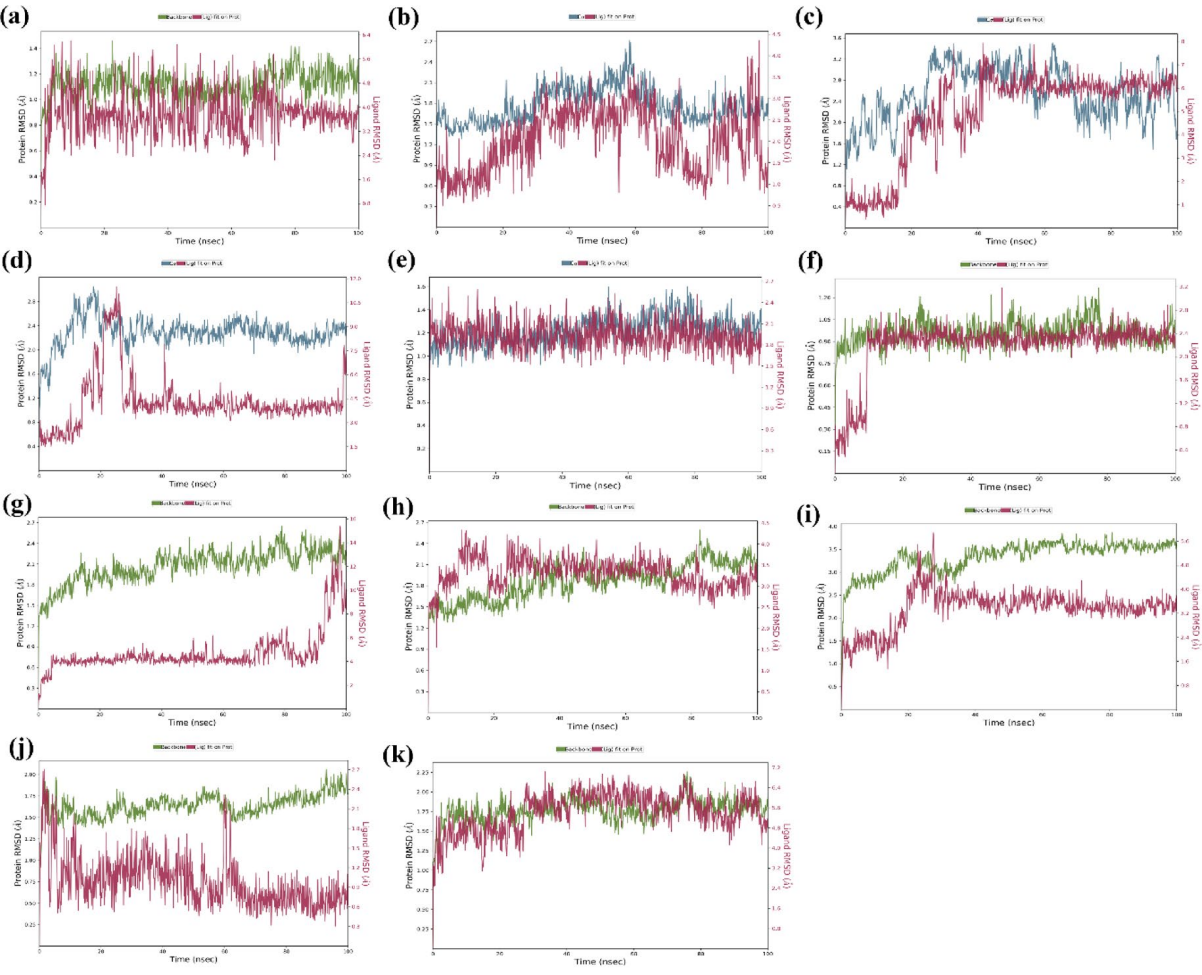
Molecular dynamic simulations RMSD results represented (**a**) MMP9-BELDF, (**b**) TNF-BELDF, (**c**) MAPK3-BELDF, (**d**) EGFR-BELDF, (**e**) MMP9-MANG, (**f**) MMP9-GENPD, (**g**) TNF-MANG, (**h**) TNF-GENPD, (**i**) EGFR-GENPD, (**j**) AKT1-MANG, (**k**) MAPK3-MANG complex.

**Fig. 7:**
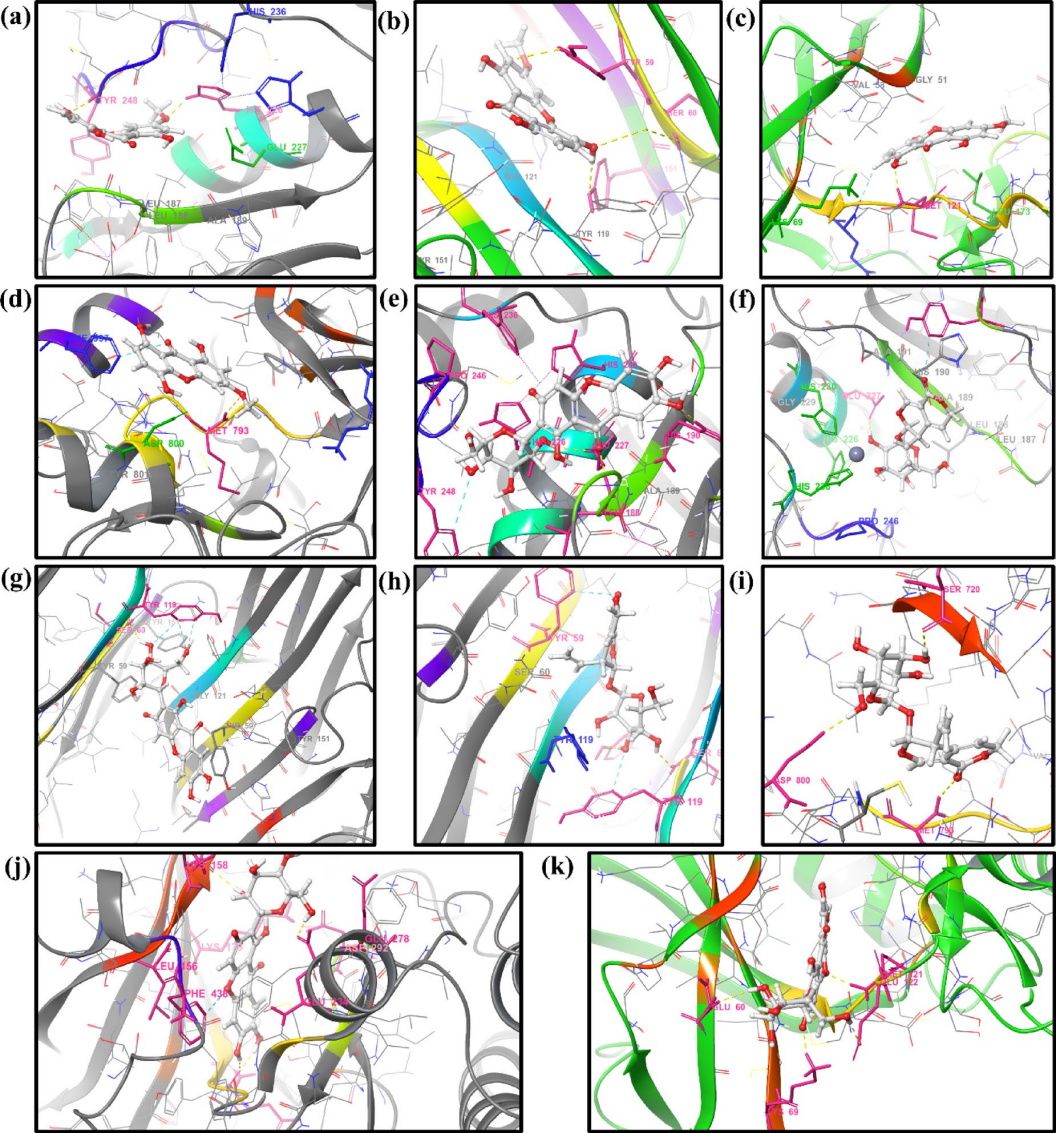
3D ligand-protein interactions of molecular dynamic simulations of (**a**) MMP9-BELDF, (**b**) TNF-BELDF, (**c**) MAPK3-BELDF, (**d**) EGFR-BELDF, (**e**) MMP9-MANG, (**f**) MMP9-GENPD, (**g**) TNF-MANG, (**h**) TNF-GENPD, (**i**) EGFR-GENPD, (**j**) AKT1-MANG, (**k**) MAPK3-MANG complex.

*MMP9-BELDF* The RMSD was observed to be less than 3.5 Å with stable interactions with the protein (Fig. 6a). The TYR248 H-bond interaction occupancy was observed to be at 94% as seen in Fig. 7a. Moderate H-bond and hydrophobic interaction were seen at LEU188. The RMSF of the protein is very stable; there is no flexibility of residues. The ligand contacts with the protein residues made stable interactions with low RMSF due to the presence of strong H-bond interactions at TYR248 and other hydrophobic interactions. The solvent exposure was found to be less than 60 Å^2^, and rGyr shows 3.77 ± 0.05 Å. All graphs related to RMSF, contact histogram, contact timeline, and 2D ligand-protein interactions of MD simulations were depicted in the supplementary file S3-5. Graphs related to solvent exposure and rGyr were represented in the supplementary file S3-4.

*TNF-BELDF* Throughout the 100-ns simulation, the protein-ligand interaction remained within 3.5 Å (Fig. 6b), having begun with the stabilized RMSD value. Along with the H-bond with TYR151 and other hydrophobic bonds shown in Fig. 7b, there is a good aromatic interaction with TYR59. The RMSF graph demonstrates that there is little fluctuation in the protein flexible loops at 18–23, 95–105, 145–150, and 240–250 residues with 3.5 Å RMSF. The beta-strands tend to be intact and less flexible, and ligand interaction facilitates the compactness of the protein through essential interactions, as shown in S3-5. The solvent exposure of the ligand was less with 40 Å^2^, and rGyr shows 3.74 ± 0.05 Å.

*MAPK3-BELDF* The protein and ligand formed a stable complex, as shown in Fig. 6c, with a little fluctuating from 17 ns for another 20 ns but remaining inside the binding pocket and conserving less than 3 Å RMSD after that. The fluctuation was seen due to the interaction with LYS73 and THR186 being lost, but the ligand preserved the H-bond connection with the MET121 (Fig. 7c) residue and the hydrophobic contact with LEU173 during the little fluctuation. The water bridge allowed for the observation of several hydrophobic interactions, as seen in S3-5. The protein residues are not very variable, with binding pockets even exhibiting less flexibility when interacting with ligands, according to the RMSF graph. Less than 0.14 Å (3.73 ± 0.07) was observed for rGyr, and the SASA was observed within 100 Å^2^. BELDF creates a negative impact on intramolecular hydrogen bonds.

*EGFR-BELDF* The shift in the hydrophobic bond interaction from ALA743 to LYS745 inside the binding pocket of the ligand and protein was responsible for the 14ns fluctuation (Fig. 6d) that was seen between 17.6ns and 31ns, along with the interactions observed with GLU1005 and LEU1001. A flexible loop region was seen in the protein at residues 160–180 and 215–235; however, it was smaller at 3 Å and not in the binding pocket. The ligand-binding pocket interactions revealed less flexible strands and helices. Among the other significant hydrophobic contacts GLN791, ASP800, LYS745, and LEU718, as illustrated in Fig. 7d, a significant MET793 residue interaction as an H-bond with ligand provides a strong inhibition supposition. The solvent exposure was higher during the 10 ns fluctuation and stable over the period of simulation within 100 Å^2^. The rGyr was shown at 3.76 ± 0.08 Å.

*MMP9-MANG* The ligand is stabilized at the binding pocket of the protein. A 99% H-bond occupancy was observed for GLU227 and LEU188 (Fig. 7e) with the ligand, and a moderate H-bond was seen for ALA189. Pi-Cation interactions between Zn coordination and ligand provide an effective binding effect for MMP9 through HIS226, HIS230, and HIS236 residues. The ligand formed a stable interaction with the protein and gives 1.3 Å RMSD (Fig. 6e). There are no flexible loops seen in the RMSF of the protein. Solvent exposure was limited to 50 Å^2^ and 4.50 ± 0.06 Å rGyr.

*MMP9-GENPD* Figure 7f showed residues GLU227 form an H-bond directly with the ligand at 99% occupancy, and ALA189 and LEU188 formed bridged H-bond interaction with water at 65% occupancy each. The contact histogram (S3-5) shows 100% Pi-Cation interactions of HIS226, HIS230, and HIS236 via Zn coordination, which is required for a compound’s potency to inhibit MMP9. During an initial 10 ns, fluctuation was seen in response to the repositioning of loops surrounding the active site for better binding with the ligand and maintained 1.2 Å RMSD (Fig. 6f) throughout the simulation period. MANG developed fluctuating contacts with MMP9’s GLU227 residue from 5 ns to 10 ns before stabilizing in the binding pocket, then migrated away from the GLY186 and TYR179 residues after 4 ns to build a stable association with ALA189. The RMSF data in the figure depicts the significantly less flexibility of a protein with the GENPD ligand. rGyr shows 3.73 ± 0.07 Å, and solvent exposure was reduced to 70 Å^2^.

*TNF-MANG* As demonstrated in S3-5, the flexible binding site residues 145–153, solvent exposure, and interactions with other residues like ALA156 caused the 3 Å RMSD to fluctuate in the final 8 ns of the simulation (Fig. 6g). At 91 ns, the hydrogen bond interactions with TYR59 and TYR151 were lost and exposed to the solvent with 120 Å^2^, causing them to shift slightly away from the binding interaction site. The displayed rGyr was 4.55 ± 0.07 Å. The complex retained the bridged interaction with SER60 and TYR151 at 58% and 46% occupancy (Fig. 7g). The ligand and protein residue TYR59 was observed to form a moderate π-π interaction.

*TNF-GENPD* GENPD consistently demonstrated binding stability within the TNF binding pocket. TYR151 and SER60 (Fig. 7h) allowed for the retention of robust H-bond interactions at 91% and 79%, respectively, throughout the simulation. The RMSD was maintained below 2 Å variation (Fig. 6h) with less than 80 Å^2^ solvent exposure and 3.90 ± 0.15 Å rGyr. Other than the binding site, many flexible loops with 2–3.5 Å RMSF fluctuations were seen.

*EGFR- GENPD* Before stabilizing to less than 2 Å, the RMSD showed a minor change at 20 ns for the next 10 ns (Fig. 6i) due to the loss of interaction with VAL726, ASP800, and MET793’s interaction with the ligand that was partially missed. Following stabilization, rGyr varied to 3.6 ± 0.15 Å, and solvent interaction was decreased to maintain at 80 Å^2^ SASA. Compared to all stable and helical strands, the flexible loop regions at residues 160–180 and 280–300 showed a minor increase in RMSF of 3.6–4.2 Å. The ligand has shown a strong 94% continuous H-bond interaction with the MET793 required for the inhibition. ASP800 also showed strong interaction with the ligand (Fig. 7i), and a moderate interaction was observed by SER720 and CYS797 (through the water bridge).

*AKT1- MANG* MANG inhibits AKT1 as shown by Fig. 7j, two direct H-bond interactions with the ALA230 and GLU228 (60% and 81% occupancy), the rest of the other interactions were bridged H-bonds from LEU156, LYS158, ASP292, LYS179, ASP439, GLU234, and GLU278. RMSD plot Fig. 6j shows that it is within 2 Å to maintain the stabilized complex interactions during the simulation. There were no flexible loops, strands, or helices in the protein RMSF. Solvent exposure was seen in less than 50 Å^2^ SASA after the initial stabilization, and rGyr was seen at 4.50 ± 0.06 Å.

*MAPK3-MANG* Figure 7k showed GLU60, LYS69, MET121, and GLU122 residues of MAPK3 formed good H-bond interactions with the ligand, along with the two-water bridged interaction with LEU52 and CYS70 residues. The ligand fluctuated more than the other protein interactions shown by MANG at 3 Å RMSD (Fig. 6k). A flexible loop area was indicated by the high RMSF values of residues 160–170, whereas a stable helical and strands were indicated by the low RMSF values. Since there was a positive correlation between the ligand and residues with H-bonds, there was a reduced RMSF value at the binding pocket, which suggested limited flexibility. The ligand was exposed to solvent during the initial phase and fitted within the binding pocket with 80 Å^2^ SASA. The rGyr was observed at 4.48 ± 0.08 Å.

### Principal component analysis

**Fig. 8:**
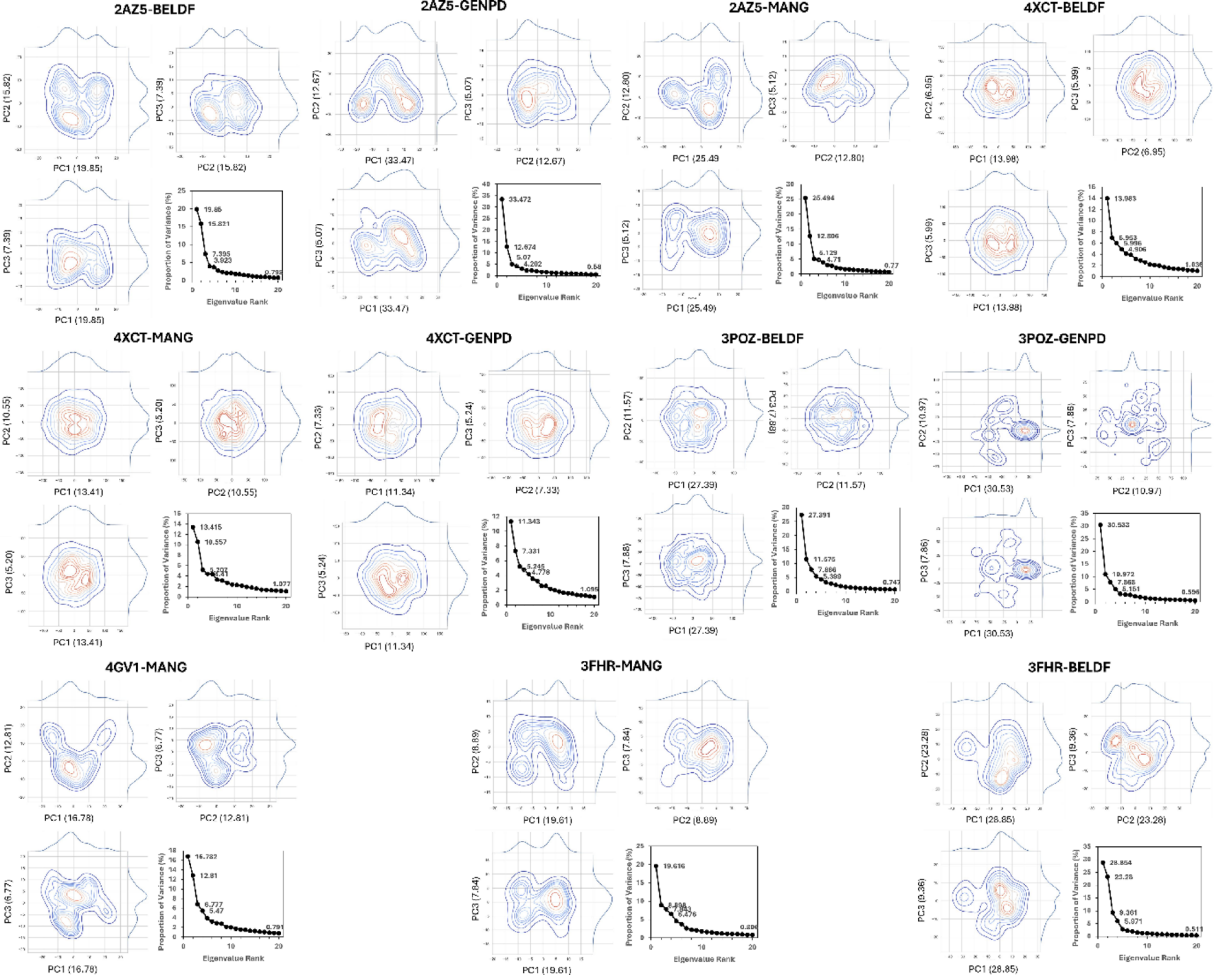
PCA analysis for all the MD protein-ligand complexes with 3 PC variations, and the top 20 PC variances were represented in the plot graph.

*PCA:* The three eigenvalues ranked top in PCA analysis show that the majority of collective motions account for 19.85% (PC1), 15.82% (PC2), and 7.39% (PC3) of the total variance in 2AZ5-BELDF. Clustering patterns of PC1, PC2, and PC3 of 2AZ5-GENPD (33.47, 12.67, and 5.07), 2AZ5-MANG (25.49, 12.80, and 5.12), 3FHR-BELDF (28.85, 23.28, and 9.36), 3POZ-BELDF (27.39, 11.57, and 7.88), 3POZ-GENPD (30.53, 10.97, 7.86), 4XCT-BELDF (13.98, 6.95, and 5.99), 4XCT-GENPD (11.34, 7.33, and 5.24), 4XCT-MANG (13.41, 10.55, and 5.20), 4GV1-MANG (16.78, 12.81, and 6.77), and 3FHR-MANG (19.61, 8.89, and 7.84) were represented in Fig. 8, suggesting the existence of stable conformational states with dynamic transitions between them. The closely packed contours of all the protein-ligand complexes represent the most stable conformation, except the 3POZ-GENPD complex, which shows spread contours as indicating conformational flexibility of non-ligand binding amino acid residues 160–175 (Fig. 14). The stable system conformations were shown by the top variances dispersion in the cluster plot analysis.

**Fig. 9:**
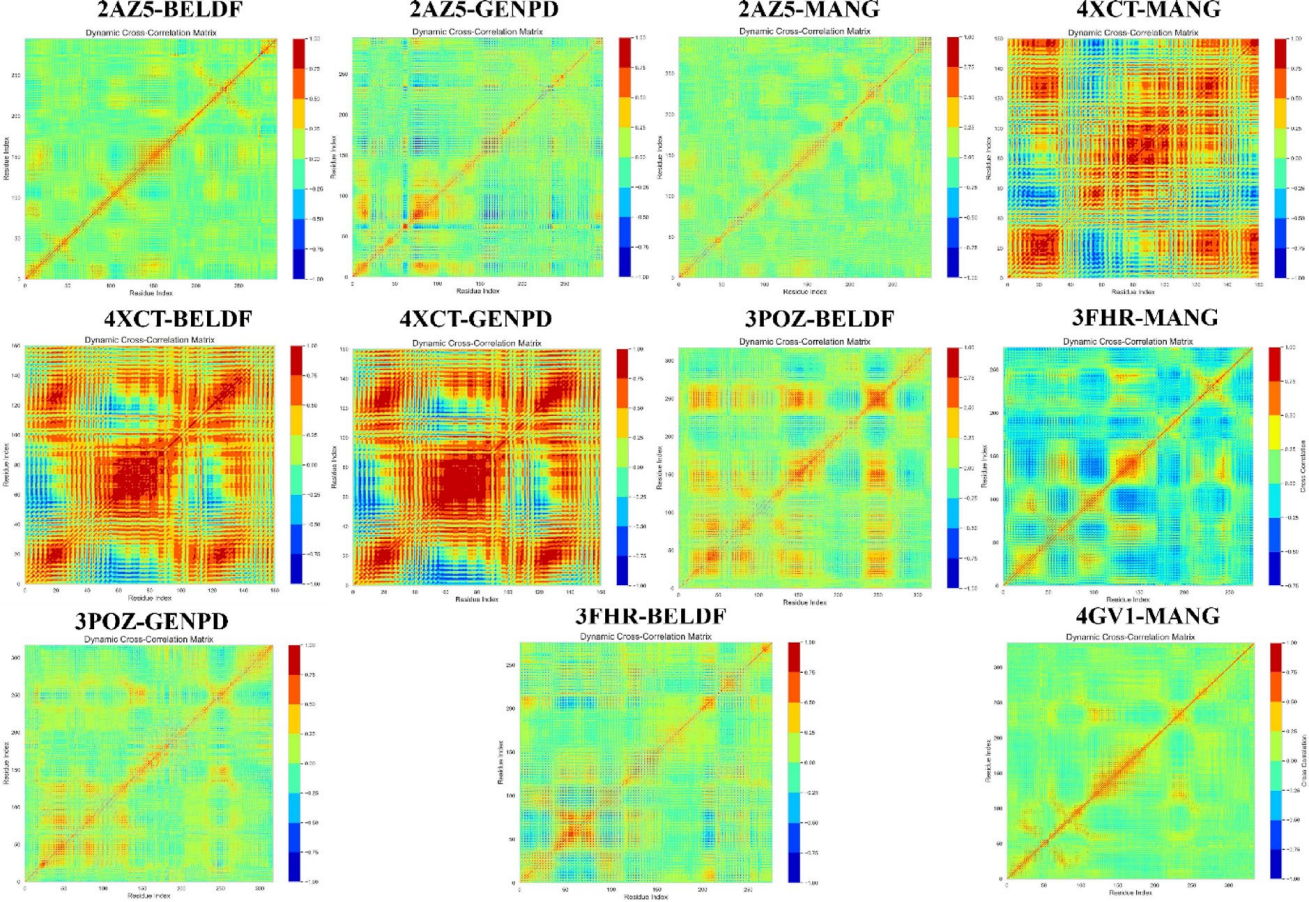
DCCM of Map analysis of the trajectory of protein-ligand complexes of SC. The color scale shows the strength of the correlation; red (+ 1) indicates a positive correlation, while blue (− 1) indicates a negative correlation.

**Fig. 10:**
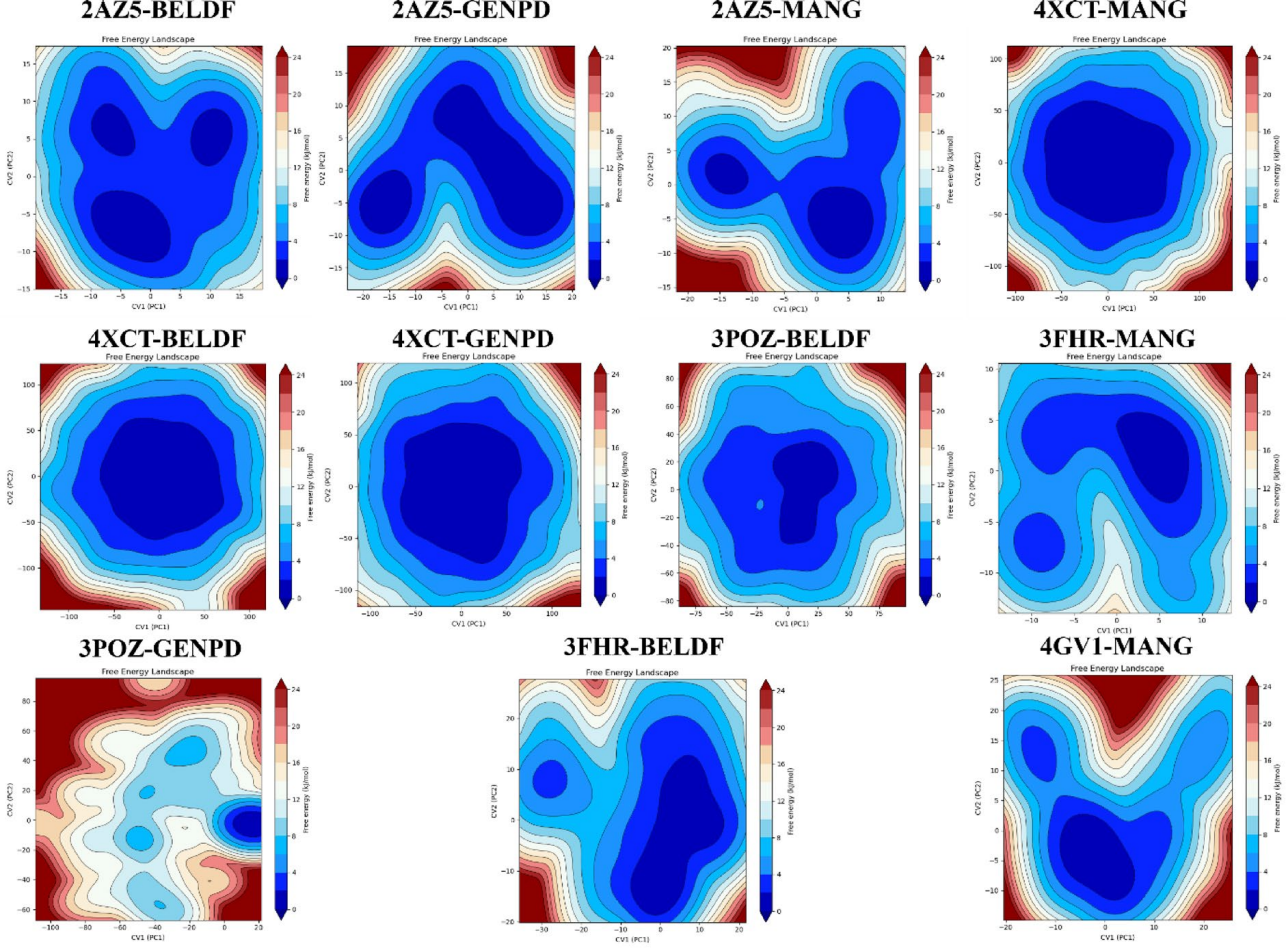
Heatmap of the Free energy landscape of the protein-ligand complex. Blue color represents a stable conformation state and high energy barriers with a light color towards red.

*Domain cross-correlation map (DCCM) analysis*: Additionally, the movement correlation of the protein complex system was examined using DCCM analysis. The diagonal deep red indicates a stable correlation of each residue in the protein-ligand complex, as shown in Fig. 9. The collective motion of residues within the protein, as red clusters and yellowish green off the diagonal, strongly correlated. As shown in the dynamic simulations, certain residues of the protein showed flexibility by moving in the opposite direction, represented with blue clusters. All the complexes exerted superior stability as they are highly self-correlated with red diagonal motion. In contrast to other complexes, 3FHR-MANG has shown few blue clusters. However, the red diagonal indicates a positive correlation; the protein’s RMSF likewise showed no flexible mobility.*Free energy landscape analysis*: The stability of the protein-ligand complex structure and conformational distribution have been examined using free energy landscape analysis shown in Fig. 10, based on the simulated trajectory. The stable conformation state of 2AZ5-BELDF was observed in the dark blue and blue areas (free energy minima) in the PC1 and PC2 free energy landscape, with lower free energy states conserved between the complex figures. The conformational state transition was observed to be less probable, with higher energy barriers (light color regions closer to red) seen around the minima (all complexes). The multiple low-energy conformational states were seen with deep basins in the blue area, indicating a more binding pose and multiple stable conformations, as well as their transitions within the basins. Comparable to 2AZ5-BELDF, but with a very little higher free energy area, 2AZ5-GENPD has multiple stable conformation poses and less conformational transitions. 2AZ5-MANG has shown stable conformation, multiple binding poses, and conformational transition within basins, but comparatively less than 2AZ5-BELDF with a higher free energy area. 3FHR-BELDF, 3FHR-MANG, and 4GV1-MANG have shown a single low-energy conformational state with one deep basin. Contour lines are concentrated and elongated around the blue region, which is less complex and shows fewer transitions between conformations due to one large stable conformation and the ligand staying here for a longer duration. 3POZ-BELDF, with a large individual conformational state, tends to remain in this stable state for a longer duration. The conformational state transition was observed to be less probable with the higher energy barriers. 3POZ-GENPD shows higher free energy states compared to all the complexes due to the flexible loop residues observed in the MD simulation. Meanwhile, low-energy states were also observed in 4XCT-BELDF, 4XCT-MANG, and 4XCT-GENPD with a single stable conformational state, which shows stable interactions here for a long duration without transition. They have a large basin with stable conformation for a long duration.

**Fig. 11:**
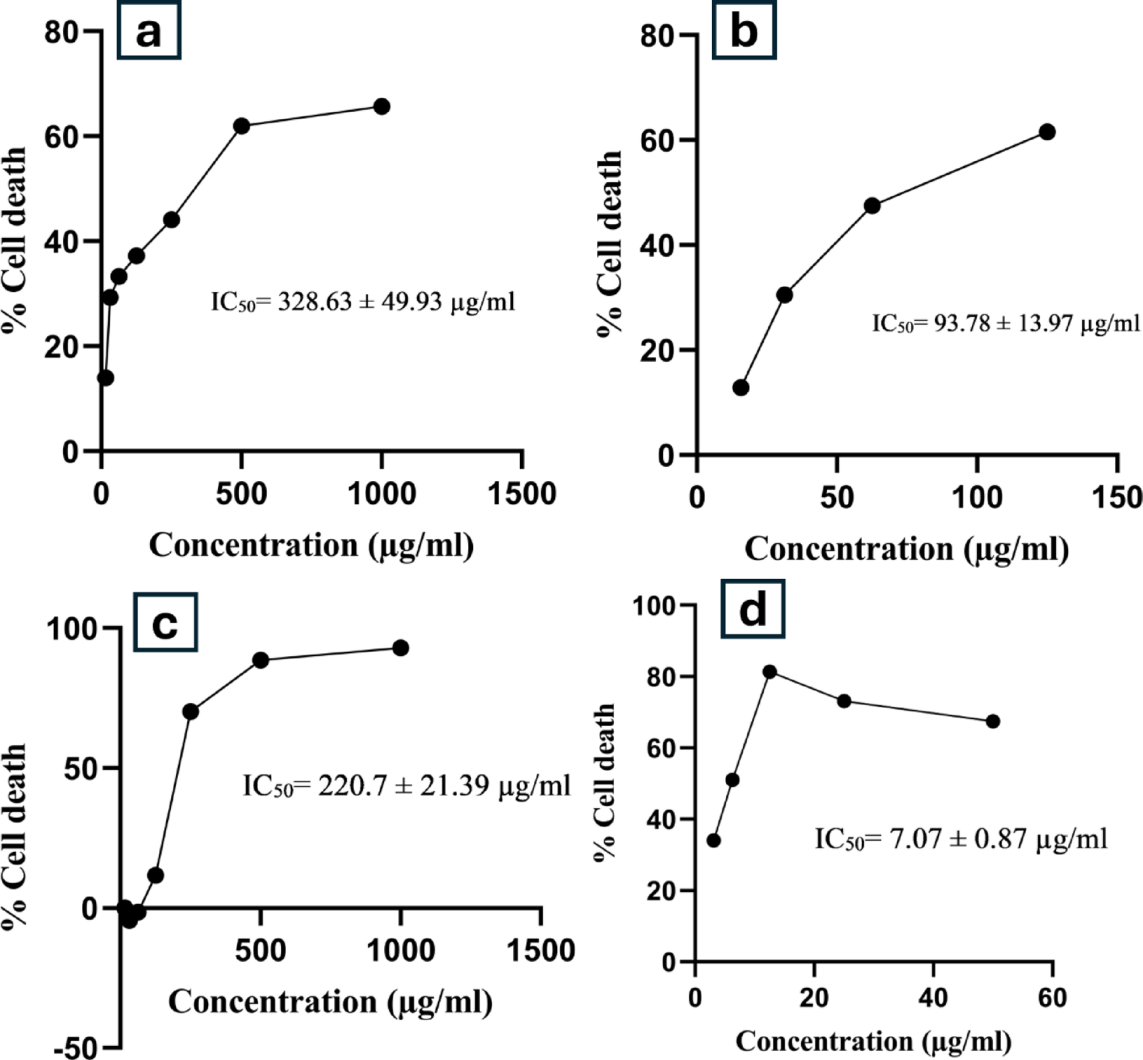
Concentration and percentage cell death of (**a**) *Swertia chirayita* and (**b**) nintedanib in A549 cells; (**c**) *Swertia chirayita* and (**d**) nintedanib in NIH3T3 cells.

### Cytotoxicity assay on NIH3T3 and A549

After a 24-hour incubation period with A549 cells, the IC50 values for SC and nintedanib were 328.63 ± 49.93 µg/ml and 93.78 ± 13.97 µg/ml, respectively. SC and nintedanib, on the other hand, demonstrated IC50 values of 220.7 ± 21.39 µg/ml and 7.07 ± 0.87 µg/ml in NIH3T3, respectively. Figure 11 depicts the concentration vs. percentage of cell death in A549 and NIH3T3 cells.

**Fig. 12:**
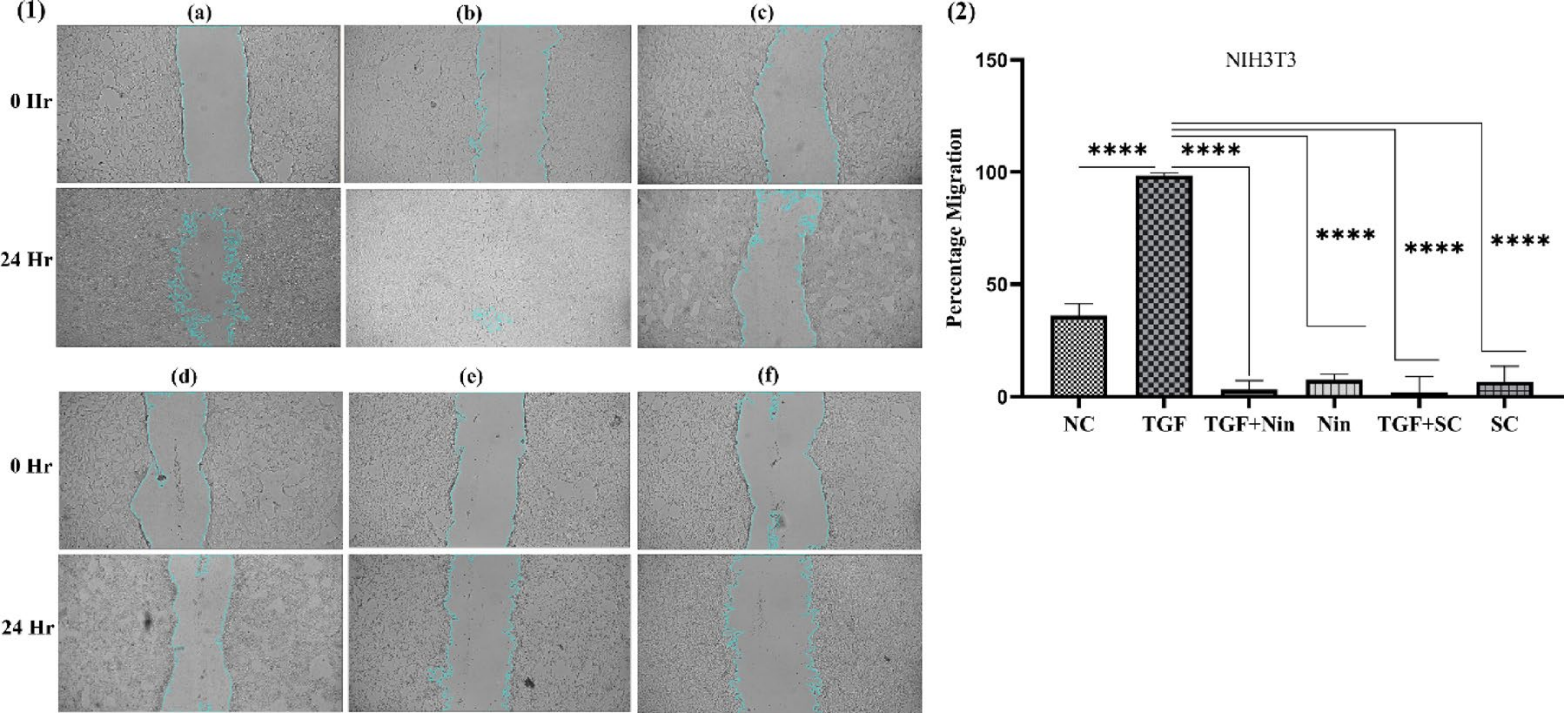
NIH 3T3 cell scratch wound assay, (1) Visuals of cell migration from 0 h to 24 h with or without TGFβ1 (**a**) Normal, (**b**) TGFβ1 stimulated, (**c**) TGFβ1 and nintedanib treated, (**d**) Nintedanib treated, (**e**) TGFβ1 and *Swertia chirayita* treated, and (**f**) *Swertia chirayita* treated. (2) Activity of fibroblast proliferation and differentiation with TGFβ1 stimulation and its inhibition by *Swertia chirayita* and nintedanib treatment. The values are represented in mean ± SEM, ****p < 0.0001, ***p < 0.0005, **p < 0.005, *p < 0.05 vs. TGF (*n* = 3). NC- normal cell, TGF-TGFβ1, Nin- nintedanib, and SC- *Swertia chirayita*.

### TGFβ1-induced fibroblast proliferation and differentiation

The percentage of cell migration and differentiation upon TGFβ1 stimulation in NIH3T3 was assessed using a scratch wound healing assay, as shown in Fig. 12 (1), to examine the therapeutic effects of SC (110 µg/ml) and nintedanib (4 µg/ml). TGFβ1 stimulation in NIH3T3 cells demonstrated a very significant 98.46 ± 2.41% cell migration in comparison to 35.99 ± 2.41% NIH3T3 cells without TGFβ1, which shows the fibroblast proliferation and differentiation. The study showed that the migration of 3T3 cells was significantly inhibited in SC (6.66 ± 3.22%) and nintedanib (7.49 ± 1.15%) treatment for 24 h. Whereas treatment with SC (1.86 ± 3.34%) and nintedanib (3.15 ± 1.90%) subsequently stimulated with TGFβ1 showed significant inhibition of fibroblast migration and its differentiation (Fig. 12 (2)).

**Fig. 13:**
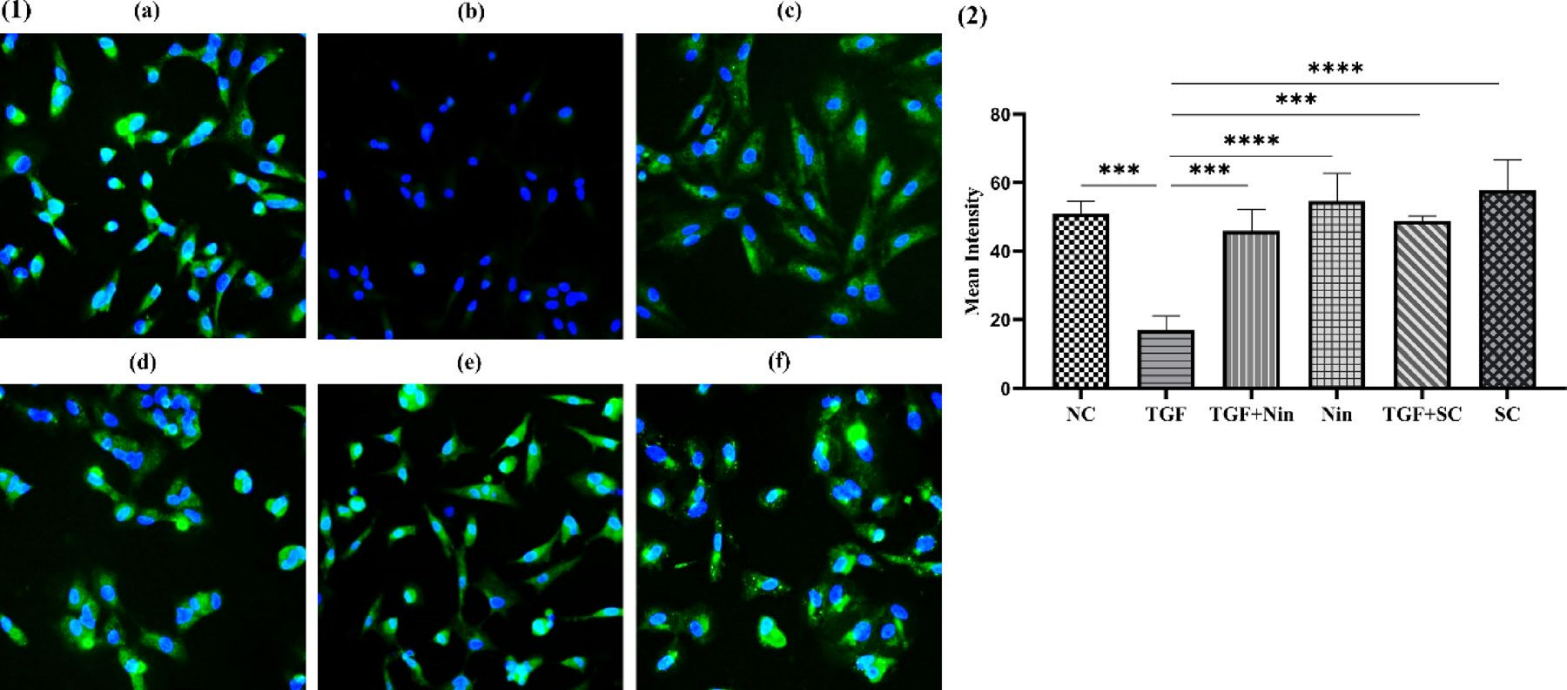
Immunofluorescence assay of (1) E-cadherin (green) intensity in A549 epithelial cells (blue nucleus) with or without TGFβ1. Whereas (**a**) Normal, (**b**) TGFβ1 stimulated, (**c**) TGFβ1 and nintedanib treated, (**d**) Nintedanib treated, (**e**) TGFβ1 and *Swertia chirayita* treated, and (**f**) *Swertia chirayita* treated. (2) Mean intensity of E-cadherin related to the EMT with TGFβ1 stimulation and its inhibition by *Swertia chirayita* and nintedanib. The values are represented in mean ± SEM, ****p < 0.0001, ***p < 0.0005, **p < 0.005, *p < 0.05 vs. TGF (*n* = 3). NC- normal cell, TGF-TGFβ1, Nin- nintedanib, and SC- *Swertia chirayita*.

### SC inhibits TGFβ1-induced EMT in A549 cells (Immunofluorescence assay)

As illustrated in Fig. 13 (1), A549 cells stimulated with TGFβ1 (4 ng/ml) for 24 h exhibited a significant downregulation of the epithelial marker E-cadherin (green fluorescence: 20.6 ± 2.37 mean intensity) in comparison to normal A549 cells (50.95 ± 2.11). TGFβ1-containing A549 cells were then treated for 24 h with SC (180 µg/ml) and nintedanib (30 µg/ml). By preserving the levels of the epithelial marker E-cadherin in TGFβ1-stimulated A549 cells, SC (48.82 ± 3.64) and nintedanib (45.9 ± 0.82) effectively inhibited mesenchymal transition through TGFβ1 signaling, as seen by the microscopic visualisations in Fig. 13 (1) and (2). Conversely, both nintedanib (54.57 ± 5.17) and SC (57.74 ± 4.71) treatments substantially raised E-cadherin levels in A549 cells without TGFβ1.

**Fig. 14:**
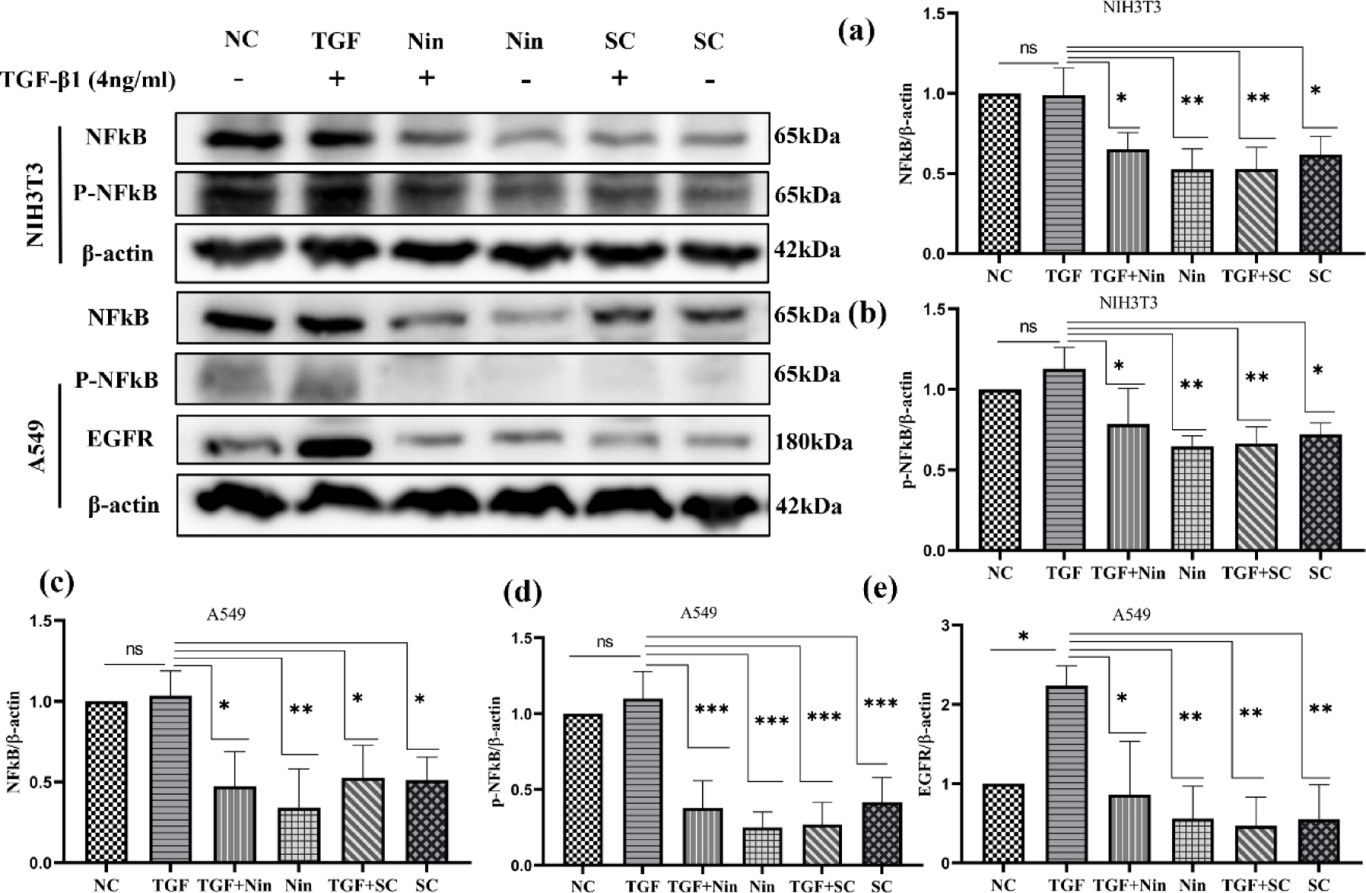
Effect of *Swertia chirayita* and nintedanib by inhibiting TNF-α signaling through canonical NF-κB and its phosphorylation in A549 mesenchymal cells and NIH3T3 fibroblast cells. *Swertia chirayita* and nintedanib reduced (**a**) and (**c**) NF-κB and (**b**) and (**d**) p-NF-κB levels in NIH3T3 and A549 cells. *Swertia chirayita* and nintedanib reduced TGFβ1-elevated (**e**) EGFR levels. The values are represented in mean ± SEM, ***p < 0.0005, **p < 0.005, *p < 0.05 vs. TGF (*n* = 3). NC- normal cell, TGF-TGFβ1, Nin- nintedanib, and SC- *Swertia chirayita*.

### Western blot analysis

A549 and NIH3T3 cells were stimulated with TGFβ1 to induce mesenchymal transition and proliferation of fibroblasts. The levels of TNF downstream indicators NF-κB and its phosphorylation were significantly decreased through TNF/NF-κB signaling when cells were treated with SC and nintedanib, as illustrated in Fig. 14 (1) and (2). NF-κB (p65) levels have been significantly decreased by nintedanib with TGFβ1 (0.47 ± 0.12), nintedanib (0.34 ± 0.13), SC with TGFβ1 (0.52 ± 0.11), and SC (0.51 ± 0.08) in A549 cells. In A549 cells, nintedanib with TGFβ1 (0.37 ± 0.10), nintedanib (0.25 ± 0.05), SC with TGFβ1 (0.26 ± 0.08), and SC (0.41 ± 0.09) all significantly lowered the phosphorylation of NF-κB (p65) that inhibits EMT. In NIH3T3 cells, SC (0.52 ± 0.07) and nintedanib (0.65 ± 0.05) treatment for TGFβ1-induced fibroblast proliferation showed a significant reduction of the TNF downstream marker NF-κB (p65). The p-NF-κB (p65) levels were significantly decreased in nintedanib with TGFβ1 (0.78 ± 0.12), nintedanib (0.64 ± 0.03), SC with TGFβ1 (0.66 ± 0.05), and SC (0.72 ± 0.03) treatments to inhibit TNF-α signaling via the canonical NF-κB pathway in fibroblast proliferation. TGFβ1 (2.23 ± 0.14) significantly elevated the EGFR levels in A549, and treatment with nintedanib (0.86 ± 0.38) and SC (0.46 ± 0.21) showed significant downregulation in TGFβ1-stimulated cells. A549 cells without TGFβ1 also showed reduced levels of EGFR with nintedanib (0.55 ± 0.23) and SC (0.55 ± 0.24) treatment due to the inhibition of TGFβ1 signaling and the EGFR inhibition activity of SC compounds. All three sets of western blot raw data were incorporated in the supplementary file S5, and uncropped complete blots were depicted in the Figshare database^58^.

## Discussion

Leveraging topological characteristics that take into account degree, betweenness, and closeness centrality, network pharmacology was applied to SC, and nine primary targets were found, including EGFR, MMP9, IL-6, AKT1, SRC, MAPK3, FGF2, TNF, and STAT3. The components of SC (MANG, GENPD, BELDF, SWNIN, DTPA, SWCIN, NSWIN, CHIRT, and SWIDE) exerted a good binding affinity with the respective core targets, as represented in Table 3. In the case of IL-6 and AKT1, fewer interactions and binding affinity were observed with all the components except MANG. Compared to other components, MANG, BELDF, and GENPD of SC demonstrated strong docking interactions with all proteins. Thus, they were considered for the MD simulation with a specific pose derived from the IFD (Table 7). The protein-ligand contact timeline throughout the 100 ns MD simulation period remains stable, and the interactions were conserved in all the complexes, as shown in the 2D docking interaction Fig. 5.

Figure 6 demonstrate that the compounds obtained steady RMSD, maintained the structural compactness of proteins by reducing the fluctuation in the RMSF graph, ligand radius of gyration (rGyr), and detected the ligand intact in the protein binding pocket with less solvent exposure than expected by the SASA. The BELDF interaction within the protein binding sites of EGFR, TNF-α, and MAPK3 exhibited a good binding affinity, within the limits of RMSD variations, with reduced solvent exposure and slight flexible loop fluctuations at no binding sites, indicating a stable complex. The MD simulations of MANG with all proteins revealed stable interactions at the binding site, characterized by minimal fluctuations, as indicated by RMSD, RMSF, and solvent exposure. However, the MANG with TNF-α interactions was slightly fluctuating during the last five nanoseconds of the simulation period. The MD simulations of GENPD with proteins showed stable interactions. The more water exposure and rGyr for the GENPD and EGFR complex did not show any significant deviations from the protein ligand complex. Further confirmation of these complexes’ stabilization was provided by PCA analysis, which confirmed the stable conformational states with dynamic transitions. These findings support the possibility that the biological activity of all the complexes is related to their conformational flexibility, with energy minima indicating stable ligand-receptor connections. All the protein-ligand complexes exerted a lower free energy state and single and multiple stable conformations, as represented in the free energy landscape Fig. 10. The RMSF study suggests that for residues with low RMSF values and little motion, these positive correlation patterns in DCCM analysis enhanced complex stability and decreased residue fluctuation. Molecular docking and dynamics of SC with the core targets confirmed the regulation of major targets as predicted by the network pharmacology.

Regulating AKT1 as one of the central nodes by SC inhibits the senescence of alveolar epithelial cells and promotes ATII to ATI differentiation by regulating the release of profibrotic markers TGFβ1 and MMP9^59^. TNF-α, in conjunction with TGFβ1 signaling, promotes EMT synergistically to induce fibroblast migration and proliferation^60,61^. The inhibition of downstream NF-κB activation by TNF-α signaling regulation is reported in the suppression of fibrotic foci formation^7^. The regulation of TNF-α also inhibits the induction of MMP9, which contributes to the ECM remodeling and fibroblast proliferation^62^. The regulation of EGFR by SC through the EGFR signaling pathway inhibits crosstalk with TGFβ1, thereby suppressing EMT and the proliferation of fibroblasts^63^. Further regulation of SRC by SC inhibits myofibroblast activation, EMT, and inflammation^64^. The regulation of IL-6, TNF-α, STAT3, and MMP9 by SC suppresses inflammatory responses, fibroblast activation, and ECM remodeling, which helps to inhibit fibrotic foci formation^62,65^. The regulation of IL-6, HIF-1α, and STAT3 by SC through the HIF-1α signaling pathway typically inhibits the cellular response to hypoxia, thereby facilitating the restoration of respiratory function^66^. SC acting on the HIF-1α signaling pathway affects the PD-L1 expression and MAPK signaling pathways to decrease the interleukin production and hypoxia^67,68^. Inhibition of other AGE-RAGE and FoxO signaling pathways regulates the positive feedback signaling of inflammatory reactions, oxidative stress resistance, and the DNA repair process in the damaged epithelial cells^69,70^. The interaction of these targets and pathways with interlinked biological processes, including EMT, fibroblast proliferation, differentiation, and inflammation, reveals a complex pathophysiology. Modulation of these networks by SC, as explored through network pharmacology, provides more significant insights into the treatment of pulmonary fibrosis. The core targets actively interact with major pathways in pulmonary fibrosis, including TNF, HIF-1, FOXO, AGE-RAGE, PD-L1 expression, VEGF, IL-17, relaxin, ErbB, and EGFR tyrosine kinase inhibitor resistance pathways. However, major significant targets, such as TNF, MMP9, IL6, AKT1, and MAPK3, were primarily involved in TNF-α signaling^62,71,72^. The stable MD simulation results of SC on these core targets also highlight the regulation of TNF-α signaling. The SC, acting on TNF-α through its downstream pathway NF-κB, is considered a central regulator of EMT, fibroblast proliferation, myofibroblast differentiation, and inflammatory reactions that contribute to ECM deposition and the formation of fibrotic foci^7^. The crosstalk between TGFβ1 and the SC-regulated signaling pathways underscores the role of TGFβ1 signaling pathways in regulating fibrosis progression.

Considering the mechanism of action of SC, in vitro models were developed to assess its efficacy in regulating the reported network of pathways and biological processes. The EMT model was standardized by stimulating A549 cells with TGFβ1^73^. The EMT cells exhibited low expression of E-cadherin, whereas SC treatment prevented EMT by preserving E-cadherin levels. The inhibition of TNF/NF-κB and TGFβ1 signaling by SC in A549 confirms the regulation of EMT and the differentiation of ATII to ATI through the regulation of crosstalk between the EGFR signaling pathway and MMP9 induction^8^. Additionally, the inhibition of EMT by SC reduces the migration and proliferation of fibroblasts into the extracellular space by half^74^. The inhibition of TNF/NF-κB by SC in the NIH3T3 cell migration assay confirmed the regulation of fibroblast migration and proliferation. SC inhibited the differentiation of myofibroblasts in TGFβ1-stimulated fibroblast cells through the TNF/NF-κB and TGFβ1 signaling pathways. The inhibition of TNF/NF-κB also regulates the HIF-1α signaling and IL-6 release, helping to reduce inflammatory reaction and hypoxia^75,76^. The multitargeted effect of SC confirmed the regulation of the fibrosis-inducing network by acting on primary targets, pathways, and biological processes, which helps maintain alveolar homeostasis and reconfigure fibrotic foci. The current study highlights that the appropriate application of network pharmacology provides greater insight into predicting mechanisms with molecular-level evidence, which could assist in reducing the time spent on expensive and futile techniques. Regulation of fibrotic foci formation through TNF/NF-κB and TGFβ1 signaling pathways by SC offers a new therapeutic approach for treating pulmonary fibrosis. The current study is limited to in vitro cell culture study validations; in the future, extending it to in vivo preclinical studies can further confirm the SC’s efficacy and safety through dynamic, kinetic, and toxicity studies. Extending the comprehensive mechanism exploration in pulmonary fibrosis animal models lends further support to the current predictions and validations. However, this work can aid future preclinical research by ensuring data integrity, which is crucial for addressing potential regulatory issues that may arise for traditional medicines. In the future, Bellidifolin can be isolated and explored as an anti-fibrotic agent for the treatment of pulmonary fibrosis.

## Supplementary Information

Below is the link to the electronic supplementary material.

Supplementary Material 1

## Data Availability

Additional data for this work were provided in the Figshare database. The project contains the following extended and underlying data. 10.6084/m9.figshare.28225982.v3. The raw data of the chromatograms were included in supplementary file 1.Raw data, including the target identification and functional annotations, were incorporated in supplementary file 2.Cytoscape raw figures, IFD interaction matrix figures, and MD simulations data were included in supplementary file 3.XP-docking raw results were included in supplementary file 4.All 3 sets of Western blot analysis were included in supplementary file 5.Original full-length blots and raw software data were depicted in the Western blot raw data file.All network pharmacology analysis of *Swertia chirayita* using Cytoscape was represented in the **Swertia chirayita** LCMS compounds PPI network file.In vitro statistical data using GraphPad Prism were provided in the in vitro raw data file.All the software and data-related documents for in silico were provided in the molecular docking, MD, and PCA raw data file.
