# Supplementary material for: Network pharmacology and in vitro studies demonstrate modulation of fibrotic pathways by Swertia chirayita in pulmonary fibrosis
Source: Sci Rep. 2025 Dec 8;16:1150. doi: 10.1038/s41598-025-30784-x (PMC12789668; doi:10.1038/s41598-025-30784-x)
Supplement: Supplementary file 1 — Supplementary Material 1 [file 41598_2025_30784_MOESM1_ESM.pdf]

Network pharmacology and in vitro studies demonstrate modulation of fibrotic pathways by *Swertia chirayita* in pulmonary fibrosis

Bharath H B[1], Farmiza Begum[1,2], Gautam Kumar[1,3], Jyothi Giridhar[4], Usha Y Nayak[5], Fayaz S M[6], Pawan Ganesh Nayak[1], Yogendra Nayak[1*]

[1]Department of Pharmacology, Manipal College of Pharmaceutical Sciences, Manipal Academy of Higher Education, Manipal, Karnataka 576104, India.

[2]Department of Pharmacology, Vaagdevi Pharmacy College, Bollikunta, Warangal, Telangana 506005, India.

[3]Amity School of Pharmaceutical Sciences, Amity University, Mohali, Punjab 140306, India.

[4]Department of Pharmaceutical Chemistry, Manipal College of Pharmaceutical Sciences, Manipal Academy of Higher Education, Manipal, Karnataka 576104, India.

[5]Department of Pharmaceutics, Manipal College of Pharmaceutical Sciences, Manipal Academy of Higher Education, Manipal, Karnataka 576104, India.

[6]Department of Biotechnology, Manipal Institute of Technology, Manipal Academy of Higher Education, Manipal, Karnataka 576104, India.

* Corresponding author: Yogendra Nayak

E-mail address: yogendra.nayak@manipal.edu

Supplementary file 1

S1-1: Mass spectra of Dl-aspartic acid (134.07)

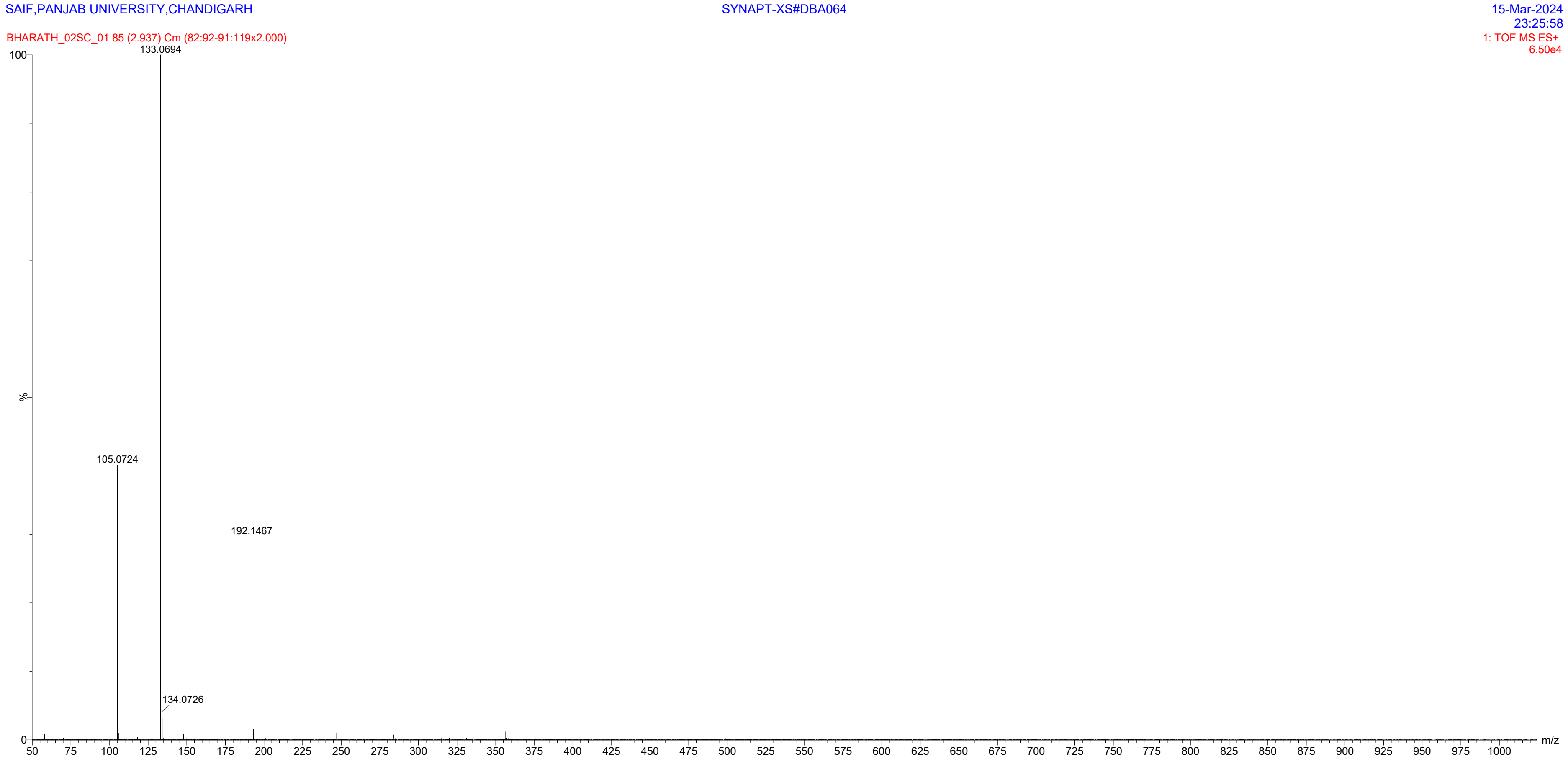

S1-2: Mass spectra of Chiratenol (427), Enicoflavine (211.15), Gentianine (133.10, 145.10, 105.07, 176), L-leucine (132, 85), L-threonine (119.09), Palmitic acid (257.15), Stearic acid (285.17), Syringaresinol (419.19), and Taraxerol (353)

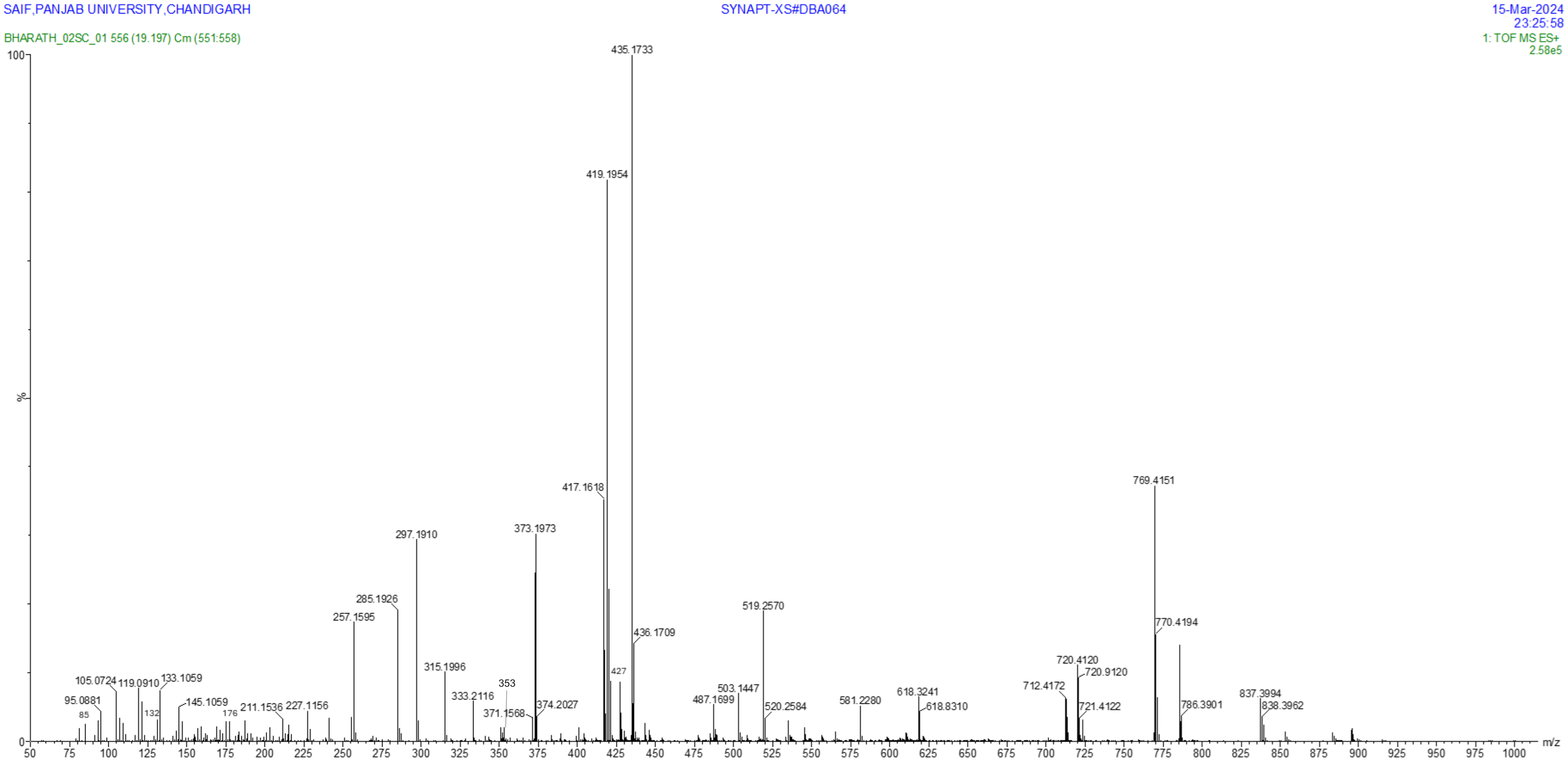

S1-3: Mass spectra of (3E)-3-(aminomethylidene)oxane-2,4-dione (140.92) and Glutamate (84.95)

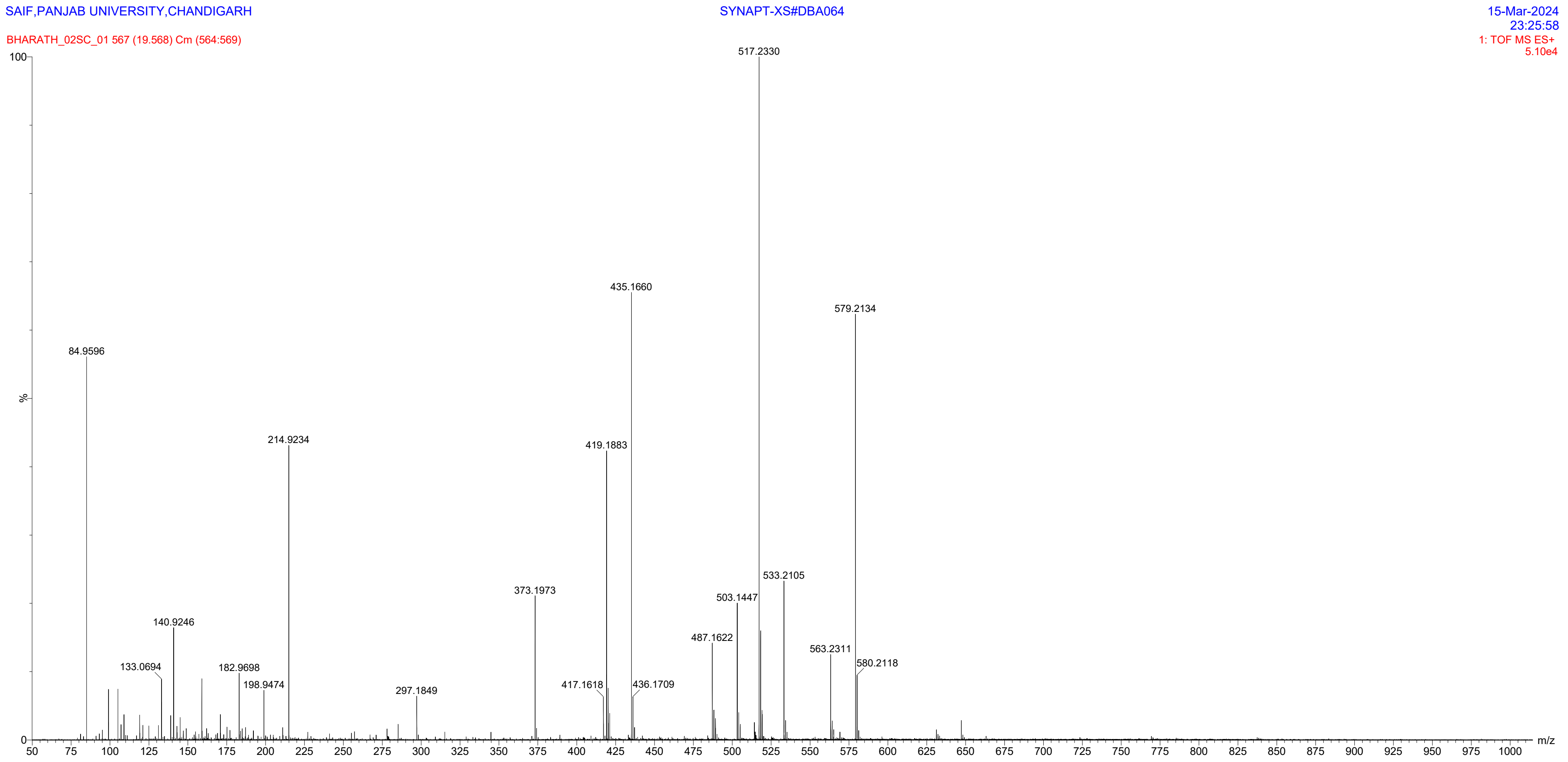

S1-4: Mass spectra of Bellidifolin (191.11), Chiratol (289.21), Demethylbellidifolin (260.12, 219, 81), Dl-tryptophan (205.12), and Stigmasterol (159)

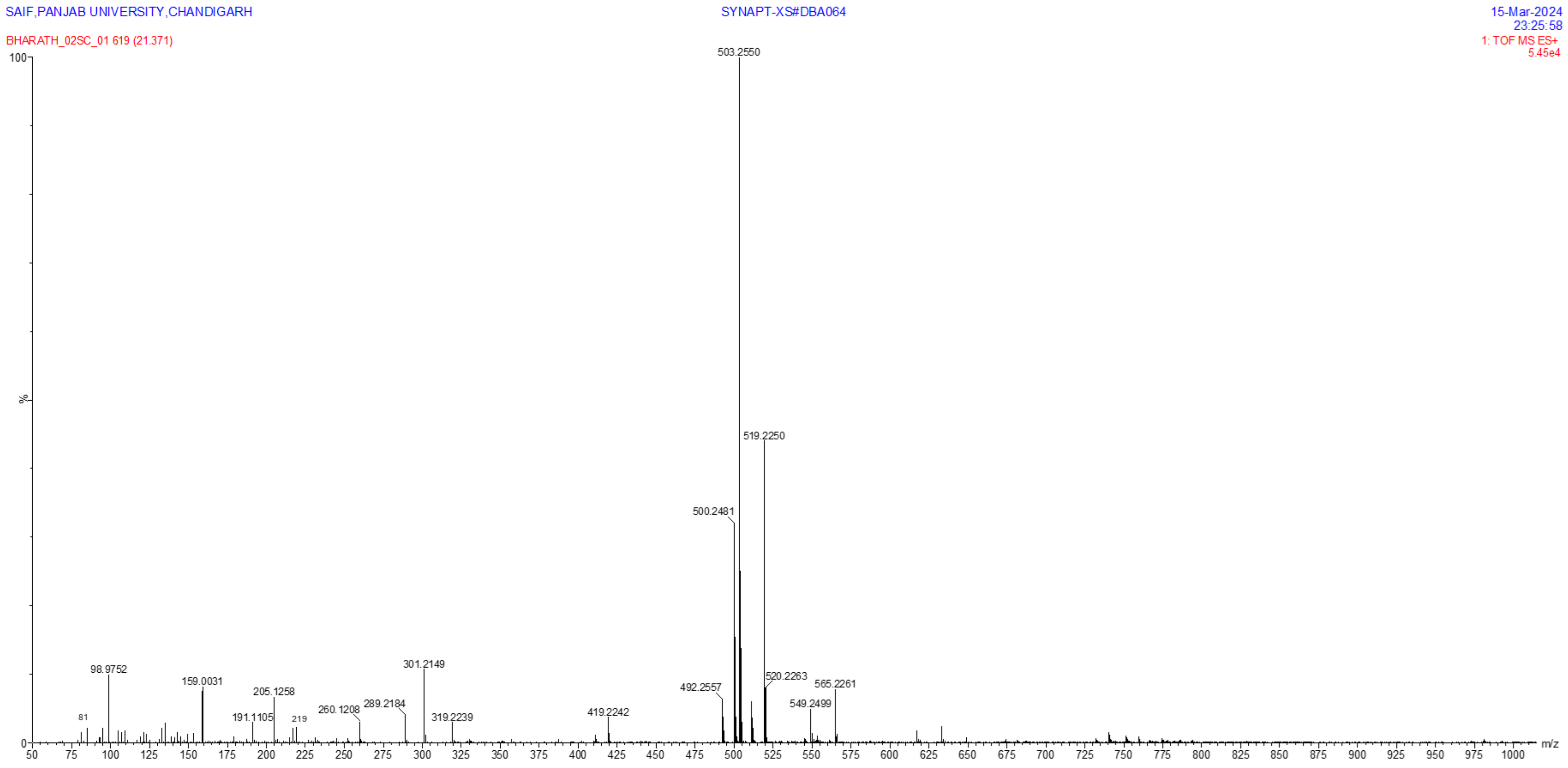

S1-5: Mass spectra of Swerchirin (287.2)

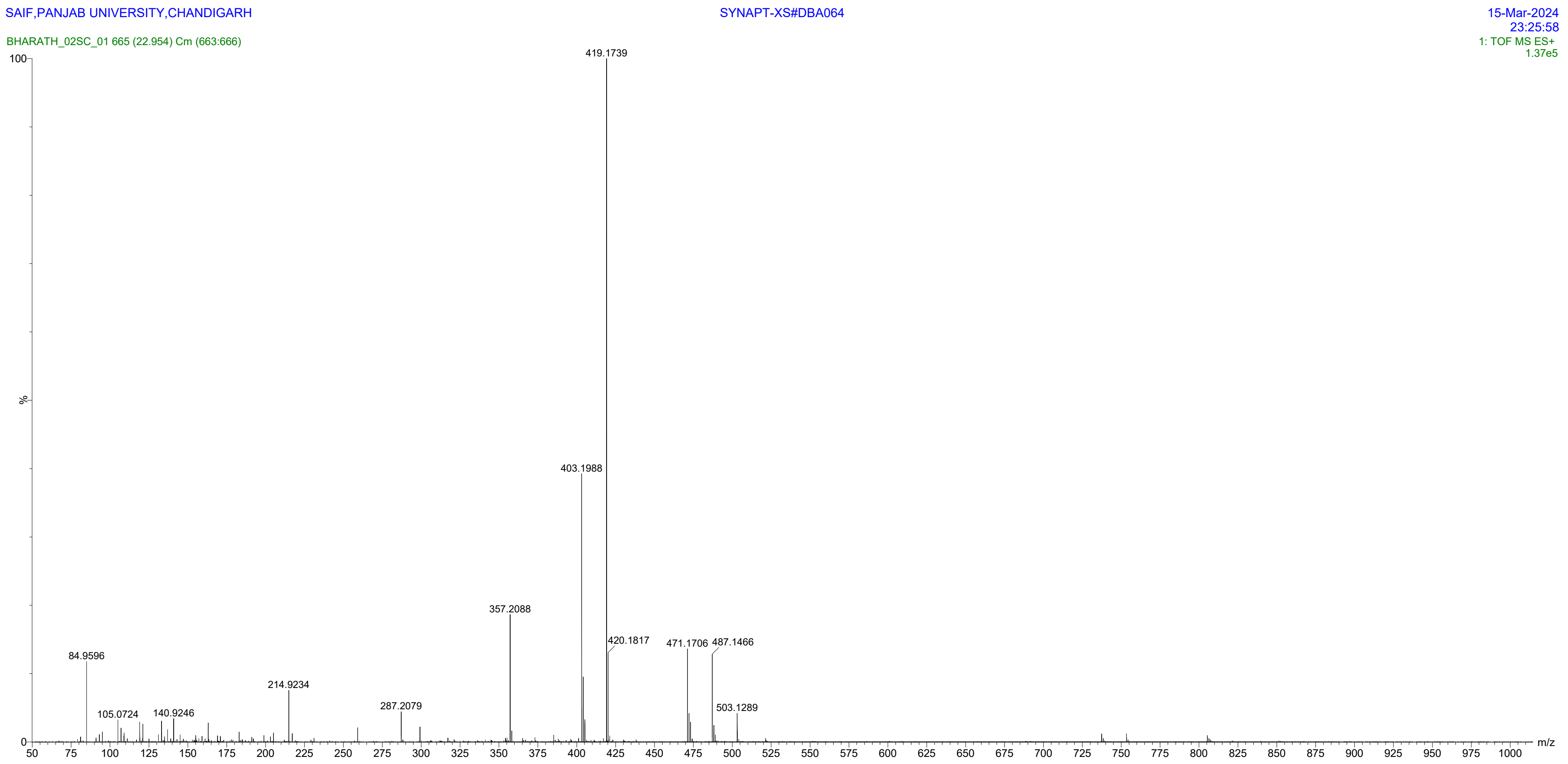

S1-6: Mass spectra of 5,8-dimethylbellidifolin (145.1), Amaroswerin (227.11, 203, 201), Calendol (352.17, 353), and Swertiamarin (355.18, 119, 375)

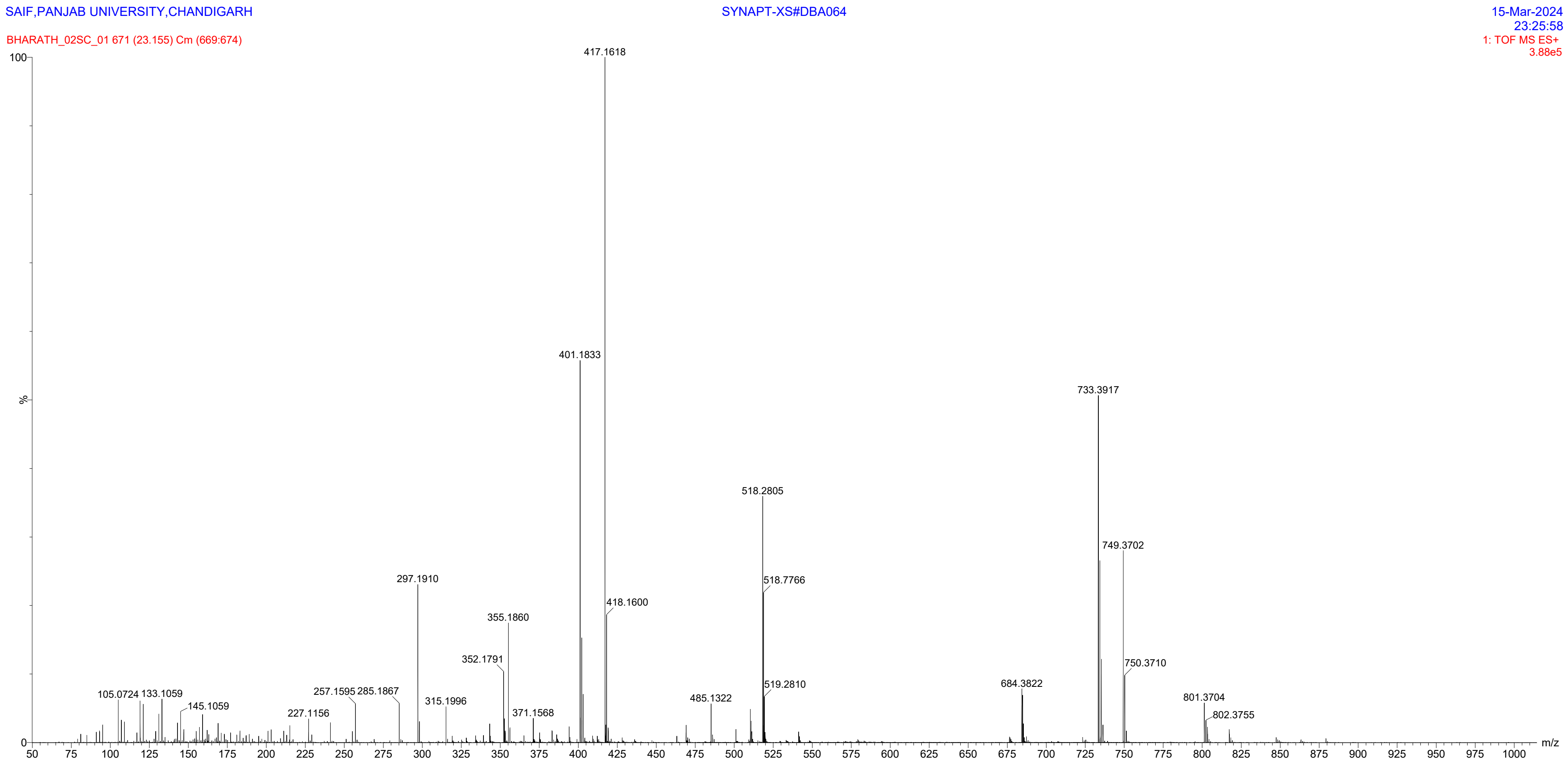

S1-7: Mass spectra of Amarogentin (198.94)

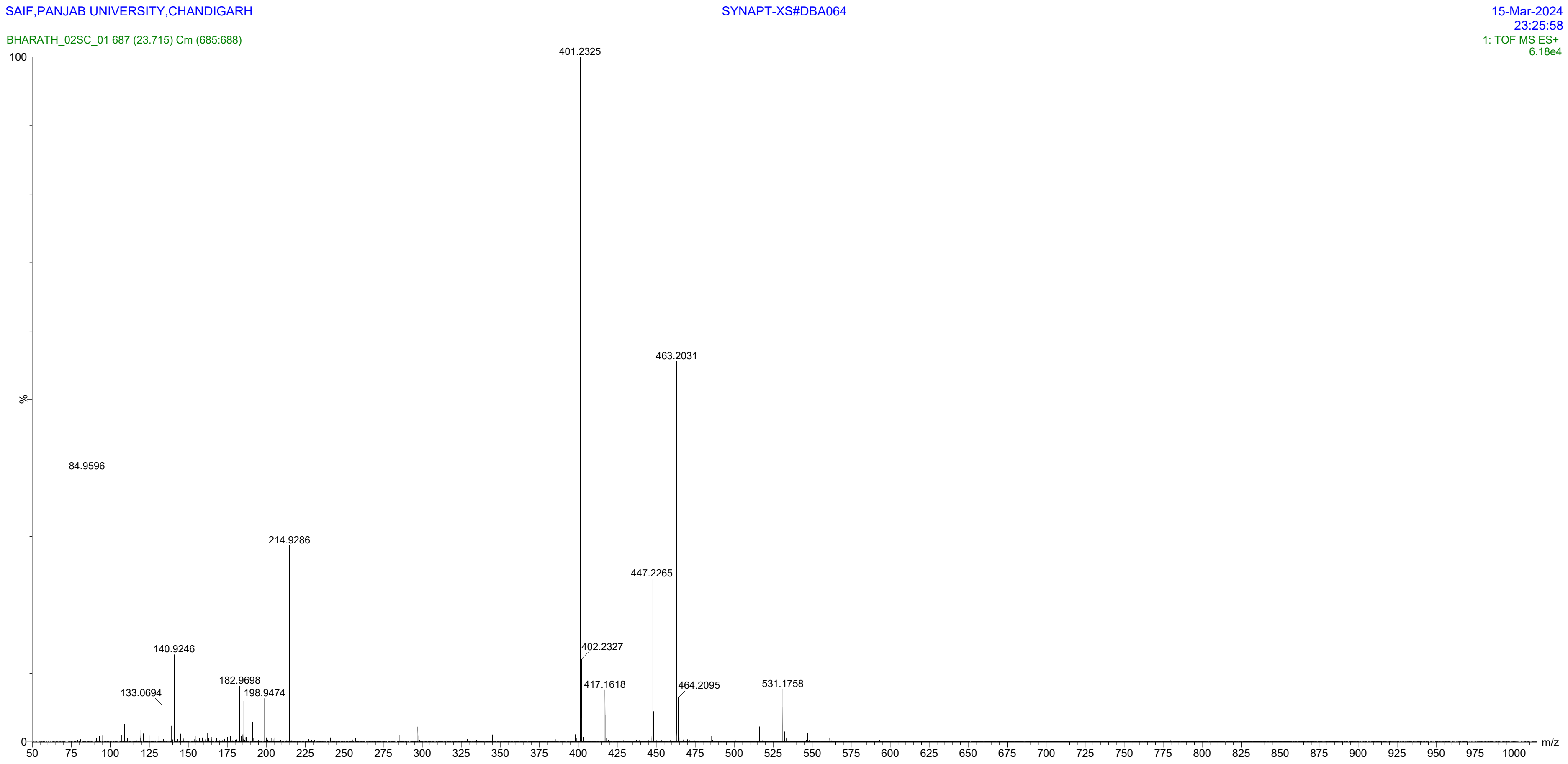

S1-8: Mass spectra of Pichierenyl acetate (468) and 2,5-Dihydroxyterephthalic acid (198.94)

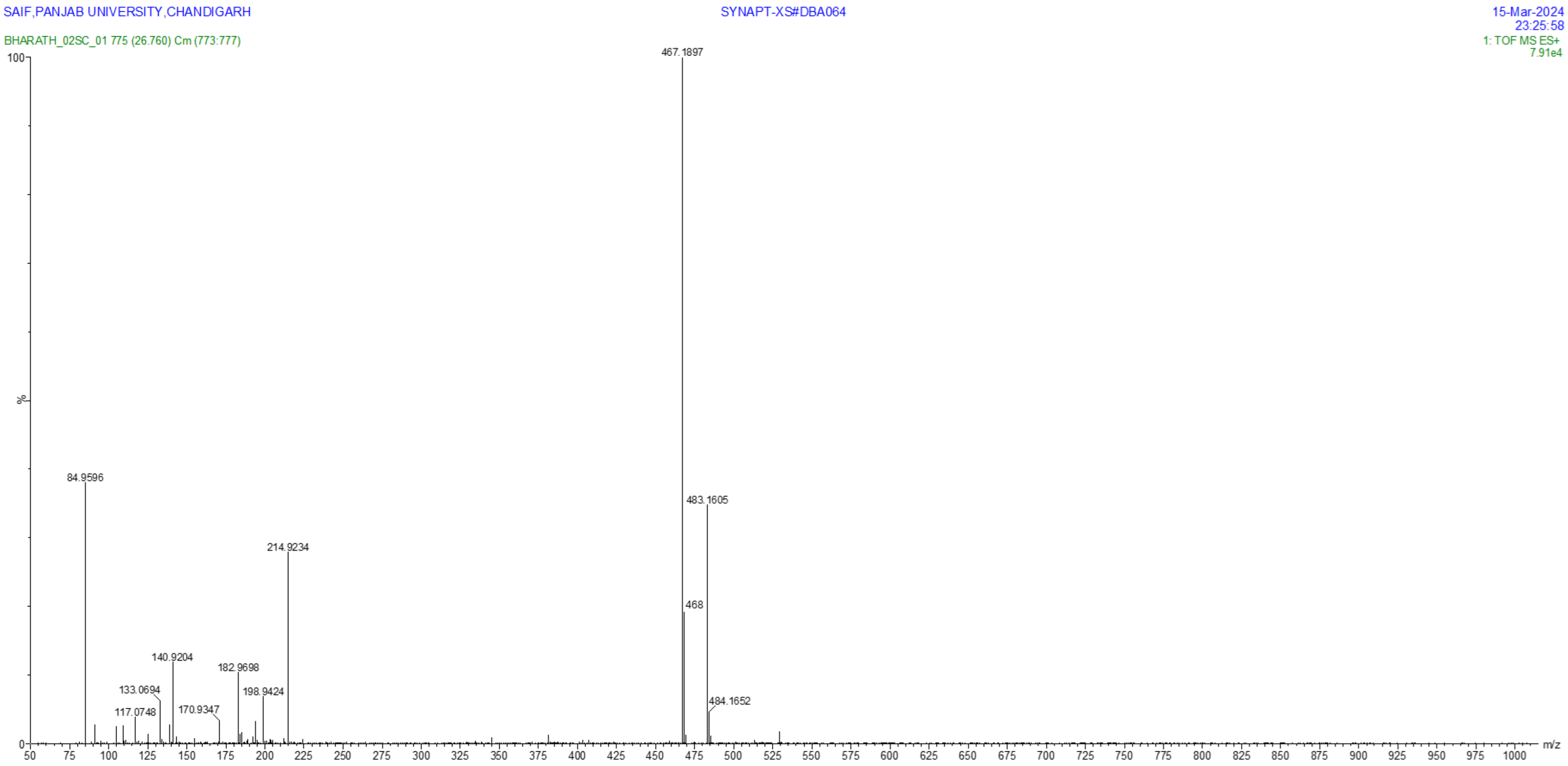

S1-9: Mass spectra of Octadecanoate (284.07) and Sweroside (359.11)

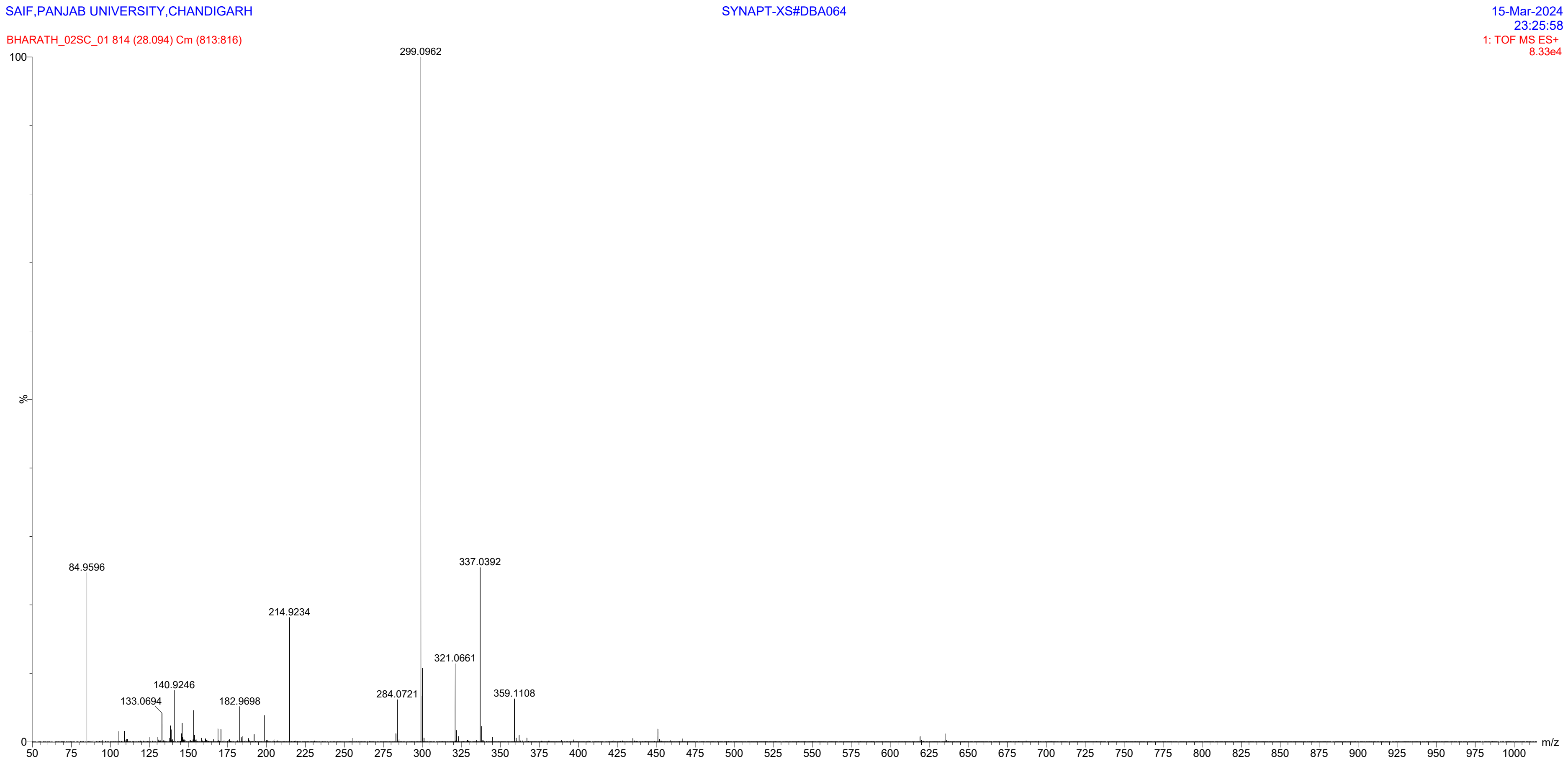

S1-10: Mass spectra of Decussatin (303, 301.2) and Swertinin (415.5)

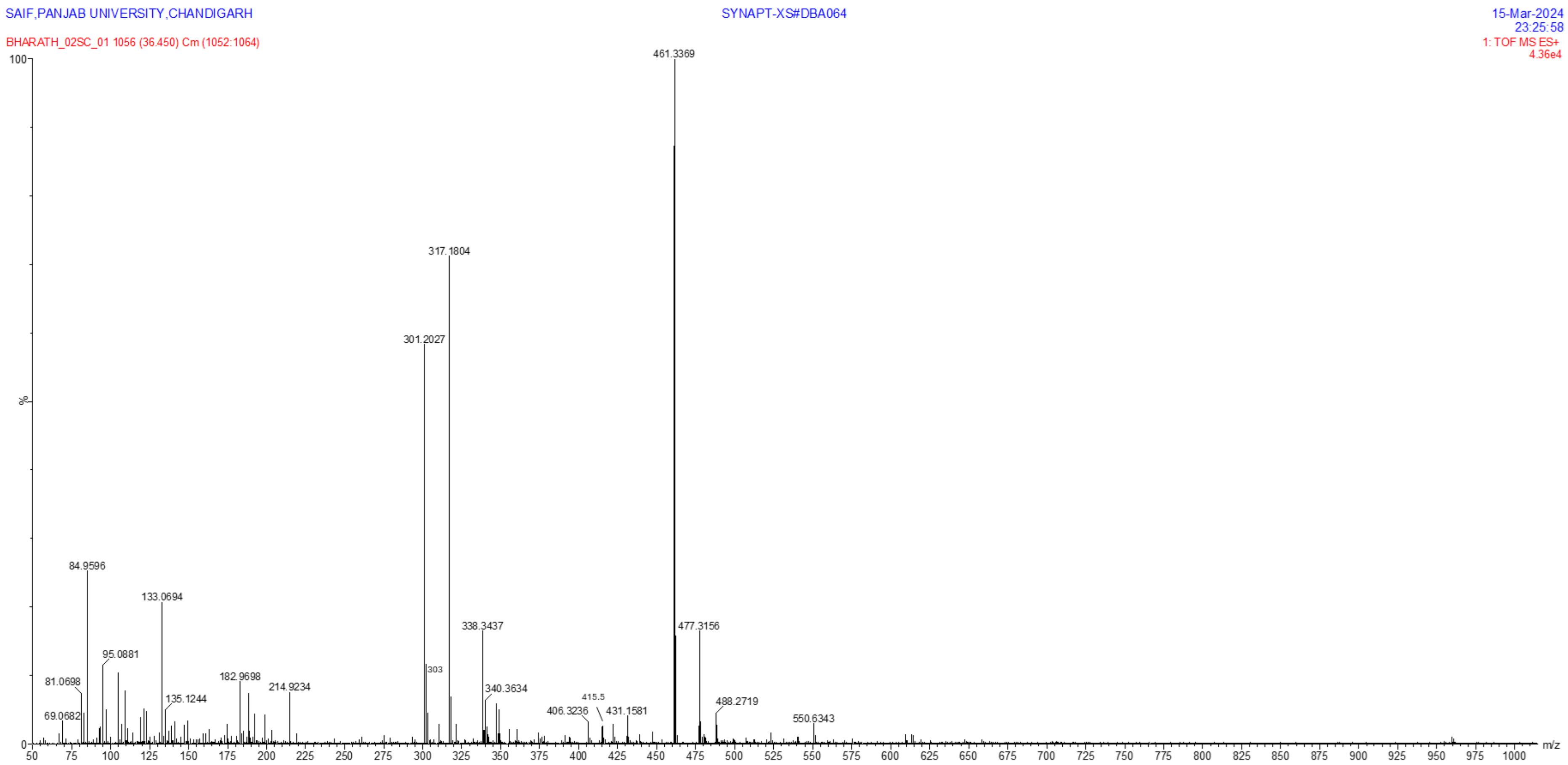

S1-11: Mass spectra of Erythrodiol (443), Gentiopicroside (149.13, 121), Mangiferin (303.21, 339.34, 406), Swertiapuniside (599.42), Beta-Sitosterol (397.37, 119), and Isovitexin (433.17, 313)

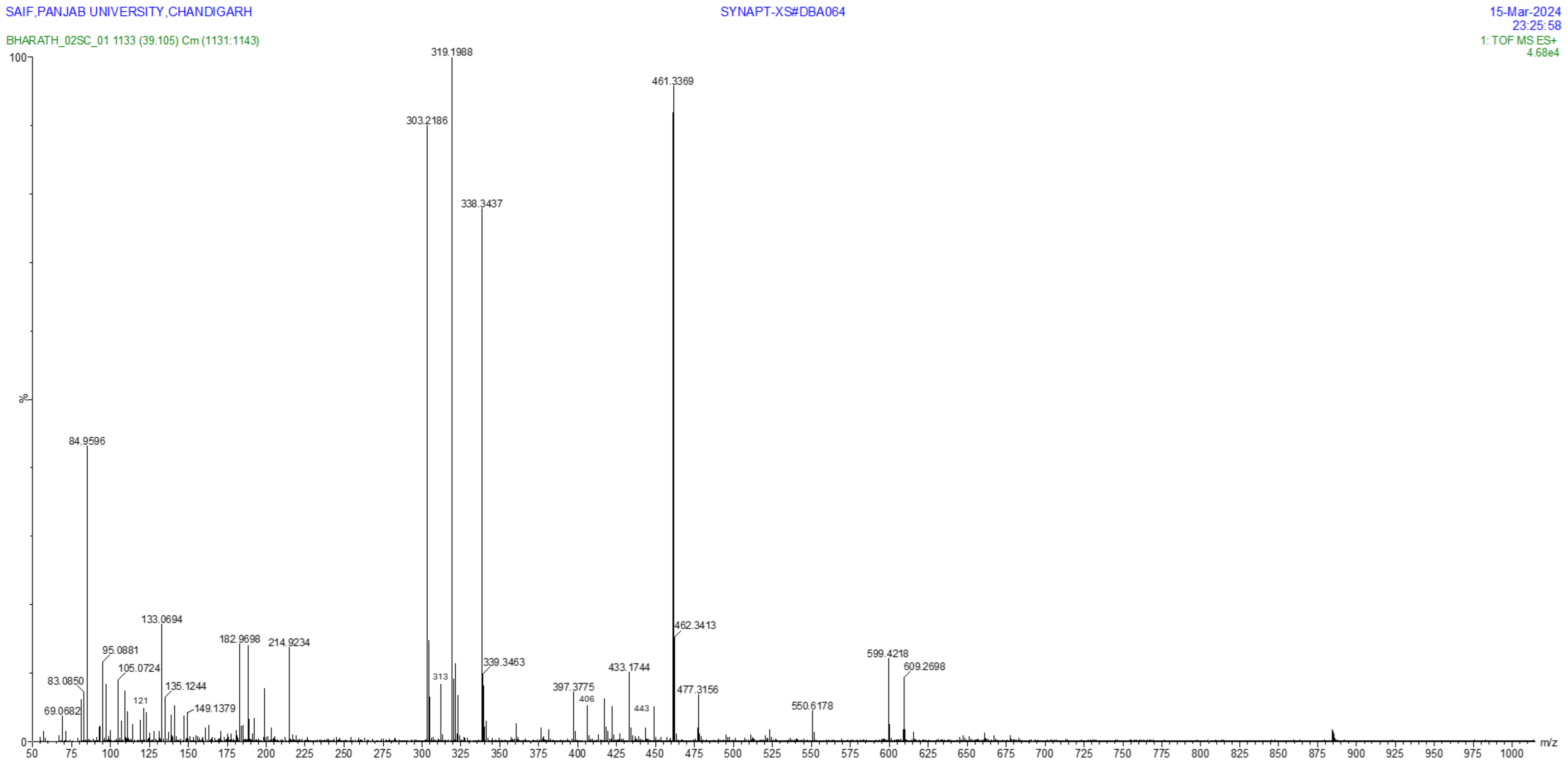

Supplementary file 2

S2-1: Disease targets (IPF)

**Disease targets**							
TNF	TWIST1	ROS1	CYP2A6	NLRP3	AGTR1	DICER1	SMARCA4
TP53	KRT20	CD36	LMNA	NOS2	CDKN2B	COL3A1	CYP1A1
IL6	RBM45	GLI2	IRF1	EGF	PLAU	TNFRSF8	BMP2
VEGFA	LGALS3	HSPA5	CTLA4	SERPINE1	CREBBP	PDGFB	KCNQ1
IL1B	IGF1R	IGFBP3	IGF2	H3P10	CCR2	TEK	HBB
IL10	AIMP2	COPD	NOS3	AGT	NOD2	BRAF	KIF21A
TGFB1	FBN1	F2R	ABCA1	FAS	RPE65	BRCA1	IL2RA
IFNG	NFKB1	TGFBR1	EDNRA	IL18	MRC1	SFTPC	MIR18A
PIK3CA	HSPA4	EP300	MLH1	TLR2	MIR34A	MET	PPP2R1B
CRP	ELN	SPARC	RYR1	CXCR4	CEACAM5	RTEL1	BMP6
BCL2	CRK	JUN	SRC	ICAM1	BMP4	NRAS	KDR
EGFR	GRAP2	C3	MIR205	FGFR2	COX1	MAP2K1	INPP5E
CTNNB1	POLDIP2	ALOX5	STK11	MIR21	CHI3L1	CASP8	MSH2
PTEN	CD40	ANGPT1	MIR20A	FN1	ABCG2	INS	INSR
MMP9	FOS	HDAC9	MIR200A	HMGB1	JAG1	SMAD4	SETD2
CDKN2A	AHSA1	LPA	CD4	REN	TNFRSF1B	CDH1	NR3C2
CXCL8	RNF19A	TXN	F2	KIT	APOA1	ATM	MIRLET7B
AKT1	NOS1	FHIT	ERCC2	HLA-B	RAC1	PPARG	MAPK3
PTGS2	MAPK8	UVRAG	NF1	TERT	HFE	ALB	MIR34B
KRAS	ALK	ANXA1	LOC11167447	MTCO2P12	XRCC1	ALB	TTN
IGF1	CCN2	CXCR2	ENG	PTPN11	VCAM1	PARN	MIR183
STAT3	CCL5	BRD4	CDK4	IL1RN	RUNX2	MUC5B	SOX9
TLR4	GPT	KL	ELANE	NOTCH1	DECR1	RB1	TMEM67
CCL2	TGFBR2	CKAP4	EGFR-AS1	CSF3	FASLG	PRKN	GJB2
PIK3CD	VWF	LOX	MIR29C	EDN1	HSPB1	FGFR1	MEN1
ACTB	PDCD1	MMP13	AR	ADIPOQ	TLR3	HRAS	LPL
ESR1	DNMT1	HSPB3	VHL	SIRT1	CNR1	FGFR3	TGFB2
PIK3CG	PDGFRB	ETS1	TSC1	HLA-A	BCHE	MIR17	CEACAM6
PIK3CB	CDKN1A	TIMP3	MIR93	HGF	GDF15	MYC	BIRC5
ACE	IL33	PRKAA1	SOD1	HMOX1	SOAT1	ERCC6	SLC9A3
IL17A	COL1A1	E2F1	MIR141	MECP2	GLB1	SFTPA1	PLA2G6
MAPK1	TNFRSF1A	COL18A1	SHH	BRCA2	FOXO3	SFTPA1	LOC11
HIF1A	RELA	SDC1	SLC2A1	MPO	POSTN	APOE	BAX
SMUG1	SERPINA1	FOXM1	CP	EZH2	EGR1	APC	CEP290
CSF2	CFTR	RTEL1	MIR19A	VIM	TGFA	MIR126	MIR486-1
MMP2	MMP3	XIAP	TUBB3	JAK2	VIP	NKX2-1	SFTPD
HLADRB	SMAD3	MMP7	COL1A2	FGF2	NT5E	MIR221	MIR30D
CD274	RAF1	CCR7	STN1	CAV1	WNT5A	MIF	MIR107
IL1A	HP	PTK2B	PDGFRA	GABPA	YAP1	DSP	MARS1

IL4	NR1I2	PTGS1	SLC11A1	CXCL10	BECN1	CFTR-AS1	HMGA2
ERBB2	TTR	SMN1	SCNN1A	CXCL12	DKK1	SFTPA2	FAM13A
BDNF	SYT1	PRKAA2	KNG1	MAPK14	CST3	SCN1A	GNAS
MTOR	WNK1	ADAM17	GSTM1	RET	GLI1	STAT1	GBA
IL2	ANGPT2	STAT4	HLA-DQB1	PTCH1	TNC	SFTPB	ERCC1
ABCB1	TLR9	SOCS1	MIR130A	TIMP1	CD163	MIR125A	GSTP1
LEP	TRBV20OR9	TGM2	GJA1	MUC1	GORASP1	MIR200B	CEACAM3
COX2	HSP90AA1	DNMT3B	MIRLET7D	NR3C1	CXCR3	MIR29A	MIR146A
CCND1	CD68	ZEB1	ADRB2	MMP1	MIR145	MIR222	APOB
SPP1	MS4A1	S100A4	CDKN1B	IL13	CD28	MDM2	MTHFR
NFE2L2	AGER	KLF4	SLC22A18	PLG	RAD51	FCGR2A	MIR15A
CASP3	TNFSF10	FGF23	STX1A	MIR155	S100A9	TSC2	TFRC
TP63	NTRK1	RETN	PKD1	PCNA	LGALS1	MIR200C	PARP1

S2-2: SC compounds targets

Merged targets of all compounds							
HSD17B10	ITK	DYRK1B	MAP3K8	MIF	LCK	F2	GSTM2
APEX1	ADRB1	CCND1	EPHB4	CFTR	SRC	CTSB	GGH
TTR	MARK4	ALDH2	LYN	HTR2C	KDR	WDR5	GLB1
PTPN11	MGLL	INSR	FNTA	GRIA2	MAP3K9	HTR7	TYRO3
PTGER1	SOS1	TEK	PTP4A3	KCNA5	FGFR1	CPT2	ITGAV
TRIM24	PKM	HSPA1A	DAO	TFPI	AURKA	F7	ITGA2B
NFKB1	GRM8	NUAK1	GSK3B	PSMB2	BTK	BRD4	RELA
THRA	GRM3	HDAC6	ERBB2	HSP90AA1	MCHR1	HDAC11	CALM1
CTSD	GRM1	GRB7	CRHR1	MAOA	FUCA1	S1PR2	BCL2
AKR1C2	GRM5	EIF2AK2	DRD3	KEAP1	ACE	ABCB1	SLC6A3
RAB9A	GRIK5	CA4	ADRB2	METAP2	TNF	TDO2	CCNA2
NR4A1	GRIA4	ADAMTS5	PTK2	LDHB	FUT6	GHSR	FASN
BLM	LMNA	ADAMTS4	AKT1	PDGFRB	CASP1	BRDT	PTGER3
SLC6A5	NR3C2	GRK2	TNNC1	KDM1A	EIF4H	PDE7A	PTGDR
SLC2A1	PTPN1	ALPG	HSD17B3	GRIN1	PABPC1	CLK2	STS
RORB	FABP4	PLAA	SNCA	ANPEP	ADH1A	PRMT6	SLC5A11
NPC1	HDAC2	GRIA1	FEN1	KIF11	EIF4A1	CDK7	BAD
GLRA1	CHRM1	GRIA3	GSTA1	ACVRL1	PIK3CA	CARM1	ERAP2
KLF5	CCNE1	MET	IGFBP6	DRD1	PTGS2	HPSE	NR2E3
PSMB1	SCN9A	NOTUM	IGFBP4	SLC1A3	NADK	TNIK	EPHX1
PTPN2	CHUK	QPCT	IGFBP5	P2RX4	VARS	HDAC4	CHKA
MAPK1	CHRM3	HMGCR	IGFBP2	GRK5	LARS	FBP1	NAMPT
CYP3A4	PRKCD	CYP51A1	IGFBP1	PDE3B	MAP2	GSK3A	DHFR
CHRNB4	ACHE	AR	PTPRS	CBX4	PPARA	HTR2A	GBA1
CLK4	PRKCA	NPC1L1	MAPKAPK5	EIF2AK1	EGFR	SIRT1	EZH2
FPR1	SCD	CYP17A1	F10	GBA2	HDAC1	MERTK	FKBP1A
SAE1	FGR	RORC	COMT	AXL	TARS	ICMT	PSMB5

AKR1C1	HSD11B1	SHBG	CES1	GGPS1	BCHE	FOLH1	APH1A
CDK5	CHRM2	SREBF2	TEC	NOS2	CYP19A1	GAK	CAMK2D
NR1I2	DHODH	CYP2C19	MAP4K4	CCR1	CHIA	FAAH	TAOK1
KDM6B	GPR17	SLC6A2	GSTP1	KLK1	SELL	PPARG	LDHA
CSNK2B	KDM4C	RORA	HDAC3	NAAA	SELE	PDE4D	TNK1
PTGER2	TLR8	SERPINA6	CETP	ZAP70	SELP	CD81	DNM1
TDP1	ITGB1	SLC6A4	AHCY	ACACB	DUT	FFAR1	DBF4
ALOX12	RPS6KA3	G6PD	CD38	PTGS1	YES1	TBXA2R	MAPK9
PREP	FPRL2	NR1I3	PRMT1	CASP8	GCK	LTB4R	YWHAG
GPR6	HDAC9	VDR	NTSR1	CYP2A6	ENPP1	PGR	JAK3
CAMKK2	PLA2G2A	CES2	MINK1	OPRD1	NTRK1	CYP11B1	JAK1
DPP9	PROC	CDC25A	NT5E	AOC3	THRB	CYP11B2	RAF1
DPP8	MC4R	DHCR7	PRMT5	MAP3K5	CCNA2	F11	NEK1
LGALS3	HDAC5	PPARD	LNPEP	PIK3CD	PTGFR	FNTA	HSD17B7
CHRM5	P2RX7	SQLE	FYN	SLC40A1	HSPA8	CTSK	SRD5A2
MAP2K2	KDM4A	PTPN6	CREBBP	GPR35	HSPA5	CTSS	GABBR1
CDC25B	HDAC10	FDFT1	GABRA1	PRSS1	OPRK1	P2RY1	PAOX
TOP2A	STING1	NR3C1	KDM5A	TOP1	KCNH2	ALOX5AP	NR1H4
GUSB	SLC1A1	SHH	GPR84	SPHK1	MAP3K14	APP	GABRA2
STAT1	SLC5A1	UGT2B7	CSNK2A2	S1PR3	MAP2K1	PRKCZ	HRH1
MDM4	MMP2	HSD11B2	KDM5C	ARG2	SIRT2	CDK5R1	IKBKB
DPP7	SLC28A3	DRD2	PTPRC	CTSG	CCNE2	TRPM8	PSEN2
CPT1B	IMPDH1	POLB	GABRB3	ADORA1	GAPDH	PDE4A	FABP2
HTR3A	ADORA2A	IDO1	CTRB1	S1PR4	BRAF	PDE4B	C3AR1
ADAM10	ADORA2B	MDM2	CHRNA3	DOT1L	TYMS	PDE4C	BRD2
NFE2L2	ADORA3	SIGMAR1	HPGDS	PIK3CB	PTGER4	MMP10	BRD3
EGLN1	SRD5A1	ATP12A	KDM5B	EP300	GBA	PIM2	HSD3B1
PDGFRA	MMP13	PTGIR	MCL1	ABCC1	HLCS	BMP1	SCN5A
EPHB2	MMP1	PRKCG	KLKB1	SCN4A	MMP14	CCNB1	CALCRL
FPR2	MMP7	PRKCB	ADRA2B	RXFP1	NQO1	TAAR1	CXCR3
ULK3	MMP12	PRKCE	CDC7	GRB2	MAPK8	CMA1	TSPO
PRCP	MMP8	PRKCQ	TGM2	TACR2	EDNRA	TRHR	ABCG2
CAPN1	SLC5A4	FABP3	GABRA2	PKN1	HK2	MAOB	NPY5R
TBXAS1	SLC5A2	FABP5	MALT1	P2RY12	HK1	KCNB1	NPSR1
SCN3A	HRAS	FABP1	DNMT1	CCR5	UPP1	ILK	AMY1A
PIK3R1	LGALS4	PTGES	DNMT3L	S1PR5	IL2	NISCH	AMY2A
CHRNA1	LGALS8	MAPK3	NUDT1	MTOR	ERN1	STK17B	NEU4
PIN1	CA2	AKR1B10	BRD9	DCUN1D1	NLK	MKNK2	NEU2
SLC9A1	CA1	PTPRF	NOS1	ITGB3	HSP90AB1	STK17A	FGF2
HPGD	CA12	PLA2G1B	HTT	CYSLTR2	TBK1	GCGR	ASNS
ERAP1	CA14	ACP1	KAT2B	AKT3	CDK5R1	CDK5R1	NMUR2
RPS6KA6	CA9	BACE1	JAK2	CHRM4	FLT1	PIM1	ADRA2A
NTSR2	TYR	HTR2B	TYK2	TMPRSS6	CA6	ROCK1	ADRA2C
PLAT	EPHX2	PTAFR	ADRA1D	TGFB1	CA5A	KCNA3	MGMT
CDC25C	SLC29A1	GRM2	CHRNA4	PRKACA	CA7	PIM3	FPGS
QRFPR	F3	BACE2	DUSP1	OPRM1	HCK	GRM4	CD22

F13A1	SLC28A2	PRKCH	KCNK2	CASP3	PI4KB	TUBB1	MAG
NOX1	PDE5A	F2R	KDM4E	APLNR	ESR1	TUBB3	NQO2
PDE3A	HCAR2	ALK	EPAS1	AKT2	ESR2	CCND3	GRIK1
PKMYT1	LGALS7	AVPR2	USP10	KLK5	ELANE	ALPL	GRIK2
DUSP3	MME	AVPR1A	USP13	PKN2	CBR1	CXCR2	GRIK3
TLR4	FHIT	MTNR1A	EGLN3	AAK1	CA13	PTK6	POLA1
CASP6	YARS	MTNR1B	BCL6	ACACA	FLT4	CCNE2	DNMT3B
CDK1	ST6GAL1	IL6	GAA	PSMB9	CA5B	SPHK2	EHMT1
IRAK1	CHEK1	MAPK14	GRK1	CFD	CSNK2A1	ASF1A	LYPLA1
SERPINE1	AGTR1	CNR1	OGA	BMP2K	IMPDH2	VCP	LYPLA2
PTPN7	MMP9	NOS3	ADA	ATG4B	STAT3	MAPKAPK2	ALOX15
XDH	CDK2	CDK4	LGALS9	TACR1	KCNMA1	CSNK1G1	CSF1R
RPS6KA1	ECE1	PAK4	MANBA	LTA4H	CCNE1	MKNK1	PCNA
STK3	PARP1	PTK2B	TK1	GPR55	PLA2G7	FAP	BDKRB1
KCNK9	ADK	HRH4	CDA	DPP4	CTSV	PLK1	KMO
PRKAA1	TNKS2	FCGRT	MGAM	CXCR4	ALOX5	MPI	PIK3C2B
ACVR1B	TNKS	AURKB	PYGL	PIK3CG	CTSL	AKR1B1	ROCK2
PLAU	IGFBP3	FFAR2	NGFR	CCR2	MMP16	SOAT1	F9
AVPR1B	AKR1C3	SCN2A	PYGM	KLK7	CHRNA7	SOAT2	PRSS3
HIF1A	TYMP	FER	RNASEH1	NR1H3	IGF1R	CASP7	SSTR5
HDAC8	PNP	MARK3	MAPK10	CA3	PCSK7	PDPK1	GPR142
GPBAR1	MMP3	HDAC7	ATIC	BMPR1A	CYP2C9	DYRK2	P2RX3
NTRK3	MARS	CLK1	CDK9	NR1H2	ADCY5	PDE10A	
FFAR4	ADAM17	TDP2	HPRT1	SLC1A2	CISD1	PPIA	
CACNA1B	ACE2	FLT3	NAALAD2	NEK2	WEE1	GSR	
PDE11A	IRAK4	HRH3	AMD1	CNR2	RET	CCND1	
GLS	ABL1	CACNA1H	P4HTM	IDH1	DYRK1A	PLK4	
C5AR1	EPHA2	TERT	SI	PDK1	GLO1	OR51E2	

S2-3: Common targets of SC compounds and disease

Merged common targets				
TTR	MTOR	PIK3CA	F2R	JAK2
PTPN11	TGFB1	PTGS2	ALK	GLB1
NFKB1	CASP3	EGFR	MAPK14	RELA
SLC2A1	CXCR4	BCHE	CNR1	BCL2
MAPK1	PIK3CG	NTRK1	NOS3	EZH2
NR1I2	CCR2	HSPA5	CDK4	RAF1
LGALS3	LMNA	MAP2K1	PTK2B	CXCR3
STAT1	NR3C2	BRAF	TERT	ABCG2
NFE2L2	HDAC9	GBA	F2	FGF2
PDGFRA	MMP2	MAPK8	BRD4	DNMT3B
TLR4	MMP13	EDNRA	ABCB1	PCNA
SERPINE1	MMP1	IL2	SIRT1	IL6

PRKAA1	MMP7	ESR1	PPARG	DNMT1
PLAU	HRAS	ELANE	TUBB3	NOS1
HIF1A	FHIT	STAT3	CXCR2	MDM2
MIF	AGTR1	ALOX5	SOAT1	MAPK3
CFTR	MMP9	IGF1R	CCND1	ACE
HSP90AA1	PARP1	RET	ERBB2	TNF
PDGFRB	IGFBP3	INSR	ADRB2	PIK3CB
NOS2	MMP3	TEK	AKT1	EP300
PTGS1	ADAM17	MET	GSTP1	
CASP8	SRC	AR	NT5E	
CYP2A6	KDR	NR3C1	CREBBP	
PIK3CD	FGFR1	SHH	TGM2	

S2-4: KEGG pathway enrichment analysis

KEGG Pathway	Count	PValue	Enrichment
Bladder cancer	15	3.2E-17	28.5498
Prostate cancer	30	4.3E-33	24.1349
EGFR tyrosine kinase inhibitor resistance	24	5.8E-26	23.7072
Central carbon metabolism in cancer	20	6.8E-21	22.296
Melanoma	20	1.2E-20	21.6767
Prolactin signaling pathway	19	2.3E-19	21.1812
Non-small cell lung cancer	19	4E-19	20.5928
Pancreatic cancer	20	3.8E-20	20.5358
AGE-RAGE signaling pathway in diabetic complications	25	7.9E-25	19.509
Renal cell carcinoma	17	1.6E-16	19.2263
Endocrine resistance	24	1.4E-23	19.1109
Thyroid cancer	9	1.5E-08	18.9817
Glioma	18	2.5E-17	18.7286
HIF-1 signaling pathway	26	2.6E-25	18.6141
VEGF signaling pathway	14	2.7E-13	18.517
PD-L1 expression and PD-1 checkpoint pathway in cancer	21	3.7E-20	18.413
Endometrial cancer	13	5.2E-12	17.4908
Acute myeloid leukemia	15	6.8E-14	17.4708
Chronic myeloid leukemia	17	8.3E-16	17.4554
C-type lectin receptor signaling pathway	21	1E-18	15.7573
Colorectal cancer	17	6.9E-15	15.4257
Type II diabetes mellitus	9	9.1E-08	15.2679
Fc epsilon RI signaling pathway	13	3.8E-11	14.9187
Aldosterone-regulated sodium reabsorption	7	6.2E-06	14.7636
Chagas disease	19	3.2E-16	14.5361

Hepatitis B	30	4.9E-26	14.4511
FoxO signaling pathway	24	1.7E-20	14.2967
Relaxin signaling pathway	23	2.5E-19	13.9134
ErbB signaling pathway	15	2.2E-12	13.7711
Toxoplasmosis	19	1.6E-15	13.3575
IL-17 signaling pathway	16	5.5E-13	13.2827
Antifolate resistance	5	0.00052	13.006
Proteoglycans in cancer	34	4.1E-28	12.9426
Platinum drug resistance	12	1.5E-09	12.8278
Longevity regulating pathway - multiple species	10	6.2E-08	12.7928
TNF signaling pathway	18	4.4E-14	12.3215
Adipocytokine signaling pathway	11	1.5E-08	12.2628
Epithelial cell signaling in Helicobacter pylori infection	11	1.5E-08	12.2628
Thyroid hormone signaling pathway	19	7.7E-15	12.2536
Leishmaniasis	12	2.8E-09	12.1615
Choline metabolism in cancer	15	1.6E-11	11.9443
Growth hormone synthesis, secretion and action	18	1.1E-13	11.7054
Kaposi sarcoma-associated herpesvirus infection	29	1.9E-22	11.6652
Toll-like receptor signaling pathway	16	4.5E-12	11.5609
Insulin resistance	16	4.5E-12	11.5609
Longevity regulating pathway	13	1E-09	11.3985
Pertussis	11	3.4E-08	11.2947
Neurotrophin signaling pathway	17	1.4E-12	11.148
Phospholipase D signaling pathway	21	1.5E-15	11.0727
Small cell lung cancer	13	1.5E-09	11.0268
Gastric cancer	21	1.7E-15	10.9984
GnRH secretion	9	1.3E-06	10.9738
T cell receptor signaling pathway	17	1.8E-12	10.9637
Apoptosis	19	6.3E-14	10.9021
Th17 cell differentiation	15	6.3E-11	10.8383
Inflammatory bowel disease	9	1.4E-06	10.805
Regulation of lipolysis in adipocytes	8	7.8E-06	10.7636
Fluid shear stress and atherosclerosis	19	9.3E-14	10.6668
Arginine biosynthesis	3	0.03129	10.6413
Lipid and atherosclerosis	29	3.4E-21	10.5258
Cellular senescence	21	4.2E-15	10.5049
Long-term depression	8	9.8E-06	10.4048
Sphingolipid signaling pathway	16	2.4E-11	10.3188
B cell receptor signaling pathway	11	9.1E-08	10.219
Breast cancer	19	2.5E-13	10.0863
Progesterone-mediated oocyte maturation	13	5E-09	9.94577
Hepatitis C	20	7.1E-14	9.87798
Legionellosis	7	7.1E-05	9.7545

Apoptosis - multiple species	4	0.00756	9.7545
Human cytomegalovirus infection	28	1.7E-19	9.71115
Estrogen signaling pathway	17	1.3E-11	9.6833
Yersinia infection	17	1.3E-11	9.6833
Measles	17	1.4E-11	9.61313
JAK-STAT signaling pathway	20	1.8E-13	9.40193
Long-term potentiation	8	2E-05	9.31774
Hepatocellular carcinoma	20	2.2E-13	9.29
Adherens junction	11	2.4E-07	9.23007
GnRH signaling pathway	11	2.4E-07	9.23007
Influenza A	20	3.1E-13	9.12702
Tuberculosis	21	6.8E-14	9.1042
Chemokine signaling pathway	22	2.1E-14	8.94163
Rap1 signaling pathway	24	1E-15	8.9184
Gap junction	10	1.5E-06	8.86773
Pathways in cancer	59	9.9E-42	8.67067
Osteoclast differentiation	15	1.3E-09	8.67067
Chemical carcinogenesis - receptor activation	23	1.4E-14	8.46617
p53 signaling pathway	8	3.9E-05	8.43633
Apelin signaling pathway	15	1.9E-09	8.42115
Amoebiasis	11	5.8E-07	8.41565
PI3K-Akt signaling pathway	38	1.8E-24	8.26008
Signaling pathways regulating pluripotency of stem cells	15	2.8E-09	8.1856
Platelet activation	13	4.7E-08	8.1812
Focal adhesion	21	6.8E-13	8.07269
Non-alcoholic fatty liver disease	16	8.5E-10	8.05533
Natural killer cell mediated cytotoxicity	13	6.1E-08	7.98794
Insulin signaling pathway	14	1.6E-08	7.97449
Ras signaling pathway	24	1.3E-14	7.93587
Malaria	5	0.00362	7.8036
AMPK signaling pathway	12	3.3E-07	7.73911
Human T-cell leukemia virus 1 infection	22	3.8E-13	7.7333
Human immunodeficiency virus 1 infection	21	1.5E-12	7.72998
Th1 and Th2 cell differentiation	9	2E-05	7.63396
Cholinergic synapse	11	1.5E-06	7.59643
MicroRNAs in cancer	30	6.5E-18	7.55187
mTOR signaling pathway	15	8.7E-09	7.50347
Efferocytosis	15	8.7E-09	7.50347
Coronavirus disease - COVID-19	22	9.7E-13	7.36821
Alcoholic liver disease	13	2.1E-07	7.14414
Autophagy - animal	15	1.8E-08	7.09419
MAPK signaling pathway	27	3.4E-15	6.99991

Chemical carcinogenesis - reactive oxygen species	20	3.6E-11	6.99875
Epstein-Barr virus infection	18	5.2E-10	6.95371
Diabetic cardiomyopathy	18	5.6E-10	6.91945
Salmonella infection	22	3.6E-12	6.89475
Viral carcinogenesis	18	6.1E-10	6.88553
Serotonergic synapse	10	1.4E-05	6.78574
Carbohydrate digestion and absorption	4	0.02156	6.64136
Parathyroid hormone synthesis, secretion and action	9	5.6E-05	6.6257
RIG-I-like receptor signaling pathway	6	0.00202	6.59459
Neutrophil extracellular trap formation	16	1.5E-08	6.53705
Regulation of actin cytoskeleton	19	4.7E-10	6.47461
Fc gamma R-mediated phagocytosis	8	0.00022	6.43596
Intestinal immune network for IgA production	4	0.02406	6.37029
Pathogenic Escherichia coli infection	16	2.5E-08	6.30594
cAMP signaling pathway	18	2.8E-09	6.24288
Ovarian steroidogenesis	4	0.0267	6.12047
Leukocyte transendothelial migration	9	0.0001	6.10717
Oxytocin signaling pathway	12	3.7E-06	6.08073
NF-kappa B signaling pathway	8	0.00034	6.00277
Human papillomavirus infection	25	2.2E-12	5.89396
Rheumatoid arthritis	7	0.00114	5.87368
Calcium signaling pathway	19	2.4E-09	5.86041
Hypertrophic cardiomyopathy	7	0.00142	5.63147
Inflammatory mediator regulation of TRP channels	7	0.00149	5.574
Graft-versus-host disease	3	0.09918	5.574
Viral protein interaction with cytokine and cytokine receptor	7	0.00166	5.46252
NOD-like receptor signaling pathway	13	3.8E-06	5.45413
Melanogenesis	7	0.00174	5.40844
Shigellosis	17	7.5E-08	5.3709
Transcriptional misregulation in cancer	13	5.5E-06	5.25631
cGMP-PKG signaling pathway	11	4.6E-05	5.17106
Axon guidance	12	1.8E-05	5.14523
Bacterial invasion of epithelial cells	5	0.01639	5.06728
Alzheimer disease	24	3.3E-10	4.87725
Necroptosis	10	0.00019	4.87725
Cytosolic DNA-sensing pathway	5	0.02102	4.70097
Viral myocarditis	4	0.05318	4.65887
Renin secretion	4	0.05713	4.52383
Glucagon signaling pathway	6	0.0115	4.37585
TGF-beta signaling pathway	6	0.01194	4.33534
Inositol phosphate metabolism	4	0.06543	4.27595
Vascular smooth muscle contraction	7	0.00706	4.07651

Adrenergic signaling in cardiomyocytes	8	0.00338	4.05382
Cushing syndrome	8	0.0035	4.02767
Mitophagy - animal	5	0.0418	3.78816
Tight junction	8	0.0058	3.67228
Complement and coagulation cascades	4	0.09592	3.62958
Oocyte meiosis	6	0.02541	3.57417
Cell cycle	7	0.01471	3.47931
Pathways of neurodegeneration - multiple diseases	21	1.8E-06	3.44277
Prion disease	12	0.00065	3.44277
Spinocerebellar ataxia	6	0.03523	3.27424
Retrograde endocannabinoid signaling	6	0.03993	3.16362
Alcoholism	7	0.03227	2.9056
Wnt signaling pathway	6	0.07031	2.6909
Endocytosis	8	0.03962	2.49715
Herpes simplex virus 1 infection	16	0.00197	2.43863
Thermogenesis	7	0.0746	2.35454
Huntington disease	9	0.04056	2.29518
Cytokine-cytokine receptor interaction	8	0.08337	2.10198
Amyotrophic lateral sclerosis	9	0.09117	1.92946

Supplementary file 3

S3-1: Disease hub targets network in Cytoscape

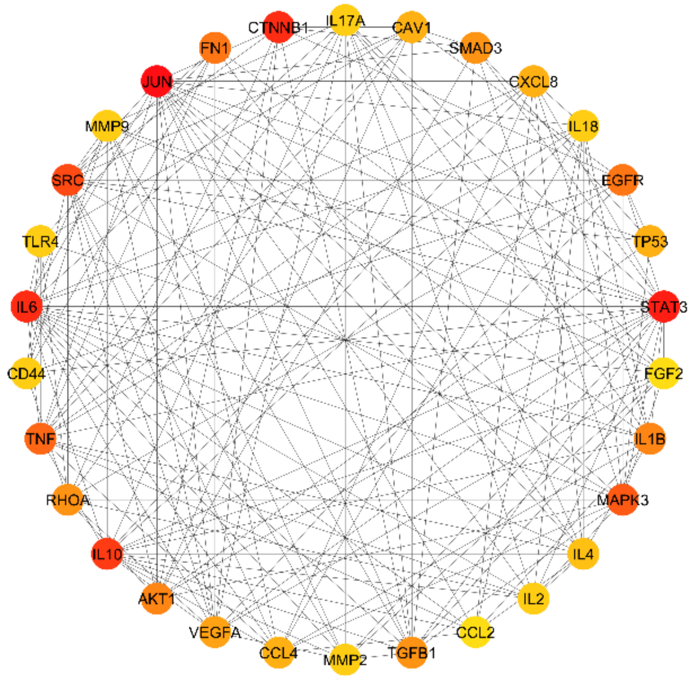

S3-2: Swertia chirayita components hub target screening by

a. **Degree targets**

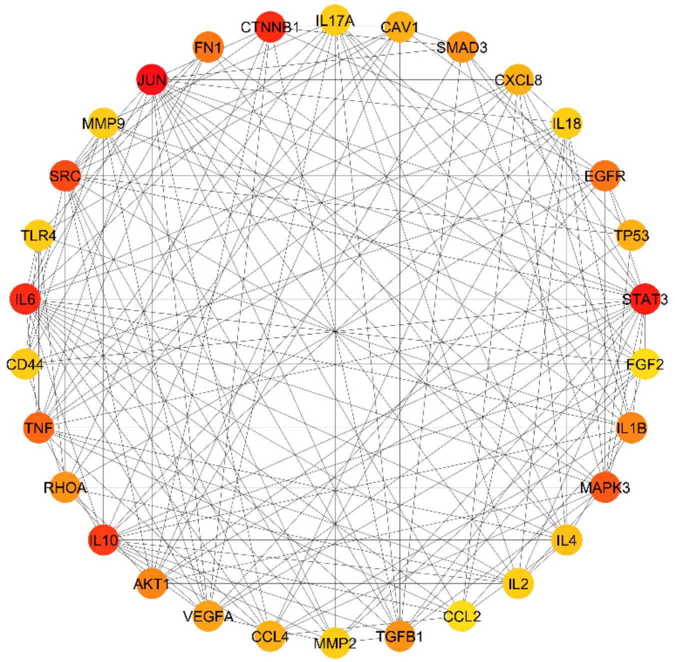

b. Closeness targets

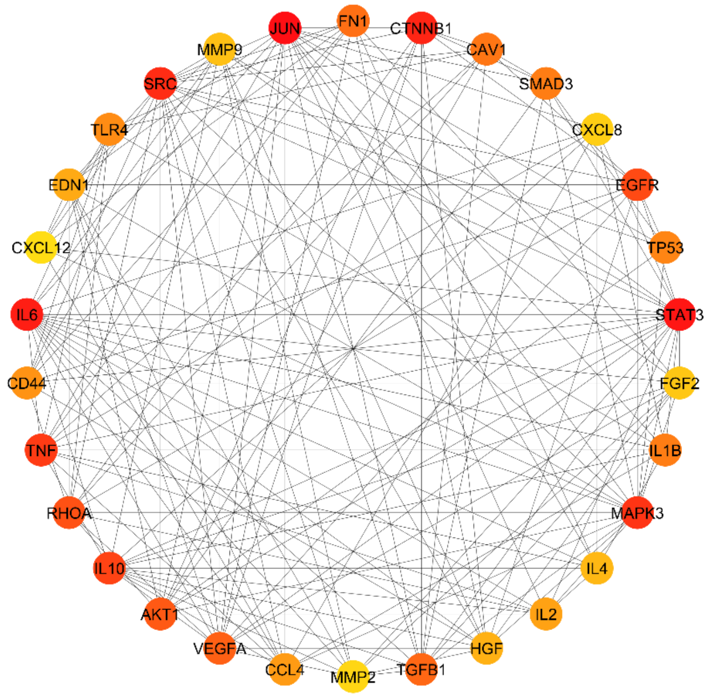

c. Betweenness targets

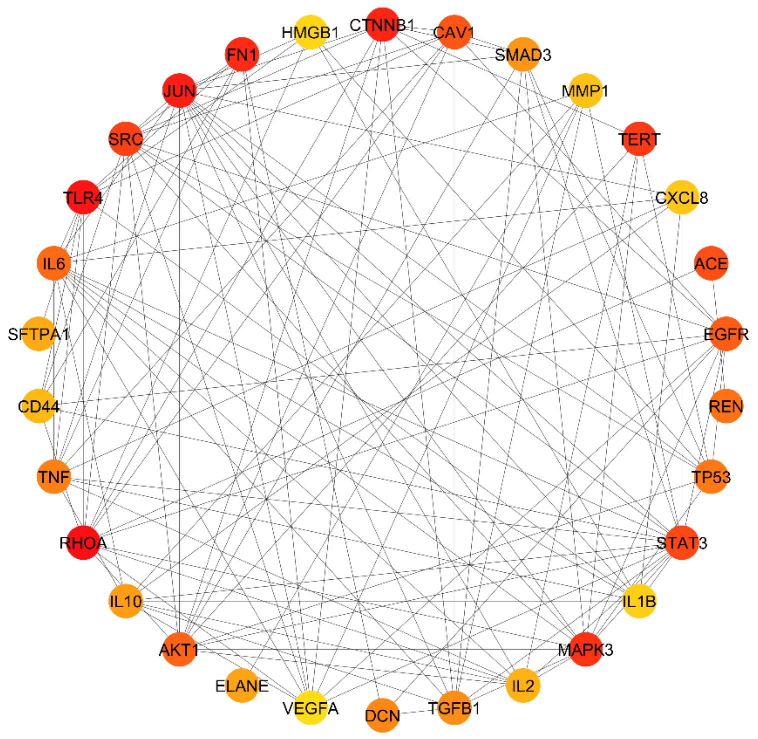

S3-3: Induced fit docking interaction matrix

3.1 BELDF-MMP9

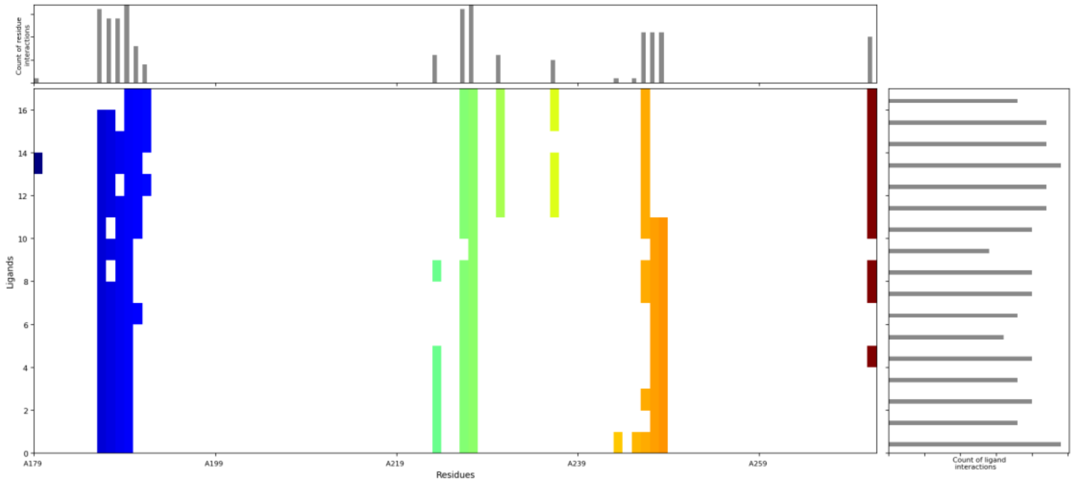

3.2 BELDF-TNF

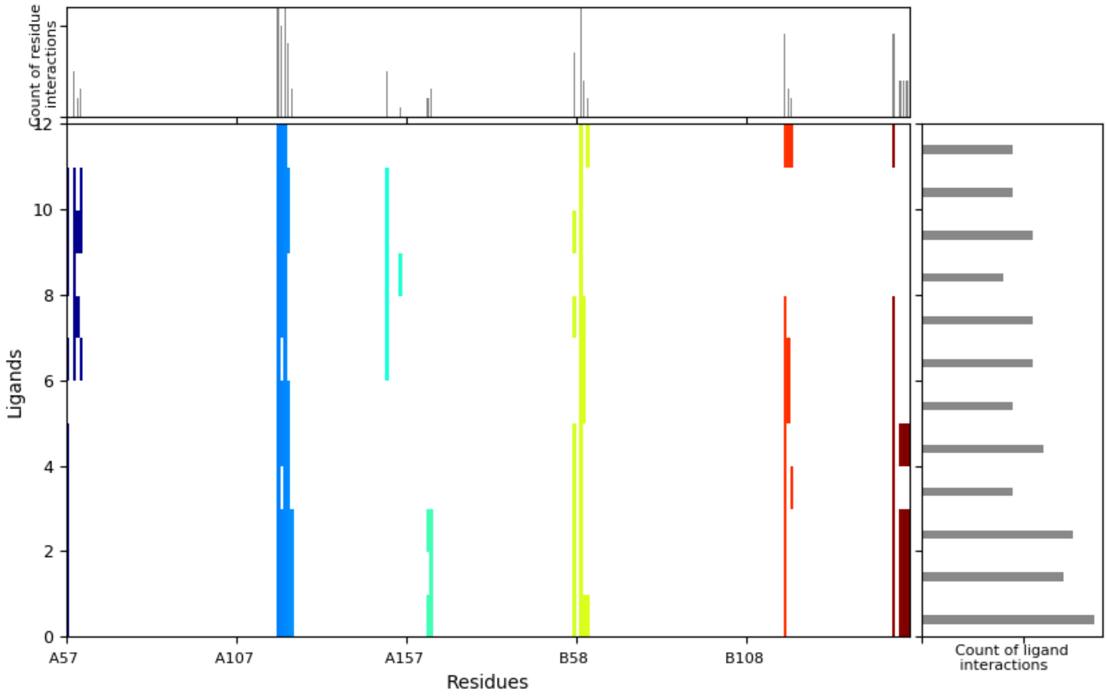

3.3 BELDF-MAPK3

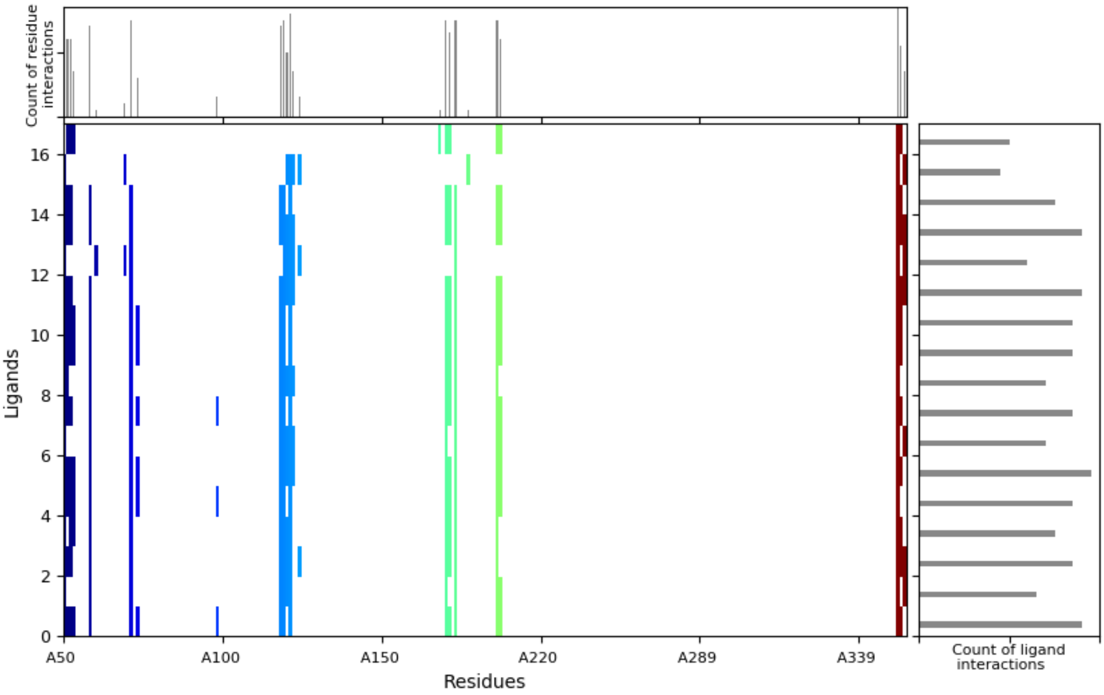

3.4 BELDF-EGFR

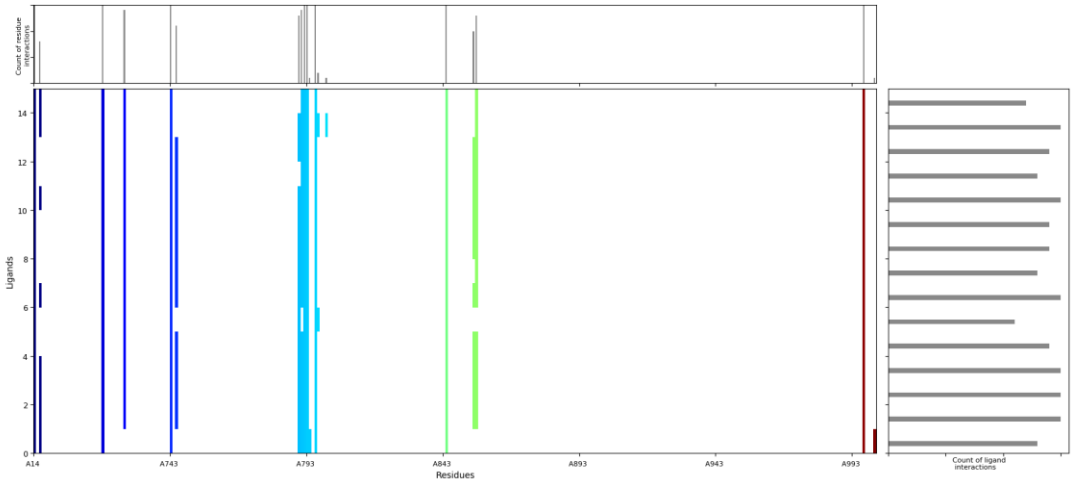

3.5 MANG-MMP9

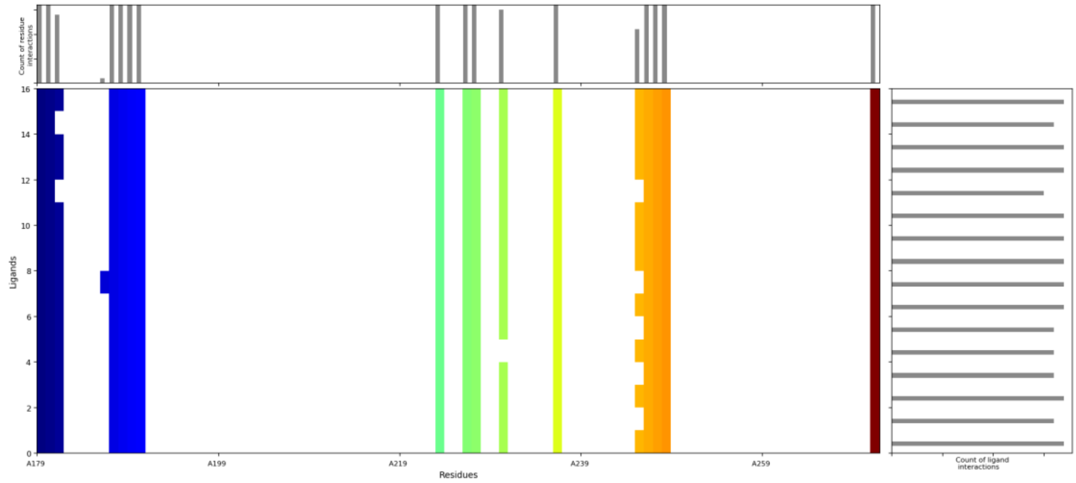

3.6 GENPD-MMP9

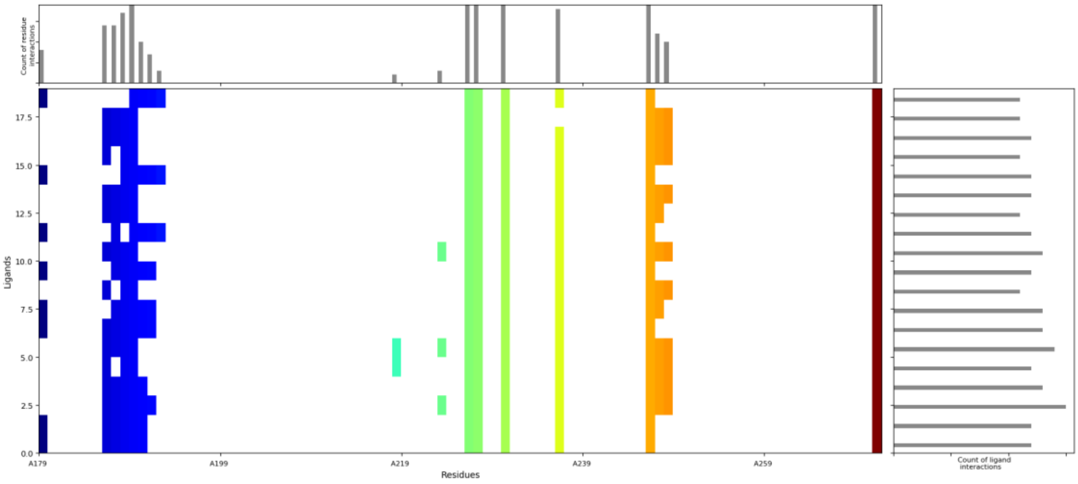

3.7 MANG-TNF

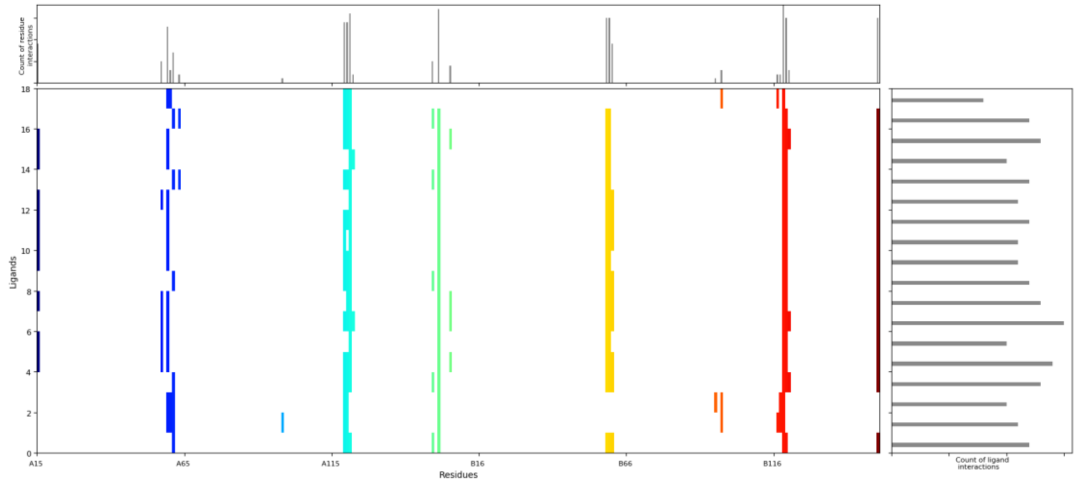

3.8 GENPD-TNF

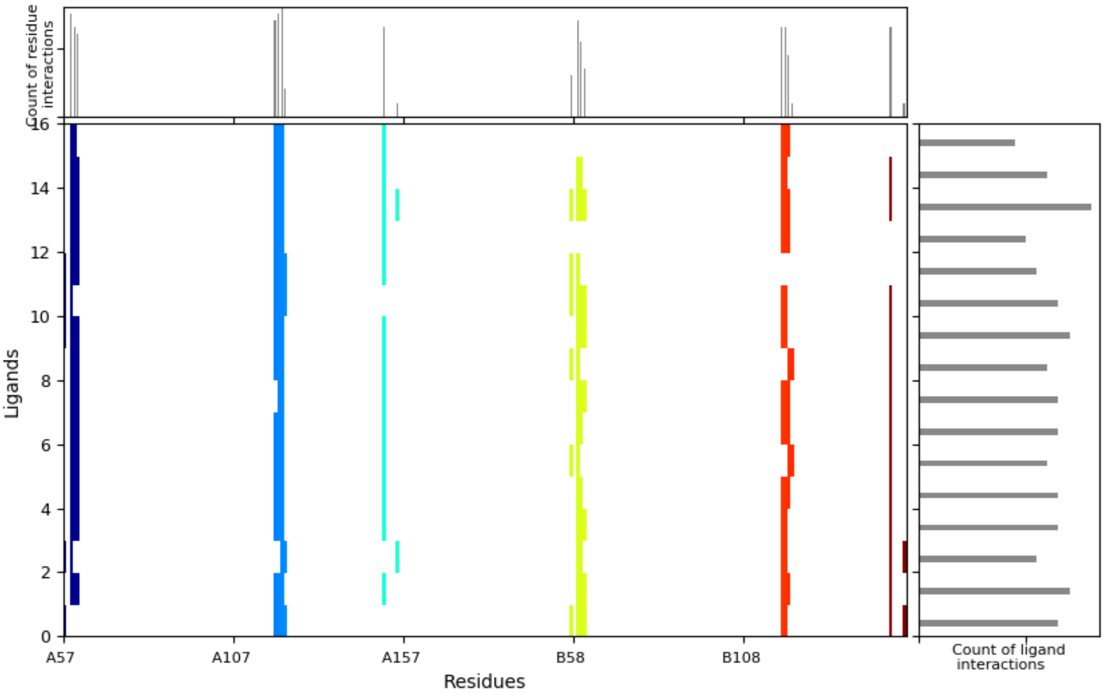

3.9 GENPD-EGFR

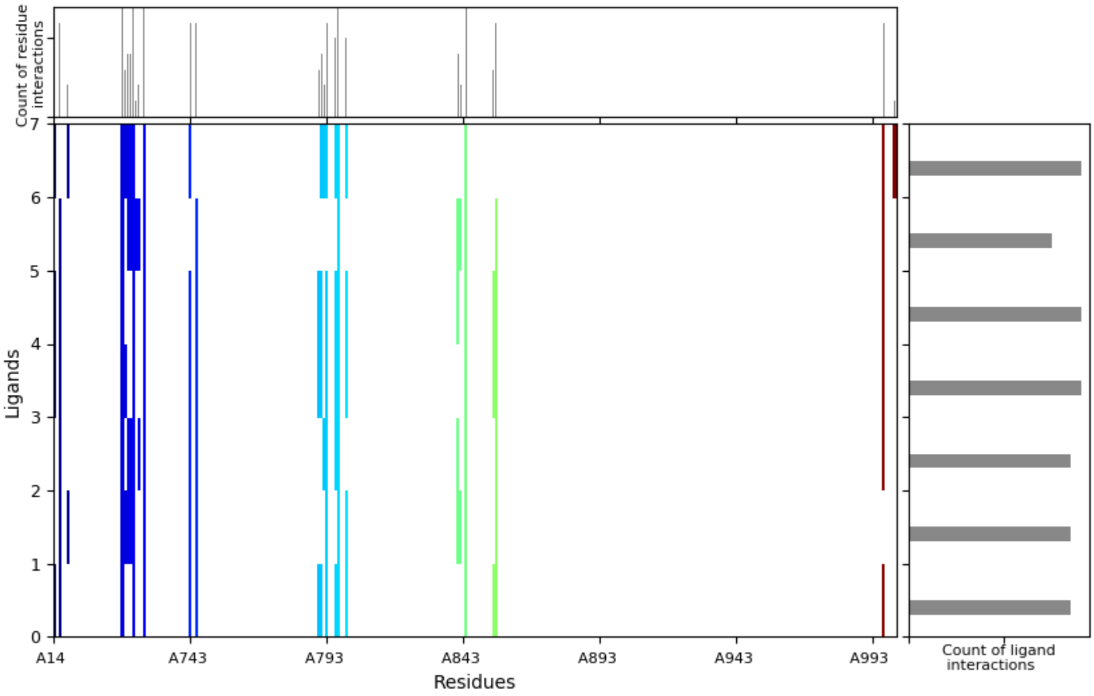

3.10 MANG-AKT1

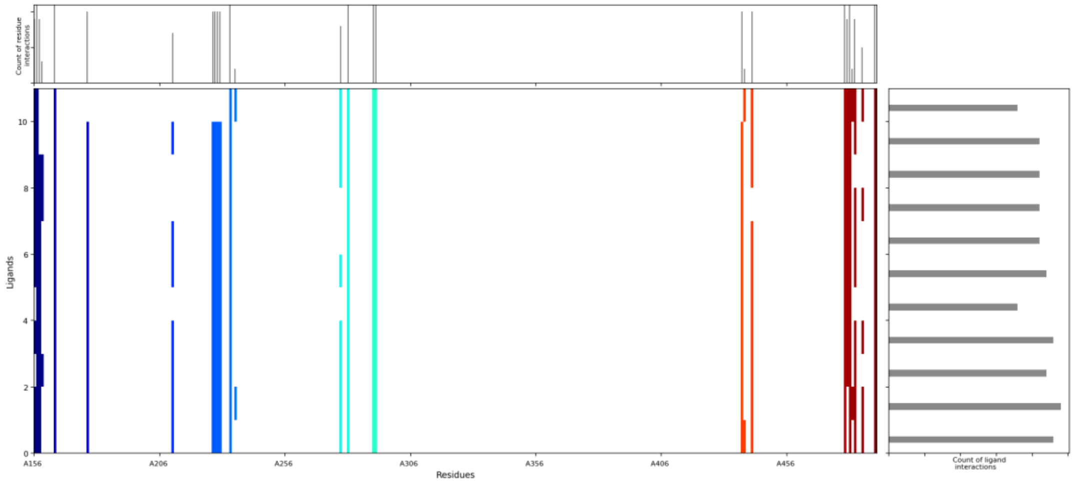

3.11 MANG-MAPK3

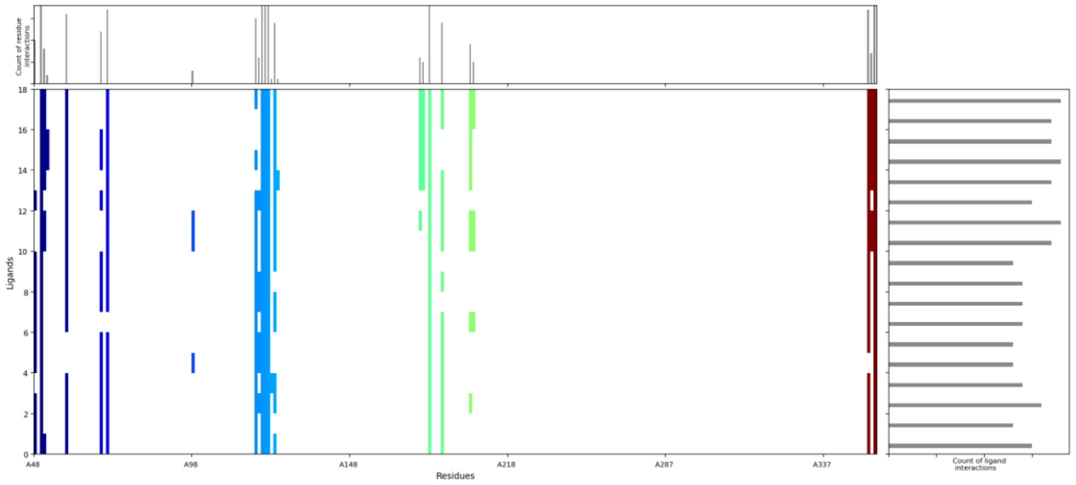

S3-4: Solvent Accessible Surface Area (SASA) and radius of Gyration (rGyr)

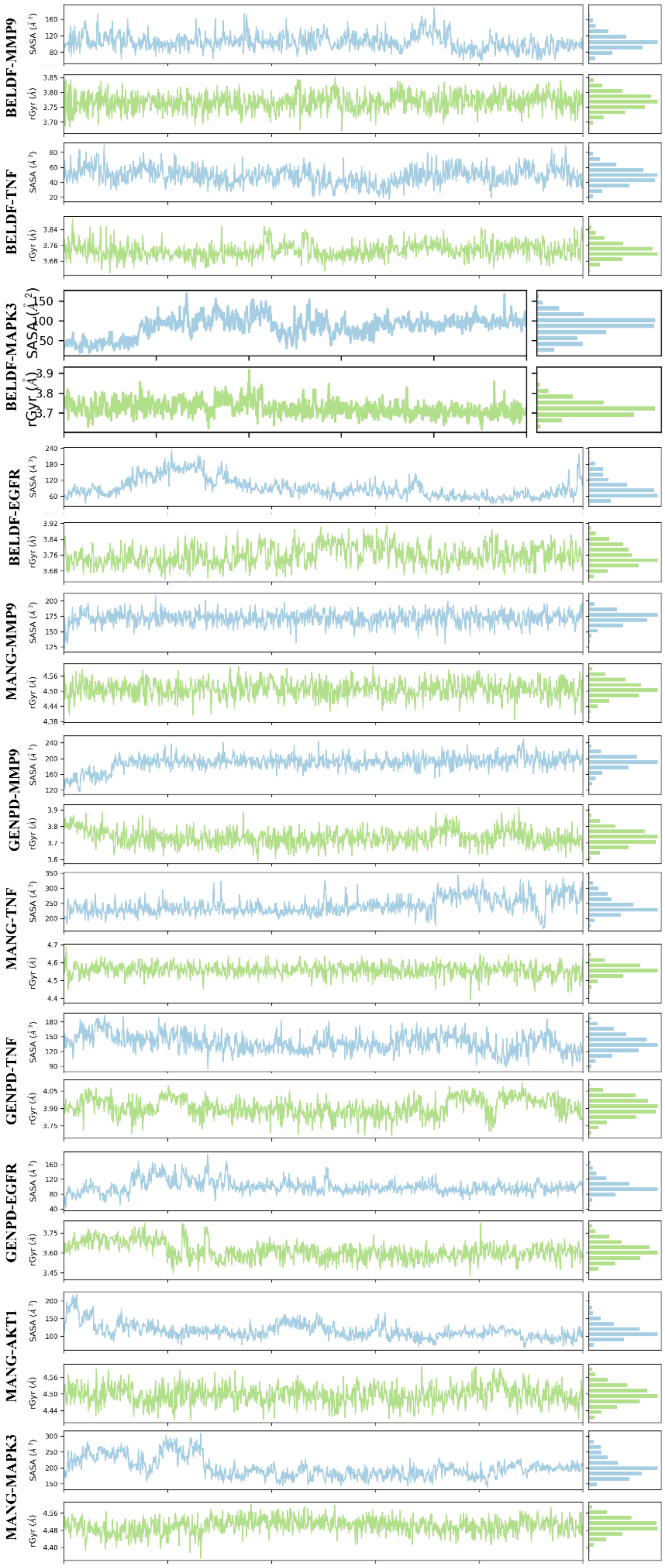

S3-5: RMSF, protein-ligand contact histogram, protein-ligand contact timeline, and 2D interaction images of MD simulations.

5.1 MMP9-BELDF

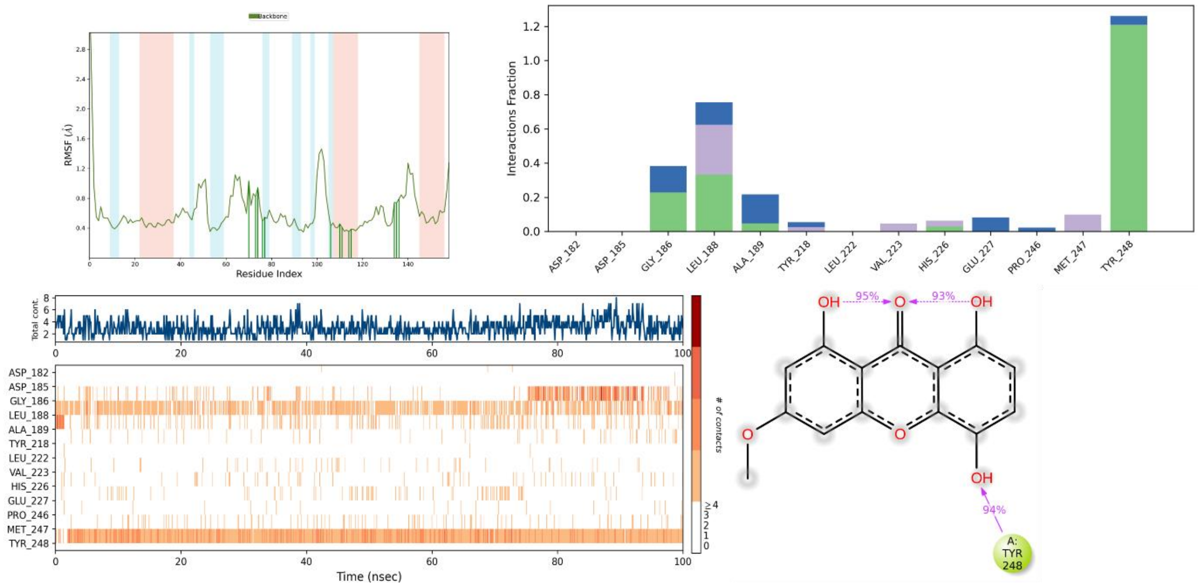

5.2 TNF-BELDF

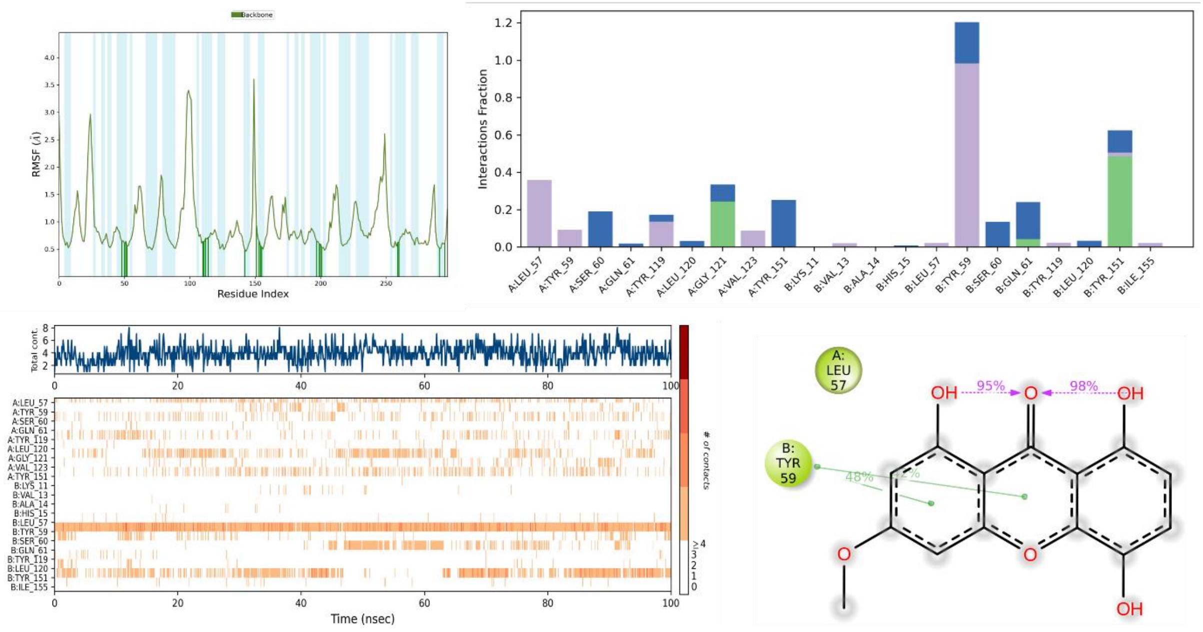

5.3 MAPK3-BELDF

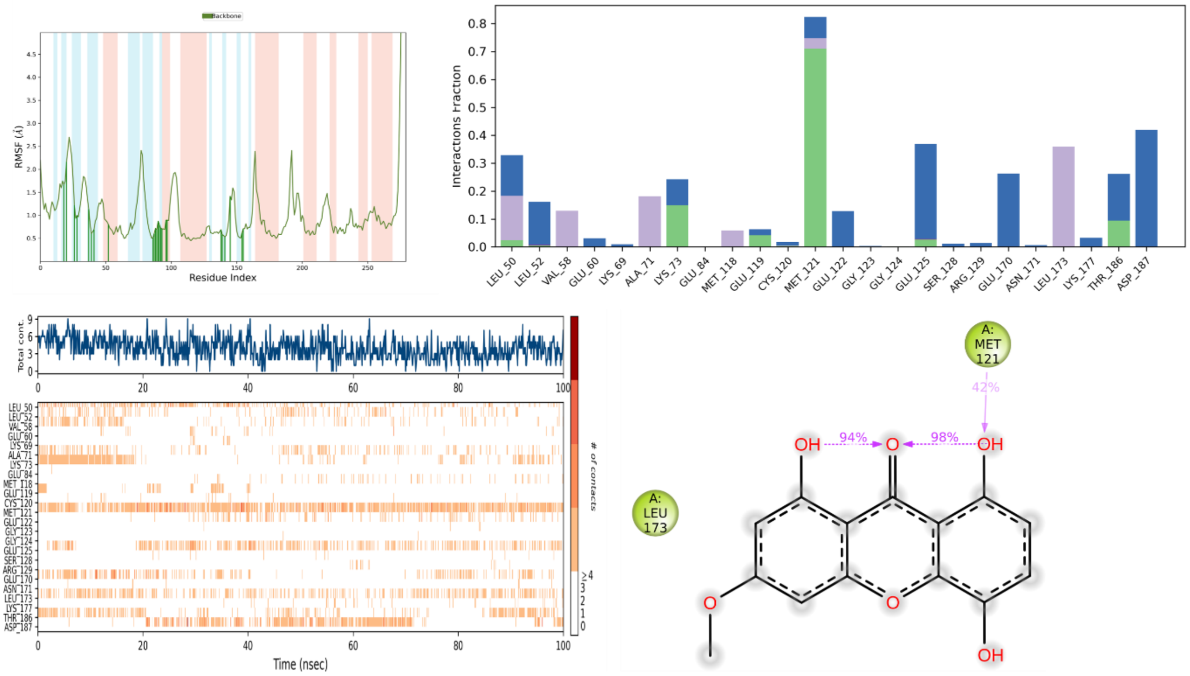

5.4 EGFR-BELDF

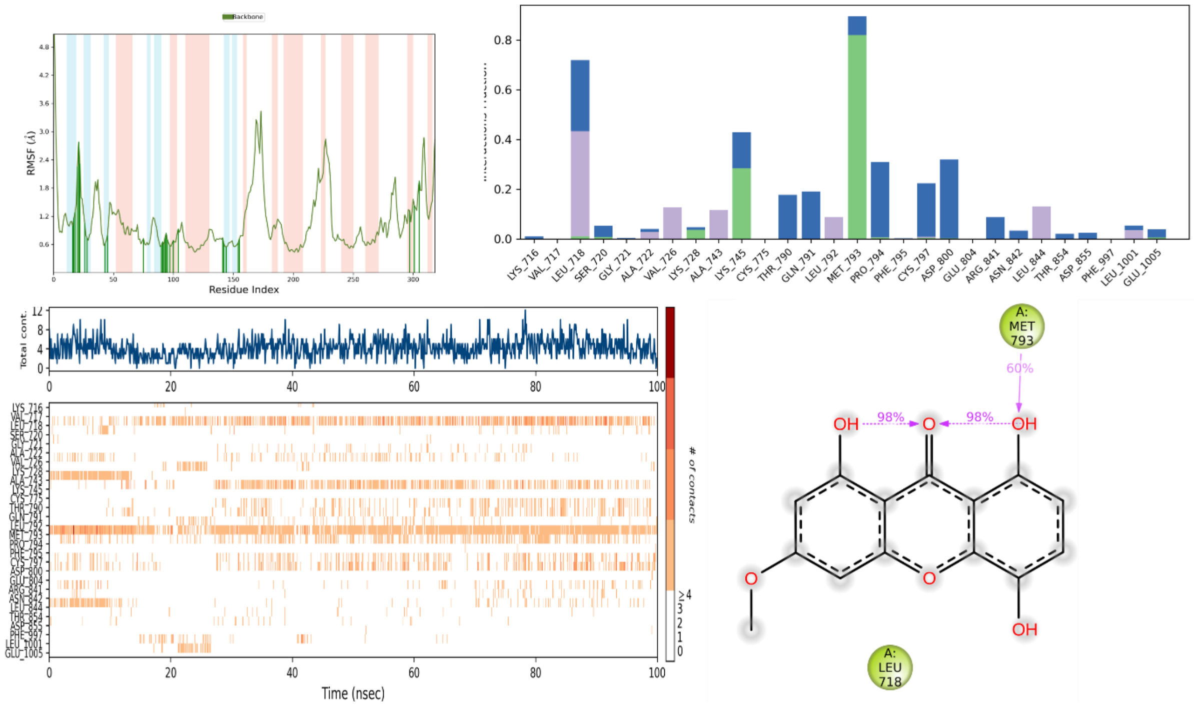

5.5 MMP9-MANG

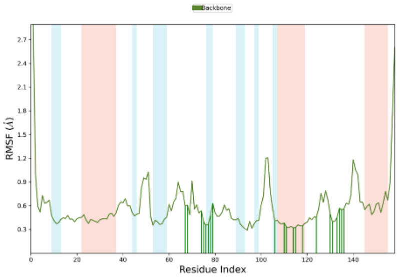

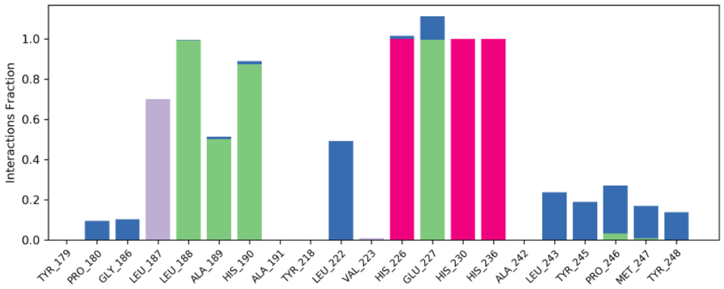

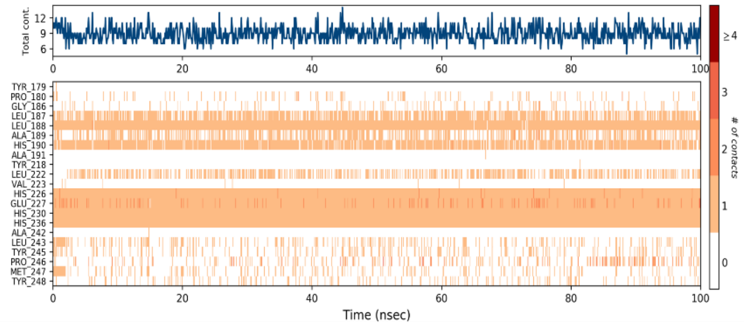

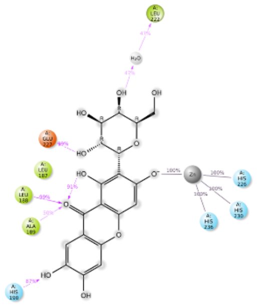

5.6 MMP9-GENPD

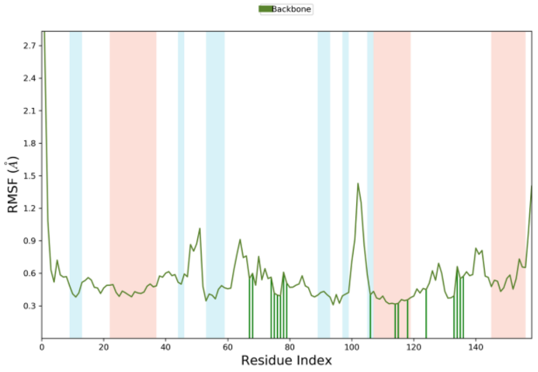

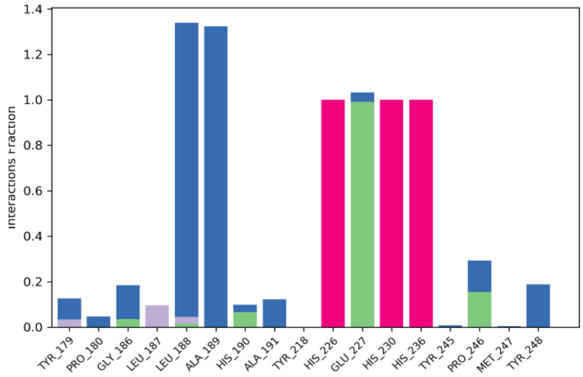

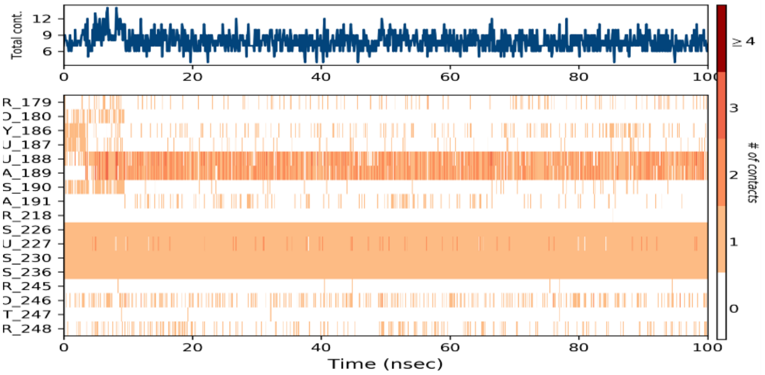

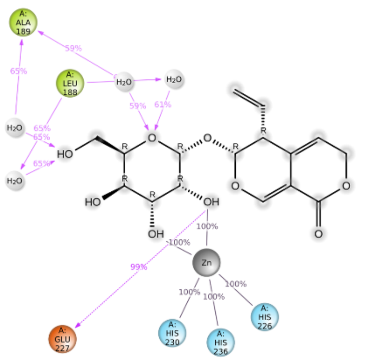

5.7 TNF-MANG

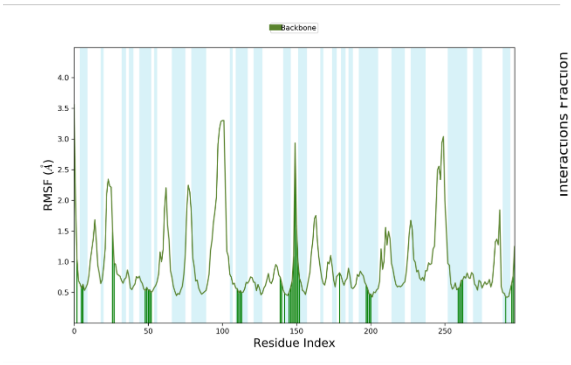

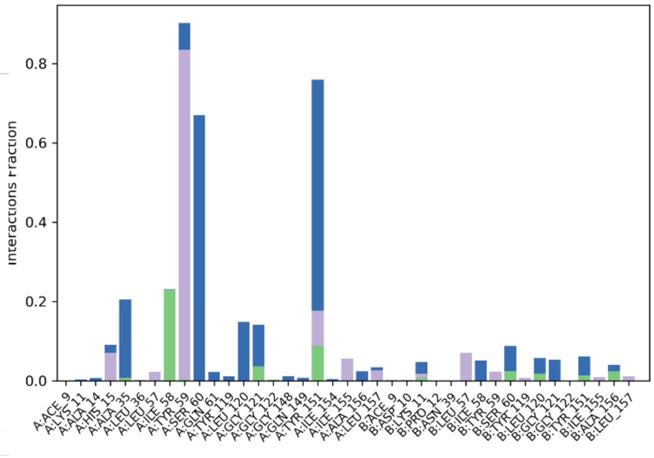

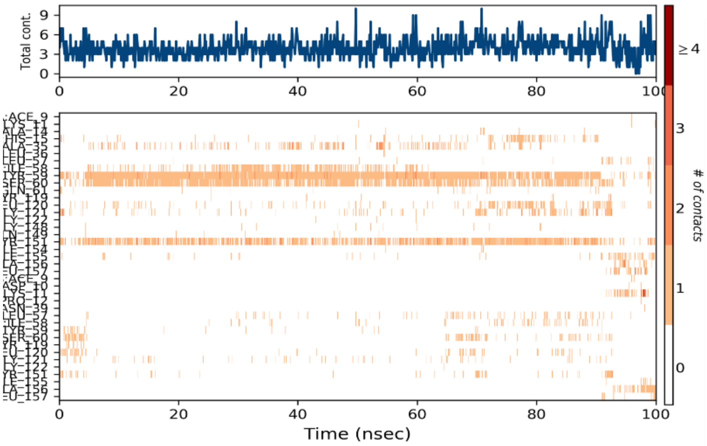

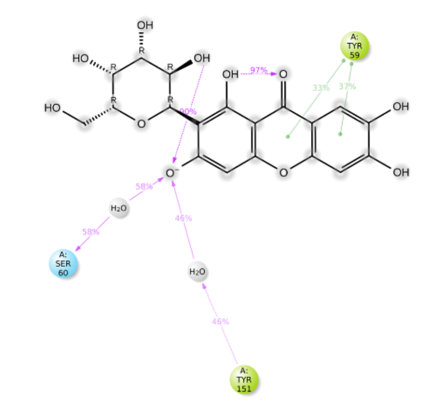

5.8 TNF-GENPD

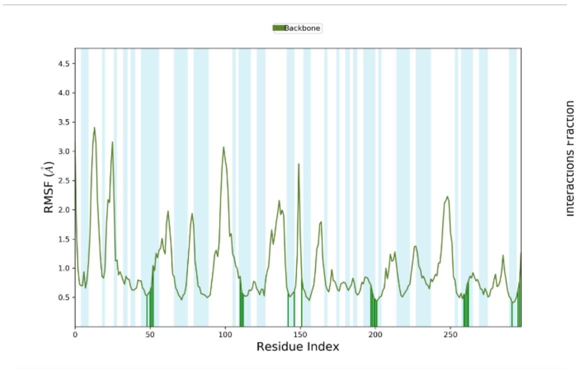

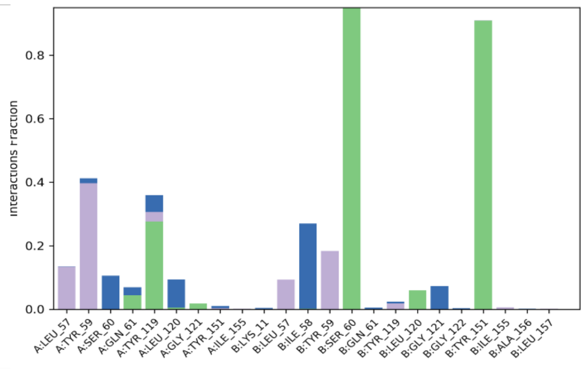

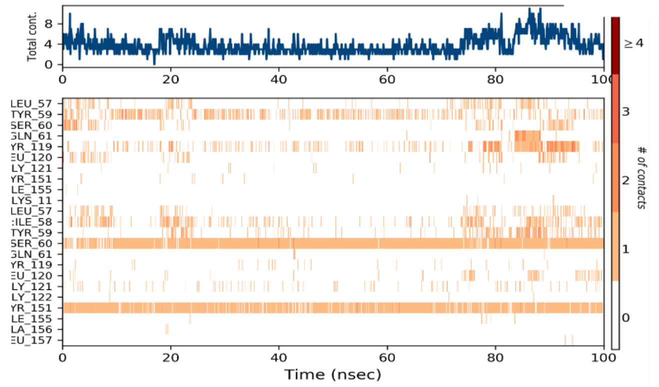

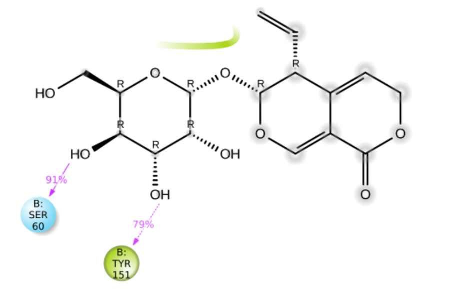

5.9 AKT1-MANG

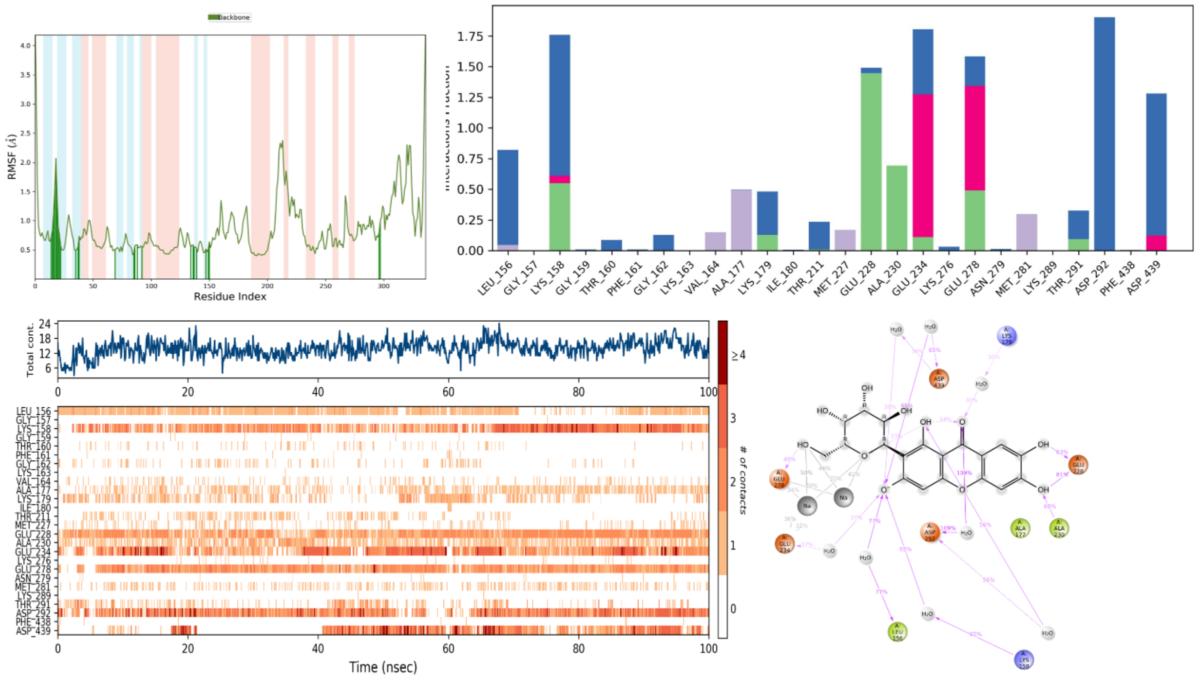

5.10 MAPK3-MANG

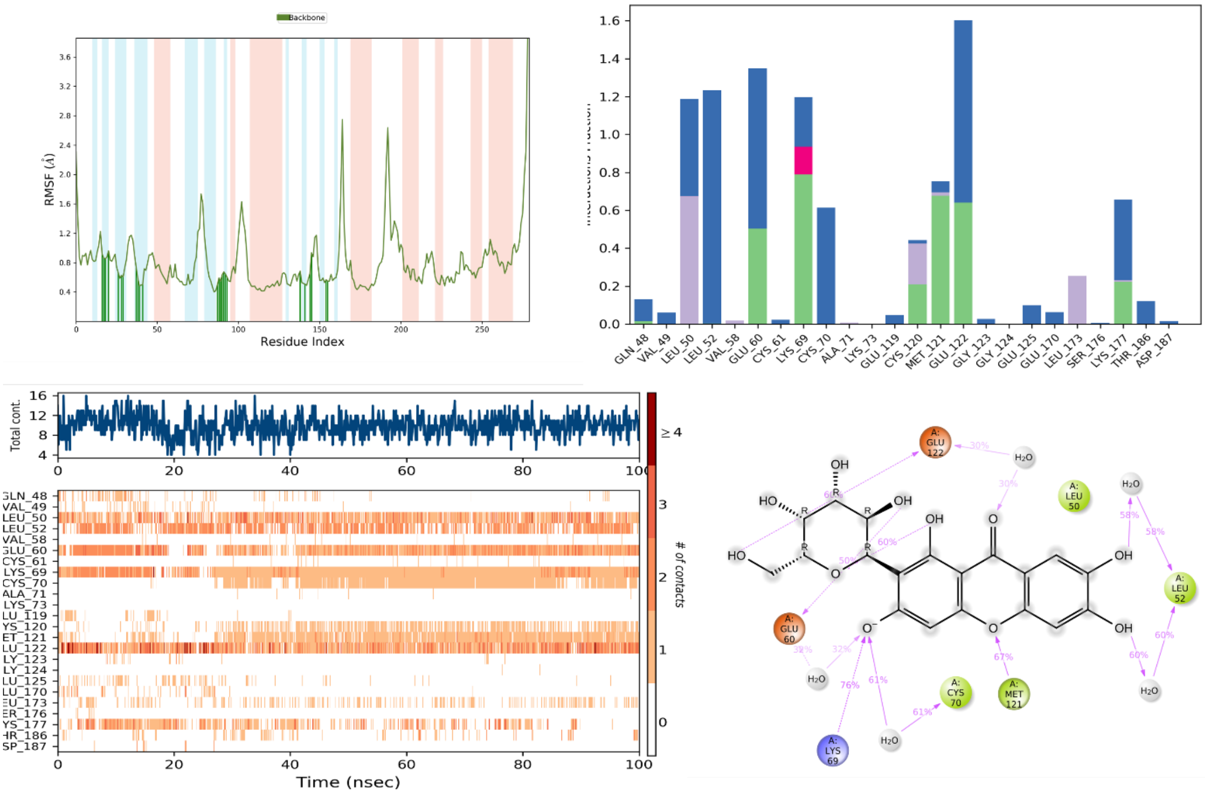

5.11 EGFR-GENPD

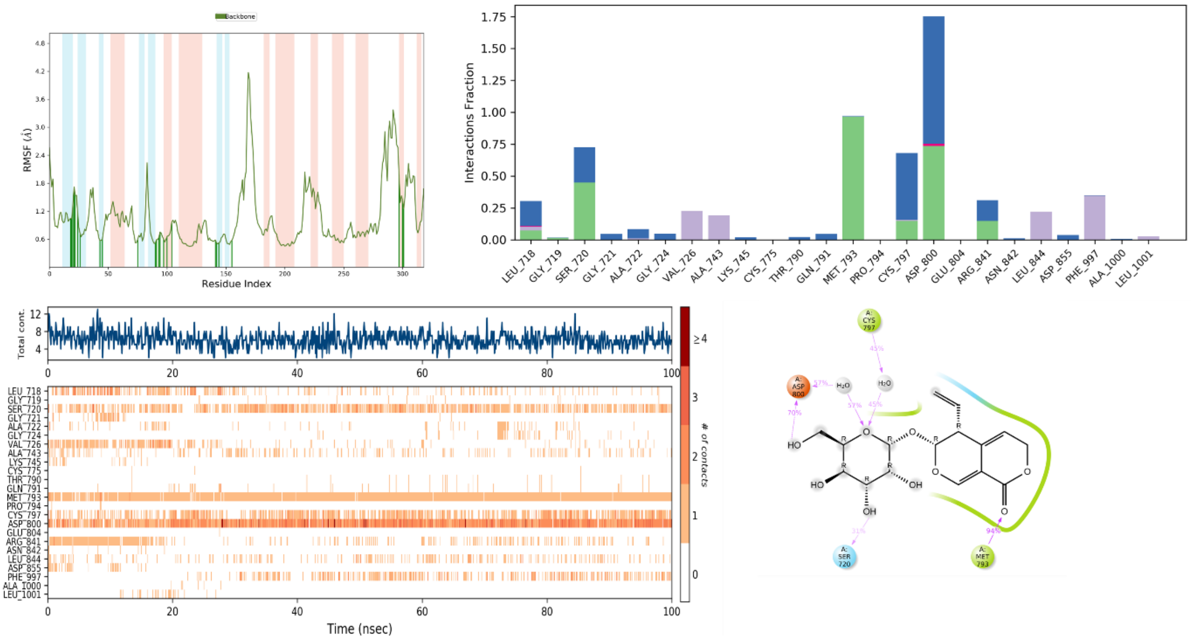

Supplementary file 4

S4-1: 1ALU XP-Docking

ligand	GScore	DockScore	LipophilicEvdW	HBond	Electro	Sitemap	LowMW	ExposPenal	EpikStatePenalty
MANG	-6.13	-6.13	-2.9	-2.69	-0.56	0	-0.1	0.12	0
SWNIN	-5.74	-5.66	-3.03	-1.91	-0.31	0	-0.5	0	0.08
NSWIN	-5.35	-5.35	-2.86	-1.93	-0.06	0	-0.5	0	0
BELDF	-4.97	-4.92	-2.78	-1.54	-0.15	0	-0.5	0	0.04
18DDXN	-4.96	-4.89	-2.7	-1.44	-0.32	0	-0.5	0	0.07
DMBEDF	-5.08	-4.68	-2.56	-1.92	-0.1	0	-0.5	0	0.4
SWCIN	-4.52	-4.48	-2.68	-1.29	-0.06	0	-0.5	0	0.04
ENCF	-4.47	-4.47	-2.59	-0.89	-0.49	0	-0.5	0	0
IBELDF	-4.6	-4.18	-2.55	-1.44	-0.11	0	-0.5	0	0.42
OLEA	-4.08	-4.08	-1.59	-1.25	-0.83	0	-0.5	0.09	0
DECU	-3.92	-3.9	-2.23	-0.96	-0.28	0	-0.49	0.04	0.03
DTPA	-3.85	-3.85	-1.15	-1.69	-0.19	-0.32	-0.5	0	0
SWIDE	-3.79	-3.79	-0.78	-2.09	-0.87	0	-0.31	0.25	0
CHIRT	-3.7	-3.67	-1.58	-1.38	-0.34	0	-0.5	0.1	0.03
SWPU	-3.52	-3.48	-1.1	-2.27	-0.23	0	0	0.09	0.04
DIMBELD	-3.35	-3.33	-1.56	-1.03	-0.39	0	-0.49	0.11	0.03
GENPD	-3.31	-3.31	-0.85	-1.52	-0.5	-0.29	-0.31	0.16	0
STEA	-3.21	-3.21	-2.94	0	0.01	0	-0.5	0.21	0
GENTN	-3.19	-3.19	-2.52	0	-0.17	0	-0.5	0	0
X9DDM	-3.53	-3.15	-2.26	-0.96	0.06	0	-0.5	0.13	0.39
PALA	-2.96	-2.96	-1.7	-0.8	-0.18	0	-0.5	0.22	0
ERTH	-2.3	-2.3	-1.01	-0.67	-0.75	0	-0.02	0.15	0
BSIT	-2.13	-2.13	-2.01	-0.14	-0.16	0	-0.12	0.29	0
SYRIN	-1.81	-1.81	-0.86	-0.83	-0.25	0	-0.11	0.23	0
16GBL	-1.69	-1.69	-0.89	-0.7	-0.31	0	-0.08	0.29	0
KAIRT	-1.48	-1.48	-1.76	0	0	0	-0.08	0.35	0
STIG	-1.34	-1.34	-1.66	-0.68	-0.28	0	-0.12	1.4	0
TARAX	-1.15	-1.15	-1.55	0	0.04	0	-0.08	0.45	0
LUPE	-1.1	-1.1	-0.59	-0.35	-0.26	0	-0.08	0.18	0
BAMY	-0.67	-0.67	-0.89	-0.7	-0.18	0	-0.08	1.18	0
PICA	-0.66	-0.66	-0.95	0	0.02	0	0	0.28	0
FRIED	-0.5	-0.5	-0.6	-0.7	-0.16	0	-0.08	1.04	0
SWONE	-0.41	-0.41	-1.01	0	0.02	0	-0.08	0.67	0
TARAXA	-0.19	-0.19	-1.62	0	0.01	0	0	1.42	0
SWOL	0.26	0.26	-0.61	0	-0.01	0	-0.08	0.96	0

URSA	0.3	0.3	-0.66	0	0.01	0	0	0.95	0
CALED	0.52	0.52	-0.87	0	0.11	0	-0.08	1.36	0
HOPB	0.74	0.74	-0.57	0	-0.15	0	-0.08	1.54	0
CHIRT	1.06	1.06	-0.7	0	0.01	0	-0.08	1.82	0

S4-2: 2AZ5 XP-Docking

ligand	GScore	DockScore	Lipophilic EvdW	PhobEn	HBond	Electro	Sitemap	LowMW	ExposPenal	EpikStatePenalty
SWIDE	-7.22	-7.22	-3	0	-2.88	-0.31	-0.72	-0.31	0	0
MANG	-7.02	-7.02	-3.38	-0.17	-3.22	-0.35	0	-0.1	0.2	0
GENPD	-6.45	-6.45	-3.87	-0.12	-1.92	-0.03	-0.19	-0.31	0	0
NSWIN	-6.31	-6.31	-3.74	-0.38	-1.39	-0.3	0	-0.5	0	0
BELDF	-6.26	-6.21	-2.8	0	-2.4	-0.59	0	-0.5	0.02	0.04
SWNIN	-6.22	-6.14	-3.42	0	-1.92	-0.38	0	-0.5	0	0.08
DMBEDF	-6.22	-5.82	-2.98	0	-2.4	-0.34	0	-0.5	0	0.4
CHIRT	-5.75	-5.72	-3.67	0	-1.31	-0.27	0	-0.5	0	0.03
SWCIN	-5.74	-5.7	-3.53	0	-1.44	-0.27	0	-0.5	0	0.04
DECU	-5.63	-5.6	-3.81	0	-0.96	-0.37	0	-0.49	0	0.03
DIMBELD	-5.61	-5.58	-4.05	0	-0.84	-0.24	0	-0.49	0	0.03
18DDXN	-5.48	-5.41	-3.35	0	-1.41	-0.23	0	-0.5	0	0.07
X9DDM	-5.51	-5.13	-3.68	0	-0.96	-0.37	0	-0.5	0	0.39
IBELDF	-5.5	-5.08	-3.32	0	-1.44	-0.24	0	-0.5	0	0.42
KAIRT	-4.98	-4.98	-5.27	-0.17	0	0.03	0	-0.08	0.51	0
STIG	-4.59	-4.59	-4.63	0	0	0.04	0	-0.12	0.12	0
OLNA	-4.51	-4.51	-4.14	-0.47	0	0	-0.08	0	0.17	0
ENCF	-4.45	-4.45	-2.17	0	-1.24	-0.21	-0.33	-0.5	0	0
GENTN	-4.41	-4.41	-3.28	-0.23	0	-0.05	-0.35	-0.5	0	0
CALED	-4.38	-4.38	-4.28	-0.26	0	-0.03	0	-0.08	0.28	0
URSA	-4.32	-4.32	-4.33	-0.12	0	0.07	0	0	0.06	0
16GBL	-4.29	-4.29	-4.29	0	0	0.02	0	-0.08	0.05	0
SWOL	-4.27	-4.27	-4.32	0	0	0.04	0	-0.08	0.09	0
TARAX	-4.27	-4.27	-4.41	-0.48	0	0.02	0	-0.08	0.69	0
SWONE	-4.25	-4.25	-4.31	0	0	0.03	0	-0.08	0.12	0
BSIT	-4.25	-4.25	-4.17	-0.13	0	0.03	0	-0.12	0.14	0

PALA	-4.25	-4.25	-3.07	0	-0.35	-0.34	0	-0.5	0.01	0
CHIRT	-4.24	-4.24	-3.71	0	-0.49	-0.1	0	-0.08	0.13	0
PICA	-4.2	-4.2	-4.46	0	0	0.07	-0.01	0	0.2	0
BAMY	-4.14	-4.14	-4.01	-0.14	0	0.01	0	-0.08	0.09	0
TARAXA	-4.08	-4.08	-4.29	0	0	0	-0.22	0	0.43	0
DTPA	-4.05	-4.05	-1.99	0	-0.96	-0.01	-0.6	-0.5	0	0
LUPE	-4.05	-4.05	-4.18	0	0	0.03	0	-0.08	0.18	0
HOPB	-4.04	-4.04	-3.42	-0.23	-0.37	-0.12	0	-0.08	0.17	0
OLEA	-4.04	-4.04	-3.58	0	0	-0.01	-0.08	-0.5	0.13	0
FRIED	-4.01	-4.01	-3.78	0	0	0.02	-0.4	-0.08	0.23	0
STEA	-3.93	-3.93	-2.44	0	-0.9	-0.24	0	-0.5	0.16	0
ERTH	-3.79	-3.79	-3.38	0	-0.39	-0.08	0	-0.02	0.09	0
SYRIN	-2.71	-2.71	-2.46	0	-0.48	0.02	0	-0.11	0.31	0

S4-3: 3FHR XP-Docking

ligand	GScore	DockScore	LipophilicEvdW	HBond	Electro	Sitemap	LowMW	ExposPenal	EpikStatePenalty
MANG	-7.64	-7.64	-3.72	-3.29	-0.65	0	-0.1	0.13	0
SWCIN	-7.33	-7.29	-3.32	-2.86	-0.65	0	-0.5	0	0.04
DECU	-6.87	-6.84	-4.06	-2	-0.38	0	-0.49	0.07	0.03
18DDXN	-6.81	-6.74	-4.34	-1.74	-0.3	0	-0.5	0.09	0.07
DIMBELD	-6.67	-6.64	-3.26	-2.47	-0.63	0	-0.49	0.18	0.03
CHIRT	-6.56	-6.53	-3.77	-2.14	-0.41	0	-0.5	0.26	0.03
X9DDM	-6.81	-6.42	-3.76	-2.17	-0.45	0	-0.5	0.07	0.39
NSWIN	-6.42	-6.42	-3.52	-2.28	-0.32	0	-0.5	0.19	0
DMBEDF	-6	-5.6	-3.37	-2.33	-0.18	0	-0.5	0.38	0.4
BELDF	-5.33	-5.29	-2.32	-2.45	-0.58	0	-0.5	0.51	0.04
DTPA	-4.82	-4.82	-1.97	-1.34	-0.4	-0.61	-0.5	0	0
SWNIN	-4.84	-4.76	-2.13	-2.21	-0.34	0	-0.5	0.35	0.08
SWIDE	-4.5	-4.5	-2.25	-1.42	-0.26	-0.32	-0.31	0.04	0
GENPD	-4.47	-4.47	-1.82	-2.33	-0.12	-0.4	-0.31	0.51	0
AMARSW	-4.12	-3.95	-1.17	-2.46	-0.34	-0.28	0	0.13	0.17
GENTN	-3.86	-3.86	-2.7	-0.59	-0.06	0	-0.5	0	0
IBELDF	-4.05	-3.63	-2.56	-0.98	-0.15	0	-0.5	0.14	0.42
ENCF	-3.01	-3.01	-1.42	-1	-0.14	0	-0.5	0.05	0
STEA	-2.92	-2.92	-1.3	-1.05	-0.08	0	-0.5	0	0
OLEA	-2.82	-2.82	-2.99	-0.25	0.04	-0.13	-0.5	0.2	0
PALA	-2.65	-2.65	-2.32	0	0.01	0	-0.5	0.16	0

SYRIN	-2.62	-2.62	-2.32	-0.48	0.08	0	-0.11	0.21	0
TARAXA	-2.3	-2.3	-1.76	-0.51	-0.07	-0.26	0	0.29	0
KAIRT	-1.59	-1.59	-1.08	-1.21	-0.52	0	-0.08	1.3	0
SWOL	-1.54	-1.54	-1.59	0	0.07	0	-0.08	0.05	0
FRIED	-1.48	-1.48	-1.72	-0.14	-0.05	0	-0.08	0.51	0
BSIT	-1.4	-1.4	-1.57	0	0.07	0	-0.12	0.21	0
PICA	-0.88	-0.88	-1.26	0	-0.02	-0.35	0	0.75	0
OLNA	-0.78	-0.78	-1.16	0	0.08	0	0	0.31	0
TARAX	-0.35	-0.35	-1.48	0	0.07	0	-0.08	1.15	0
SWONE	0	0	-1.46	0	0.04	0	-0.08	0.5	0

S4-4: 3POZ XP-Docking

ligand	GScore	DockScore	LipophilicEvdW	HBond	Electro	Sitemap	LowMW	ExposPenal	EpikStatePenalty
MANG	-7.35	-7.35	-2.22	-4.02	-1.09	0	-0.1	0.07	0
SWNIN	-7.43	-7.35	-4.39	-2.42	-0.12	0	-0.5	0	0.08
GENPD	-7.26	-7.26	-2.36	-3.14	-1.02	-0.43	-0.31	0	0
DMBEDF	-7.65	-7.25	-3.97	-2.96	-0.22	0	-0.5	0	0.4
NSWIN	-7.2	-7.2	-4.09	-2.37	-0.24	0	-0.5	0	0
BELDF	-7.02	-6.98	-4.33	-2.17	-0.2	0	-0.5	0.18	0.04
IBELDF	-7.31	-6.9	-4.1	-2.49	-0.22	0	-0.5	0	0.42
GENTN	-6.83	-6.83	-3.19	-0.7	-0.08	-0.29	-0.5	0	0
DTPA	-6.82	-6.82	-2.47	-2.04	-0.54	-0.5	-0.5	0.15	0
DIMBELD	-6.75	-6.72	-4.61	-1.52	-0.12	0	-0.49	0	0.03
SWCIN	-6.76	-6.72	-3.9	-2.1	-0.25	0	-0.5	0	0.04
SWPU	-6.66	-6.62	-1.71	-4.25	-0.9	0	0	0.21	0.04
18DDXN	-6.59	-6.53	-4.61	-1.31	-0.17	0	-0.5	0	0.07
CHIRT	-6.54	-6.51	-3.85	-1.97	-0.38	0	-0.5	0.16	0.03
X9DDM	-6.8	-6.41	-4.42	-1.29	-0.39	0	-0.5	0	0.39
DECU	-6.34	-6.32	-4.83	-0.85	-0.17	0	-0.49	0	0.03
SWIDE	-5.44	-5.44	-1.81	-2.59	-0.45	-0.38	-0.31	0.09	0
STEA	-5	-5	-4.54	-0.18	0.1	0	-0.5	0.13	0
AMARG	-4.97	-4.79	-2.71	-1.92	-0.5	-0.11	0	0.28	0.17
PALA	-4.21	-4.21	-2.03	-1.22	-0.5	0	-0.5	0.04	0
SYRIN	-4.17	-4.17	-3.52	-0.48	-0.1	0	-0.11	0.04	0
AMARSW	-4.03	-3.85	-1.94	-2.15	-0.15	-0.02	0	0.24	0.17
FRIED	-3.8	-3.8	-3.82	0	-0.02	-0.05	-0.08	0.17	0

OLEA	-3.61	-3.61	-2.21	-0.9	-0.27	0	-0.5	0.27	0
ENCF	-3.44	-3.44	-1.09	-1.81	-0.28	-0.09	-0.5	0.32	0
SWONE	-3.38	-3.38	-3.52	0	0.01	0	-0.08	0.22	0
PICA	-3.13	-3.13	-3.16	0	-0.02	-0.25	0	0.3	0
CHIRT	-3.1	-3.1	-3.33	0	0.1	0	-0.08	0.21	0
STIG	-3.08	-3.08	-2.98	0	0.07	0	-0.12	0.14	0
SWOL	-3.06	-3.06	-3.18	0	0.01	0	-0.08	0.18	0
HOPB	-2.78	-2.78	-2.96	0	0.09	0	-0.08	0.17	0
OLNA	-2.73	-2.73	-3.3	0	0.24	0	0	0.33	0
ERTH	-2.53	-2.53	-3.04	0	0.14	0	-0.02	0.39	0
KAIRT	-2.5	-2.5	-3.02	0	0.13	0	-0.08	0.47	0
BAMY	-2.4	-2.4	-2.65	-0.28	0.18	0	-0.08	0.43	0
CALED	-2.4	-2.4	-2.49	0	0.09	0	-0.08	0.08	0
16GBL	-2.37	-2.37	-2.34	0	-0.16	0	-0.08	0.21	0
TARAX	-2.33	-2.33	-1.98	-0.45	-0.02	0	-0.08	0.2	0
LUPE	-2.27	-2.27	-1.91	-0.43	-0.14	0	-0.08	0.28	0
BSIT	-2.03	-2.03	-1.88	-0.25	-0.02	0	-0.12	0.24	0
URSA	-1.83	-1.83	-2.29	0	0.16	0	0	0.3	0

S4-5: 4GV1 XP-Docking

ligand	GScore	DockScore	Lipophilic EvdW	Phob En	HBond	Electro	Sitemap	Low MW	ExposPenal	EpikStatePenalty
SWNIN	-8.75	-8.68	-2.82	-0.3	-3.2	-0.93	0	-0.5	0	0.08
NSWIN	-8.4	-8.4	-2.85	-0.3	-3.15	-0.63	0	-0.5	0.02	0
MANG	-4.84	-4.84	-0.69	0	-3.66	-0.49	0	-0.1	0.1	0
DTPA	-4.74	-4.74	-2	0	-1.59	-0.4	-0.38	-0.5	0.14	0
BELDF	-4.68	-4.63	-2.04	0	-1.78	-0.36	0	-0.5	0	0.04
X9DDM	-4.63	-4.24	-2.37	-0.19	-1.22	-0.34	0	-0.5	0	0.39
DMBEDF	-4.64	-4.24	-3.22	-0.2	-1.44	0.18	0	-0.5	0.54	0.4
SWCIN	-4.28	-4.24	-2.27	0	-1.41	-0.09	0	-0.5	0	0.04
OLEA	-4.2	-4.2	-2.84	0	-0.35	-0.35	-0.17	-0.5	0	0
CHIRT	-3.9	-3.87	-2.29	0	-1.01	-0.1	0	-0.5	0	0.03
18DDXN	-3.87	-3.8	-2.37	-0.12	-0.73	-0.15	0	-0.5	0	0.07
DIMBELD	-3.81	-3.78	-2.18	0	-1.11	-0.02	0	-0.49	0	0.03
GENTN	-3.77	-3.77	-2.89	-0.09	0	0.01	-0.3	-0.5	0	0
DECU	-3.78	-3.75	-1.75	0	-1.23	-0.38	0	-0.49	0.08	0.03
IBELDF	-3.88	-3.46	-1.2	0	-1.8	-0.37	0	-0.5	0	0.42

GENPD	-3.25	-3.25	-0.98	0	-1.4	-0.73	-0.08	-0.31	0.25	0
ENCF	-3.24	-3.24	-0.79	0	-1.53	-0.56	0	-0.5	0.13	0
PALA	-2.95	-2.95	-2.36	-0.25	0	0.08	0	-0.5	0.07	0
STEA	-2.81	-2.81	-2.43	0	0	-0.01	0	-0.5	0.13	0
SWIDE	0.28	0.28	-0.71	0	-0.5	-0.32	-0.11	-0.31	0.23	0

S4-6: 4XCT XP-Docking

ligand	GScore	DockScore	LipophilicEvdW	HBond	Electro	LowMW	EpikStatePenalty	Zpotr
GENPD	-6.65	-6.65	-1.04	-4.17	-1.14	-0.31	0	0
BELDF	-6.48	-6.43	-3.44	-1.97	-0.57	-0.5	0.04	0
IBELDF	-6.7	-6.28	-3	-1.62	-0.58	-0.5	0.42	-1
SWNIN	-6.17	-6.09	-3.43	-1.73	-0.51	-0.5	0.08	0
DTPA	-5.94	-5.94	-0.95	-1.23	-3.26	-0.5	0	0
SWCIN	-5.87	-5.83	-2.65	-1.41	-0.31	-0.5	0.04	-1
NSWIN	-5.69	-5.69	-2.95	-1.79	-0.45	-0.5	0	0
CHIRT	-5.69	-5.66	-2.65	-1.84	-0.71	-0.5	0.03	0
MANG	-5.63	-5.63	-2.02	-2.54	-0.97	-0.1	0	0
SWPU	-5.59	-5.55	-1.26	-3.75	-0.58	0	0.04	0
SWIDE	-5.4	-5.4	-0.99	-2.52	-1.58	-0.31	0	0
PALA	-5.39	-5.39	-1.82	0	-3.08	-0.5	0	0
AMARG	-5.5	-5.33	-1.41	-2.1	-0.99	0	0.17	-1
X9DDM	-5.68	-5.3	-2.15	-1.29	-0.74	-0.5	0.39	-1
DMBEDF	-5.63	-5.23	-2.75	-1.92	-0.47	-0.5	0.4	0
AMARSW	-5.3	-5.12	-1.97	-2.46	-0.87	0	0.17	0
GENTN	-4.89	-4.89	-2.81	-1.22	-0.36	-0.5	0	0
STEA	-4.69	-4.69	-1.14	0	-3.05	-0.5	0	0
SWOL	-4.42	-4.42	-2.44	-1.56	-0.34	-0.08	0	0
DIMBELD	-4.35	-4.33	-2.52	-0.96	-0.38	-0.49	0.03	0
18DDXN	-4.34	-4.27	-1.45	-1.63	-0.76	-0.5	0.07	0
DECU	-4.14	-4.11	-2.33	-0.8	-0.51	-0.49	0.03	0
ENCF	-3.83	-3.83	-1.35	-1.22	-0.77	-0.5	0	0
OLEA	-3.82	-3.82	-1.29	0	-3.03	-0.5	0	0
LUPE	-2.9	-2.9	-1.79	-0.82	-0.21	-0.08	0	0
SYRIN	-2.65	-2.65	-1.39	-0.48	-0.67	-0.11	0	0
CHIRT	-2.6	-2.6	-2.6	0	0.08	-0.08	0	0
KAIRT	-2.39	-2.39	-1.49	-0.58	-0.25	-0.08	0	0
SWONE	-2.35	-2.35	-2.27	0	0.01	-0.08	0	0
TARAXA	-2.01	-2.01	-1.88	0	-0.12	0	0	0
FRIED	-1.96	-1.96	-1.87	0	-0.01	-0.08	0	0

STIG	-1.93	-1.93	-1.84	0	0.03	-0.12	0	0
PICA	-1.86	-1.86	-1.9	0	0.04	0	0	0
16GBL	-1.84	-1.84	-1.17	0	-0.59	-0.08	0	0
URSA	-1.8	-1.8	-0.67	-0.35	-0.79	0	0	0
TARAX	-1.75	-1.75	-1.84	-0.64	-0.19	-0.08	0	0
OLNA	-1.67	-1.67	-1.77	0	0.09	0	0	0
CALED	-1.56	-1.56	-1.55	0	0.07	-0.08	0	0
ERTH	-1.53	-1.53	-1.57	0	0.06	-0.02	0	0
BSIT	-1.5	-1.5	-1.54	-0.7	-0.14	-0.12	0	0
HOPB	-1.37	-1.37	-1.19	0	-0.1	-0.08	0	0
BAMY	-1.32	-1.32	-1.22	-0.7	-0.33	-0.08	0	0

Supplementary file 5

Red dotted line boxes in S5 images highlight individual samples, labeled in the following order. The full, uncropped images are shown below their respective figures. Where the blots with full edges are not visible, alternative blot images have been provided.

S5-1: A549 cells western blot analysis

S5-1.1: EGFR

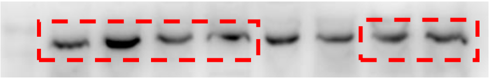

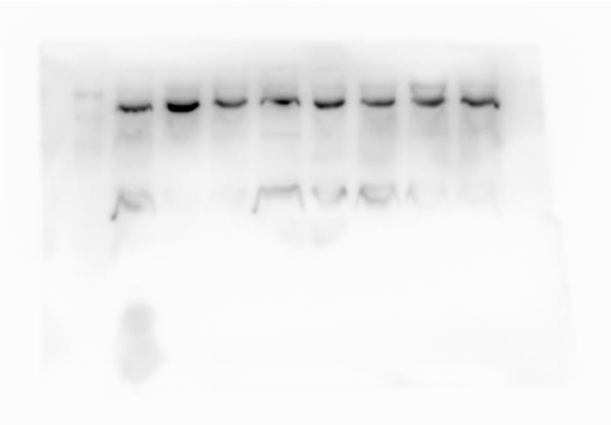

Blot 1: Lane 1: Normal cell, Lane 2: TGFb1, Lane 3: TGFb1+Nintedanib, Lane 4: Nintedanib, Lane 7: TGFb1+SC, and Lane 8: SC.

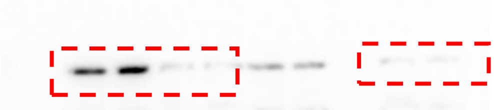

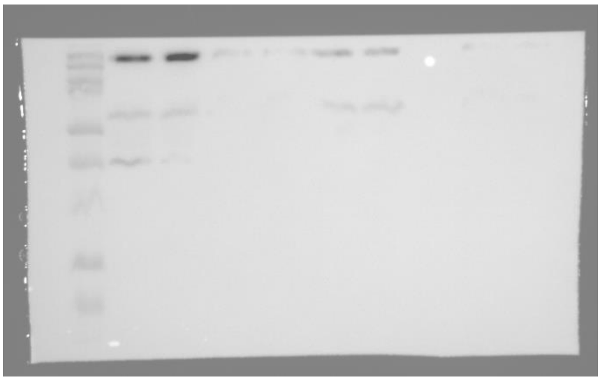

Blot 2: Lane 1: Normal cell, Lane 2: TGFb1, Lane 3: TGFb1+Nintedanib, Lane 4: Nintedanib, Lane 7: TGFb1+SC, and Lane 8: SC.

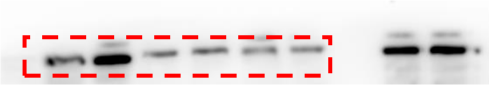

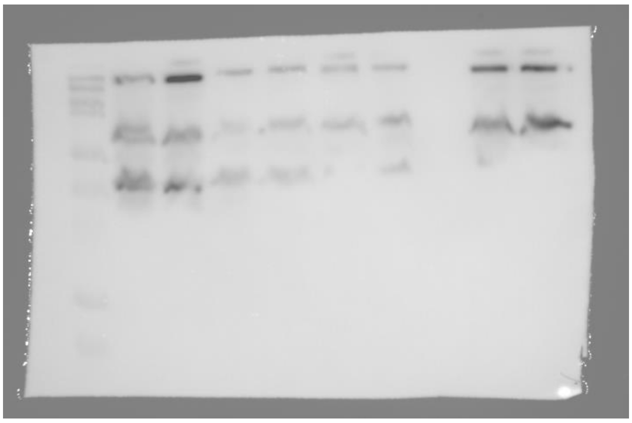

Blot 3: Lane 1: Normal cell, Lane 2: TGFb1, Lane 3: TGFb1+Nintedanib, Lane 4: Nintedanib, Lane 5: TGFb1+SC, and Lane 6: SC.

S5-1.2: NFkB

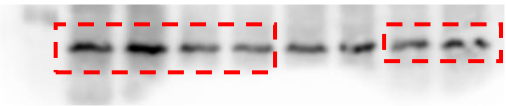

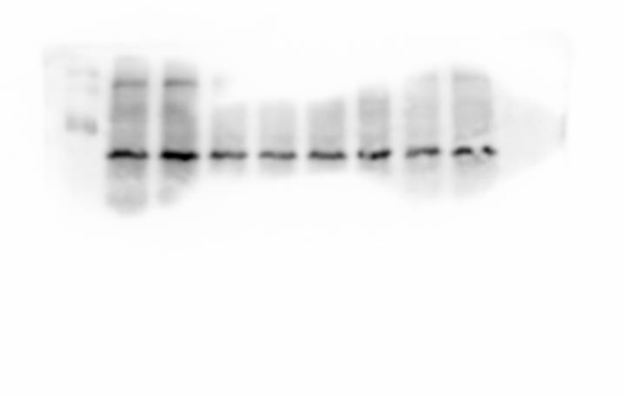

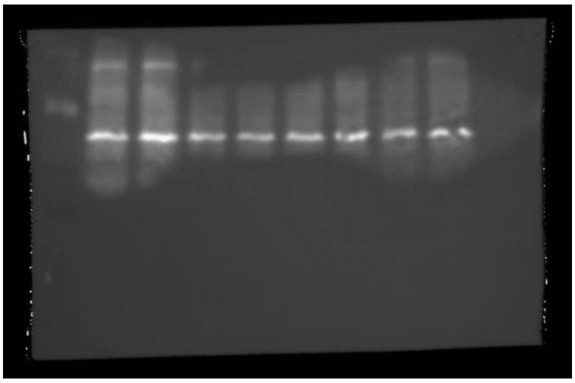

Blot 1: Lane 1: Normal cell, Lane 2: TGFb1, Lane 3: TGFb1+Nintedanib, Lane 4: Nintedanib, Lane 7: TGFb1+SC, and Lane 8: SC.

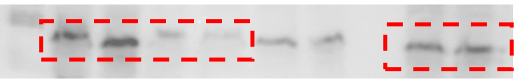

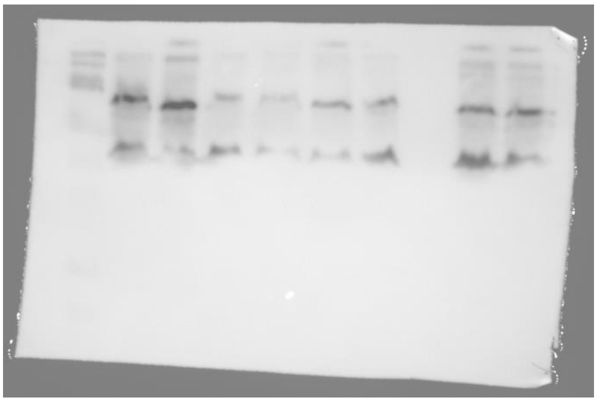

Blot 2: Lane 1: Normal cell, Lane 2: TGFb1, Lane 3: TGFb1+Nintedanib, Lane 4: Nintedanib, Lane 7: TGFb1+SC, and Lane 8: SC.

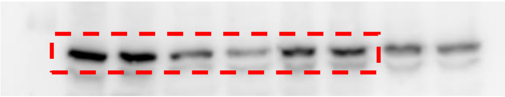

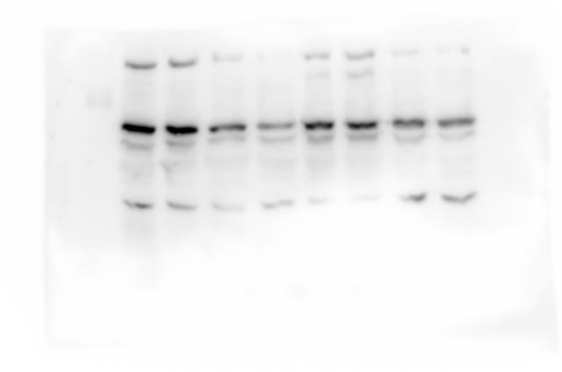

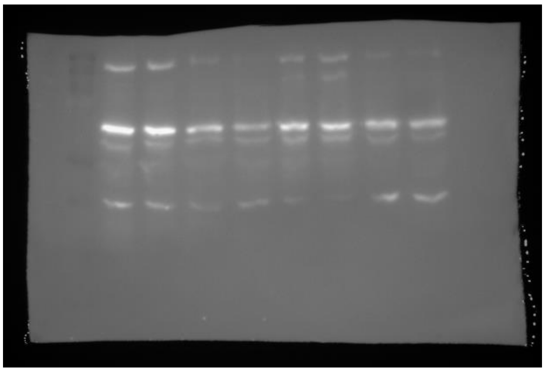

Blot 3: Lane 1: Normal cell, Lane 2: TGFb1, Lane 3: TGFb1+Nintedanib, Lane 4: Nintedanib, Lane 5: TGFb1+SC, and Lane 6: SC.

S5-1.3: p-NFkB

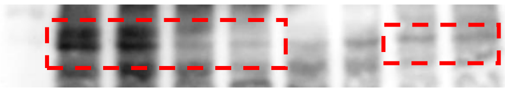

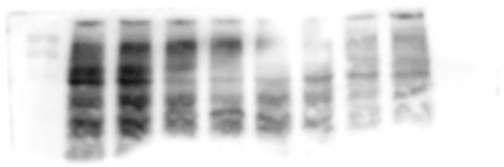

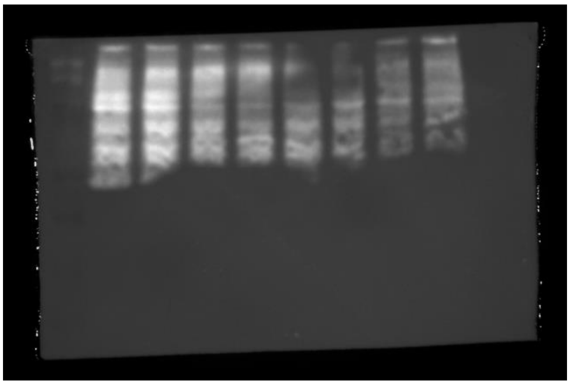

Blot 1: Lane 1: Normal cell, Lane 2: TGFb1, Lane 3: TGFb1+Nintedanib, Lane 4: Nintedanib, Lane 7: TGFb1+SC, and Lane 8: SC.

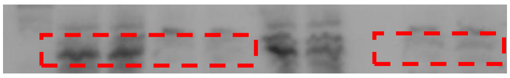

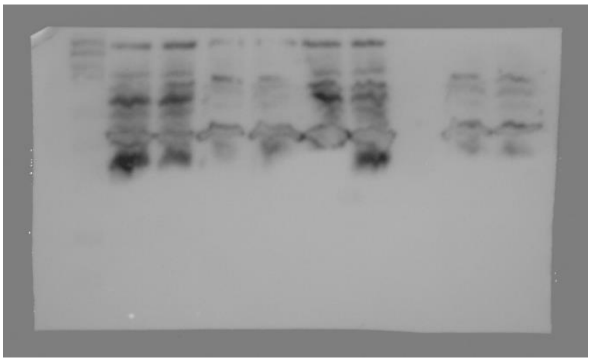

Blot 2: Lane 1: Normal cell, Lane 2: TGFb1, Lane 3: TGFb1+Nintedanib, Lane 4: Nintedanib, Lane 7: TGFb1+SC, and Lane 8: SC.

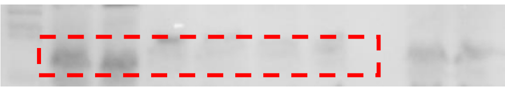

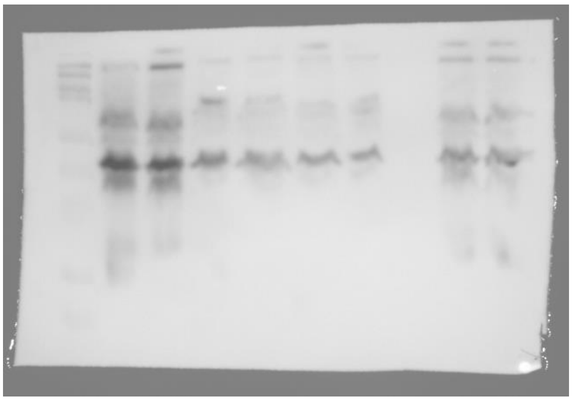

Blot 3: Lane 1: Normal cell, Lane 2: TGFb1, Lane 3: TGFb1+Nintedanib, Lane 4: Nintedanib, Lane 5: TGFb1+SC, and Lane 6: SC.

S5-1.4: β-actin

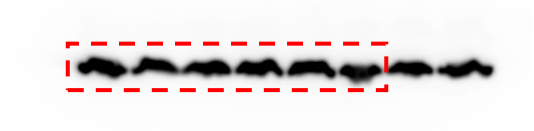

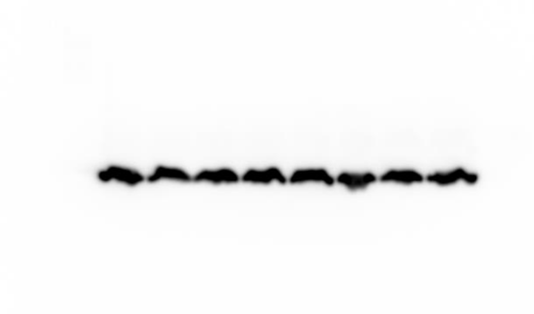

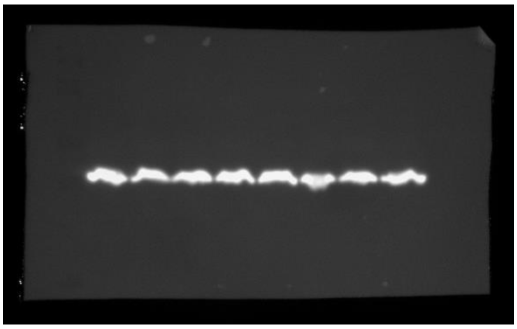

Blot: Lane 1: Normal cell, Lane 2: TGFb1, Lane 3: TGFb1+Nintedanib, Lane 4: Nintedanib, Lane 5: TGFb1+SC, and Lane 6: SC.

S5-2: NIH3T3 cells western blot analysis

S5-2.1: NFkB

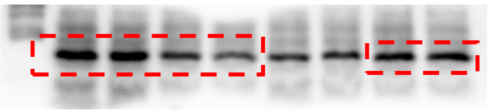

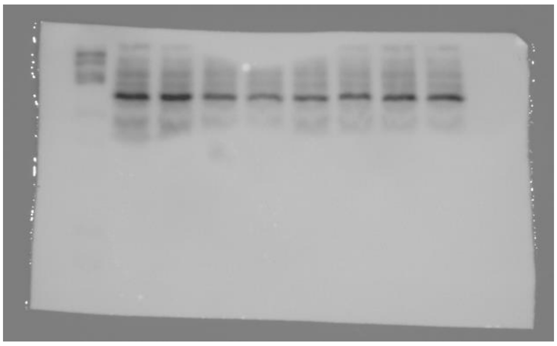

Blot 1: Lane 1: Normal cell, Lane 2: TGFb1, Lane 3: TGFb1+Nintedanib, Lane 4: Nintedanib, Lane 7: TGFb1+SC, and Lane 8: SC.

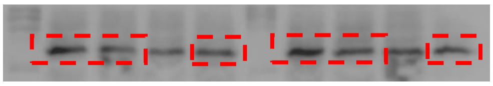

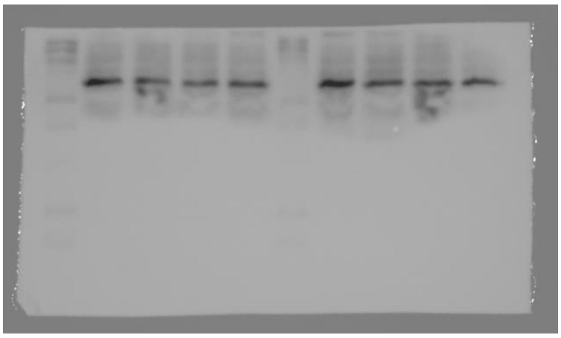

Blot 2: Lane 1: Normal cell, Lane 2: Nintedanib, Lane 4: SC, Lane 5: TGFb1, Lane 6: TGFb1+Nintedanib, and Lane 8: TGFb1+SC.

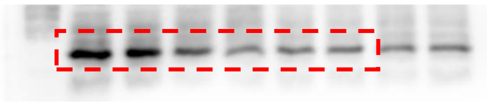

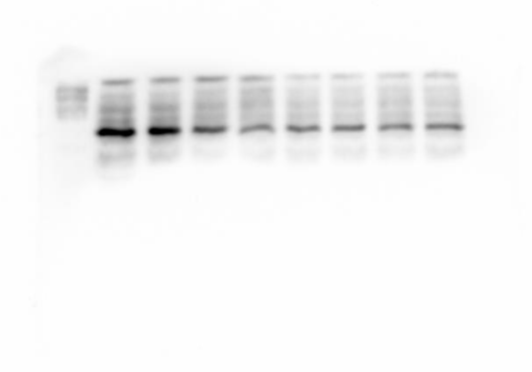

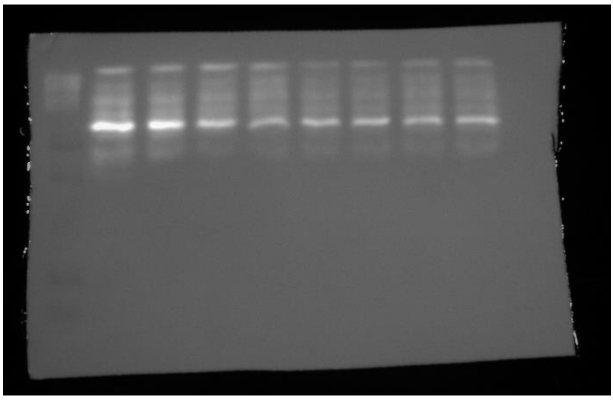

Blot 3: Lane 1: Normal cell, Lane 2: TGFb1, Lane 3: TGFb1+Nintedanib, Lane 4: Nintedanib, Lane 5: TGFb1+SC, and Lane 6: SC.

S5-2.2: p-NFkB

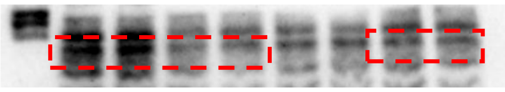

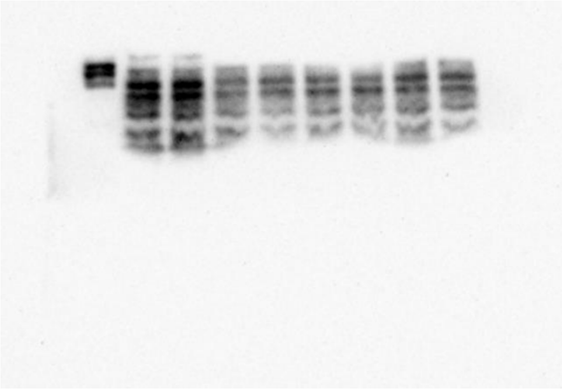

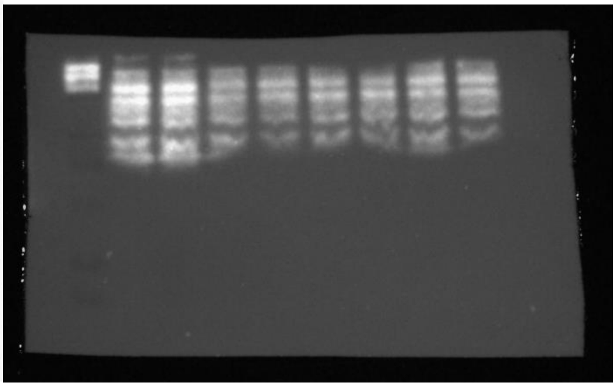

Blot 1: Lane 1: Normal cell, Lane 2: TGFb1, Lane 3: TGFb1+Nintedanib, Lane 4: Nintedanib, Lane 7: TGFb1+SC, and Lane 8: SC.

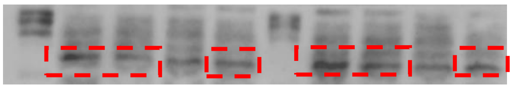

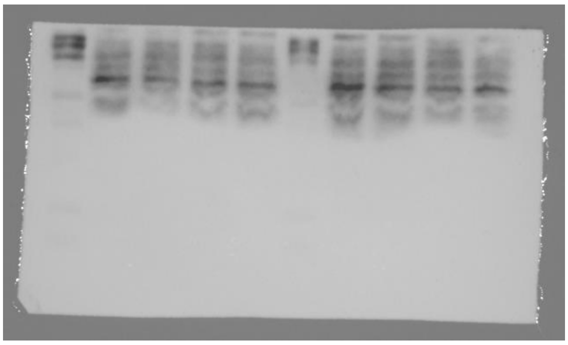

Blot 2: Lane 1: Normal cell, Lane 2: Nintedain, Lane 4: SC, Lane 5: TGFb1, Lane 6: TGFb1+Nintedanib, and Lane 8: TGFb1+SC.

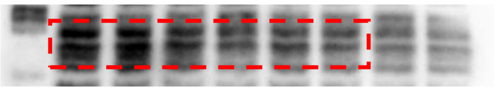

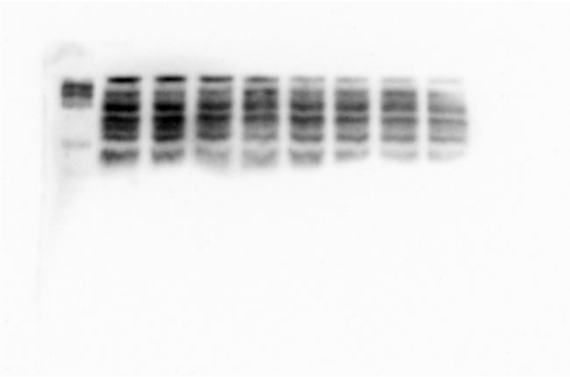

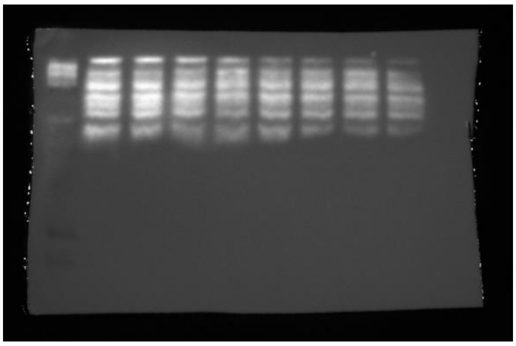

Blot 3: Lane 1: Normal cell, Lane 2: TGFb1, Lane 3: TGFb1+Nintedanib, Lane 4: Nintedanib, Lane 5: TGFb1+SC, and Lane 6: SC.

S5-2.3: β-actin

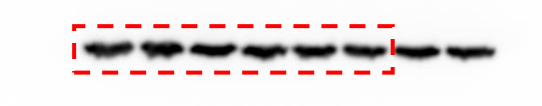

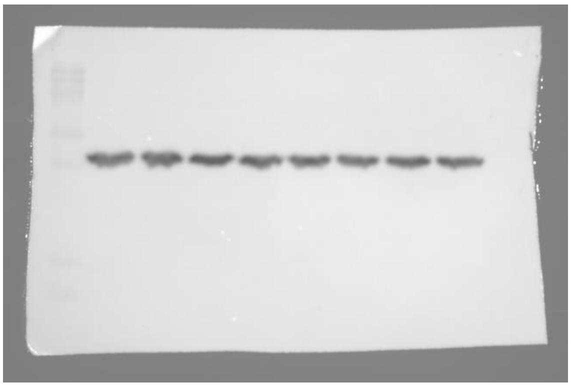

Blot: Lane 1: Normal cell, Lane 2: TGFb1, Lane 3: TGFb1+Nintedanib, Lane 4: Nintedanib, Lane 5: TGFb1+SC, and Lane 6: SC
